# Role of NCKAP1 in the Defective Phagocytic Function of Microglia-Like Cells Derived from Rapidly Progressing Sporadic ALS

**DOI:** 10.1007/s12035-023-03339-2

**Published:** 2023-05-08

**Authors:** Min-Young Noh, Min-Soo Kwon, Ki-Wook Oh, Minyeop Nahm, Jinseok Park, Young-Eun Kim, Chang-Seok Ki, Hee Kyung Jin, Jae-sung Bae, Seung Hyun Kim

**Affiliations:** 1 Department of Neurology, College of Medicine, Hanyang University, Wangsimniro 222-1, Seoul, 04763 Republic of Korea; 2 Department of Pharmacology, Research Institute of Basic Medical Science, School of Medicine, CHA University, CHA Bio Complex, 335 Pangyo, Gyeonggi-Do 13488 Republic of Korea; 3 Dementia Research Group, Korea Brain Research Institute, Daegu, Republic of Korea; 4 Department of Laboratory Medicine, College of Medicine, Hanyang University, Wangsimniro 222-1, Seoul, 04763 Republic of Korea; 5 GC Genome Corporation, Yongin, 16924 Republic of Korea; 6 KNU Alzheimer’s Disease Research Institute, Kyungpook National University, Daegu, 41566 Republic of Korea; 7 Department of Laboratory Animal Medicine, College of Veterinary Medicine, Kyungpook National University, Daegu, 41566 Republic of Korea; 8 Department of Physiology, Cell and Matrix Research Institute, School of Medicine, Kyungpook National University, Daegu, 41944 Republic of Korea; 9 Department of Biomedical Science, BK21 Plus KNU Biomedical Convergence Program, Kyungpook National University, Wangsimniro 222-1, Daegu, 41944 Republic of Korea; 10 Cell Therapy Center, Hanyang University Hospital, Wangsimniro 222-1, Seoul, 04763 Republic of Korea

**Keywords:** Amyotrophic Lateral Sclerosis, Microglia, NCKAP1, Phagocytosis

## Abstract

Microglia plays a key role in determining the progression of amyotrophic lateral sclerosis (ALS), yet their precise role in ALS has not been identified in humans. This study aimed to identify a key factor related to the functional characteristics of microglia in rapidly progressing sporadic ALS patients using the induced microglia model, although it is not identical to brain resident microglia. After confirming that microglia-like cells (iMGs) induced by human monocytes could recapitulate the main signatures of brain microglia, step-by-step comparative studies were conducted to delineate functional differences using iMGs from patients with slowly progressive ALS [ALS(S), *n* = 14] versus rapidly progressive ALS [ALS(R), *n* = 15]. Despite an absence of significant differences in the expression of microglial homeostatic genes, ALS(R)-iMGs preferentially showed defective phagocytosis and an exaggerated pro-inflammatory response to LPS stimuli compared to ALS(S)-iMGs. Transcriptome analysis revealed that the perturbed phagocytosis seen in ALS(R)-iMGs was closely associated with decreased *NCKAP1* (NCK-associated protein 1)-mediated abnormal actin polymerization. NCKAP1 overexpression was sufficient to rescue impaired phagocytosis in ALS(R)-iMGs. Post-hoc analysis indicated that decreased *NCKAP1* expression in iMGs was correlated with the progression of ALS. Our data suggest that microglial NCKAP1 may be an alternative therapeutic target in rapidly progressive sporadic ALS.

**Supplementary Information:**

The online version contains supplementary material available at 10.1007/s12035-023-03339-2.

## Background

Amyotrophic lateral sclerosis (ALS) is a fatal neurodegenerative disease characterized by loss of motor neurons and inflammation in the motor neural axis, including the primary motor cortex, brainstem, and spinal cord. It results in muscle weakness, wasting, respiratory paralysis, and, ultimately, death within 3 – 5 years [1].

The clinical progression of ALS is highly variable. Microglial cells play a crucial role in determining the clinical progression of ALS [2]. The dual roles of microglia (i.e., protective or toxic effect according to disease stage) have been thoroughly described in a *SOD1* mutant mouse model [3, 4]. However, most ALS cases are sporadic (sALS), and results from the SOD1 mutant mouse model are inadequate to describe microglial function in sporadic ALS. Moreover, reactive microglial phagocytosis exhibits a neuroprotective effect rather than exacerbating motor neuron death in a model of *TDP-43* proteinopathy, the most common pathology found in sALS, in contrast to a SOD1 mutant mouse model [5, 6]. Furthermore, the results of previous clinical trials that looked at the use of anti-inflammatory drugs in ALS suggest that complete suppression of microglial function seems to be irrelevant to optimal control of the neuro-inflammatory process [7, 8]. These findings suggest that the nature of neuroinflammation is not uniform across pathological conditions. It appears to be contingent on the type of stimulus, its duration, and the regions involved [9]. In addition, not only non-neuronal cells such as microglia [10] and astrocytes [11], but also peripheral monocytes contribute to the inflammatory process and immune dysfunction in ALS [2, 12]. Recent evidence suggests that peripheral monocytes contribute to the speed of ALS progression [13] and that modulating their inflammatory activity can ameliorate murine ALS [14, 15]. These findings provide important insights into the process of neuroinflammation, which is induced by a complex integrated form not just a separated manner.

Recent studies have shown that microglia can be divided into diverse microglial subtypes, depending on the surrounding inflammatory milieu or their origin [16]. Despite the importance of understanding the role of each microglial subpopulation based on single-cell assays in ALS, it is practically impossible to assess the overall functional status of in situ microglia and other cells that concomitantly participate in the complex inflammatory process in living ALS patients based from single-cell assays. Although induced pluripotent stem cells (iPSCs) and a co-cultured model have been used to study microglia [17–19], iPSC-derived microglia-like cells have drawbacks; for example, they do not reflect current pathological status due to rejuvenation [20]. In addition, they require intricate procedures with low reprogramming efficiency and take a long time to grow and study [21–23]. On the other hand, microglia-like cells induced from monocytes (iMGs) have an advantage in that they mirror the current pathophysiological state of CNS phagocytes [22, 24, 25]. Although they are not identical to yolk sac-derived microglia, iMGs can be used as a model system to interpret human microglial pathology in living patients, at least to some extent [21, 24–26].

Microglia reaction results in sometimes unexpecting and contrasting outcomes, depending on stage of disease and current pathological insult. Thus, therapeutic strategies targeting microglia should be tailored to their affected functional pathways rather than diverse molecular signatures and phenotypes in specific disease status. Our a priori assumption was that the progression speed of ALS might be associated with the microglia phagocytic function, which was indirectly suggested by animal model and human data [5, 10, 27]. Our hypothesis is that microglia-related inflammatory processes and their main functions differ according to the speed of progression of ALS. After establishing the iMGs model using peripheral blood monocytes (PBMC) described in previous studies [21, 24, 25], the reliability of our iMGs was confirmed again by demonstrating that iMGs share major subsets of genes that are related to innate immune functions of microglia in living ALS patients. After that, serial comparative studies delineating the molecular and functional differences in iMGs, including gene expression and phagocytic function, were conducted in slowly and rapidly progressive patients with ALS. Phagocytic dysfunction was remarkable in iMGs of ALS(R). By transcriptomic analysis, we found a target molecule involved in the phagocytic dysfunction by rapid progression. Finally, we identified a molecular target associated with the defective phagocytic function of iMGs in ALS.

## Methods

### Study Design and Sample Preparation

We designed the current study to find key factors related to microglial functional differences according to ALS progression speed using a microglia-like cell model (iMGs) as a translational research tool. First, the iMG model was shown to exhibit the signature gene patterns of brain microglia and the innate functions of microglia in healthy donors. To further validate the model, we compared iMGs to brain microglia that were obtained from the same ALS patient. Thereafter, we conducted a systematic comparative analysis to delineate the different natures of iMGs in ALS patients according to speed of clinical progression. All participants had clinically definite, clinically probable, or clinically probable with a laboratory-supported diagnosis according to the revised El Escorial criteria [28]. The patients’ clinical information, including ALS Functional Rating Scale–Revised (ALSFRS-R) score (0 ~ 48), was prospectively registered in the database. Progression speed was expressed as delta FS (∆FS) [29], (i.e., (48—ALSFRS-R score at the time of diagnosis)/(duration from onset to diagnosis in months)). We enrolled 29 participants with ALS and five healthy volunteers between September 2015 and July 2017. We tentatively sub-grouped participants who exhibited extremely rapid or slow progression in the ALS/MND Clinic database at Hanyang University Hospital as having rapidly progressive ALS [ALS(R); ∆FS ≥ 1.0, *n* = 15] and slowly progressive ALS [ALS(S); ∆FS < 0.5, *n* = 14] [30, 31]. After obtaining informed consent, blood samples were collected to generate iMGs, and comparative studies were serially conducted. Second, we endeavored to identify target molecules related to the functional properties of microglia that are present only in ALS(R)-iMGs. To do this, we compared transcriptome data between ALS(R)-iMGs and ALS(S)-iMGs. Subsequently, we conducted functional studies on an identified target molecule. All participants’ medical records were reviewed, and their clinical characteristics are summarized in Supplemental Table 1. We excluded participants with familial history or pathogenic variants of ALS-related genes in whole-exome sequencing to exclude possible genetic effects on ALS progression (Supplemental Table 2). This study was conducted under the World Medical Association’s Declaration of Helsinki and approved by the Ethics Committee of Hanyang University Hospital (HYUH IRB 2013–06-012, 2017–01-043). All patients provided written informed consent before inclusion in the study.

### Establishment of Induced Microglia-Like Cells (iMGs) from PBMCs

PBMCs were isolated by density gradient centrifugation using Ficoll (GE Healthcare, Uppsala, Sweden). iMG cells were established using a previously published method [25]. Briefly, PBMCs were resuspended in RPMI-1640 (Gibco, Carlsbad, CA, USA) containing 10% fetal bovine serum (FBS; Gibco) and 1% antibiotic/antimycotic (Invitrogen, Carlsbad, CA, USA) and cultured overnight at 37 °C and 5% CO_2_. The next day, adherent cells (monocytes) were cultured in RPMI-1640 Glutamax (Gibco) supplemented with 1% antibiotic/antimycotic, recombinant granulocyte–macrophage colony-stimulating factor (GM-CSF) (R&D Systems), and recombinant IL-34 (IL-34) (R&D Systems) to develop iMG cells. After generating iMGs, the cells were labeled with human CD11b-APCVio770 and CD45-phycoerythrin (PE) (Miltenyi Biotec, Gladbach, Germany), and flow cytometry was performed as described previously [32]. All data were assessed by FACSCanto II flow cytometry (BD Biosciences, Piscataway, NJ, USA) and analyzed by FlowJo software. Gene expression in iMGs was measured using quantitative real‑time polymerase chain reaction (qRT‑PCR) as described previously [32]. Primer information is shown in Supplemental Table 3.

### Immunostaining and Morphology Analysis

To visualize iMGs, the cells were fixed and stained with a microglial marker (IBA1) and counterstained with DAPI (4′,6-diamidino-2-phenylindole). Immunostaining was performed as previously described [32]. Antibody information is shown in Supplemental Table 3. Images were acquired by confocal microscopy (TCS SP5, Leica, Wetzlar, Germany). Three-dimensional reconstructions of randomly selected iMG cells (IBA1-positive) were generated using Imaris software (Bitplane, Zurich, Switzerland). Two blinded researchers performed morphometric analysis of each reconstructed cell after determining dendrite length, the number of segments, and branch points [33].

### Phagocytosis Assay

iMGs were treated with 4 µl of red fluorescent latex beads for 24 h at 37 °C. The cells were washed twice with ice-cold phosphate-buffered saline (PBS), fixed, and stained with a microglial marker (P2RY12) and DAPI. The number of phagocytized beads was counted using ImageJ software [34]. To assess phagocytosis cup formation in iMGs, cells were incubated with latex beads for 2 h, fixed, and stained with fluorescent phalloidin (1: 1,000; Molecular Probes, Eugene, OR, USA) for 45 min with secondary antibodies. Antibody information is shown in Supplemental Table 3. Images were acquired by confocal microscopy. For live-cell imaging, iMGs were grown in Lab-Tek II Chamber Slide (Thermo Fisher Scientific, Rochester, NY, USA) and labeled with 100 nM SiR-actin dye (Cytoskeleton Inc., Denver, CO, USA) according to the manufacturer’s protocol. Beads (1.1 μm, Sigma-Aldrich, Saint Louis, MO, USA) were added to the cells before analysis. Images were captured one frame every 1 min 30 s over 5 h using a DeltaVision Imaging System (Applied Precision, Bratislava, Slovakia).

### Isolation of Human Brain Microglia from the Neural Tissue of sALS Patient

We confirmed the microglia signature of iMGs that originated from PBMC from an sALS patient whose blood sample was collected just 1 day before death. We immediately isolated microglia from fresh brain tissue (brain-MG) from the same patient. The patient had no known pathogenic mutations, including *FUS*, *C9orf72*, *SOD1*, *ALS2*, *SPG11*, *UBQLN2*, *DAO*, *GRN*, *SQSTM1*, *SETX*, *MAPT*, *TARDBP*, or *TAF15* gene mutations. For brain-MG culture, the immediately obtained fresh middle temporal gyrus was washed in HBSS. The tissue was then diced into ~ 1-mm^3^ pieces using a sterile scalpel and transferred to a 50-ml falcon tube containing 10 ml enzyme dissociation mixture with 10 U/ml DNase (Invitrogen) and 2.5 U/ml papain (Worthington, NJ, USA) in Hibernate-A medium (Gibco) (per gram of tissue). The mixture was incubated at 37 °C for 10 min with gentle rotation. The tissue was removed from the incubator, gently triturated to aid digestion, and returned to the incubator for a further 10 min. Dissociation was slowed by adding equal volumes of Dulbecco’s modified Eagle medium and F-12 medium (DMEM/F12; Gibco) with 1% B27 (Gibco). The cell suspension was passed through a 70-μm cell strainer (Bector Dickinson, NJ, USA). Cells were centrifuged at 160 × *g* for 10 min. The supernatant was discarded, and the cell pellet was resuspended in 20 ml DMEM/F12 with 1% B27, 1% GlutaMAX (Gibco), and 1% penicillin–streptomycin-glutamine (PSG; Gibco). Next, one-third volume of cold Ficoll (GE Healthcare, Little Chalfont, UK) was added to the cell suspension, and the tube was centrifuged at 4000 rpm for 30 min at 4 °C. The interphase containing the microglia was transferred to a new tube (the myelin and erythrocyte layers were discarded) and washed twice with DMEM supplemented with 10% FCS, 1% Pen/Strep, 1% gentamycin, and 25 mM HEPES (Invitrogen). Negative selection of granulocytes (previous method only) and positive selection of microglia with anti-CD15- and anti-CD11b-conjugated magnetic microbeads (Miltenyi Biotec), respectively, were performed by magnetic activated cell sorting (MACS) according to the manufacturer’s protocol [35]. Briefly, cells were incubated with 10 μl CD15 microbeads for 15 min at 4 °C, washed, suspended in bead buffer (0.5% BSA, 2 mM EDTA in PBS, pH 7.2), and transferred to an MS column placed in a magnetic holder. The flow-through containing unlabeled cells were collected, washed, and incubated with 20 μl CD11b microbeads for 15 min at 4 °C. The cells were then washed and placed on a new MS column in a magnetic holder. The CD11b^+^ cell fraction was eluted by removing the column from the magnet, adding bead buffer, and emptying the column with a plunger. Acutely isolated primary microglia were suspended in Trizol reagent (Invitrogen) and stored at − 80 °C.

We isolated monocytes from the blood of the same patient using anti-CD14-conjugated magnetic microbeads (Miltenyi Biotec) according to the manufacturer’s protocol. The isolated monocytes were suspended in Trizol reagent (Invitrogen) and stored at − 80 °C for RNA-seq and qRT‑PCR.

### RNA Sequencing and Data Analysis

RNA quality was assessed with an Agilent 2100 bioanalyzer using an RNA 6000 Nano Chip (Agilent Technologies, Amstelveen, Netherlands). RNA libraries were constructed using a SENSE 3′ mRNA-Seq Library Prep Kit (Lexogen, Inc., Vienna, Austria) according to the manufacturer’s instructions. High-throughput sequencing was performed as single-end 75 sequencings using a NextSeq 500 platform (Illumina, Inc., San Diego, CA, USA). SENSE 3′ mRNA-Seq reads were aligned using Bowtie2 version 2.1.0 [36]. Differentially expressed genes (DEGs) were determined based on counts from unique and multiple alignments using EdgeR in R version 3.2.2 and BIOCONDUCTOR version 3.0 [37]. Read count data were processed based on the global normalization method using Genowiz™ version 4.0.5.6 (Ocimum Biosolutions, India). Gene classification was based on DAVID (http://david.abcc.ncifcrf.gov/) database searches. MeV 4.9.0 was used for clustering samples and genes and visualization.

### Cell Culture and Transfection

HeLa cells were cultured in Dulbecco’s MEM containing 10% FBS, sodium bicarbonate, sodium pyruvate (Sigma-Aldrich), and antibiotics. HeLa cells were transfected with green fluorescent protein (GFP)-tagged human NCKAP1 cDNA or NCKAP1 shRNA using Lipofectamine 2000 (Invitrogen) according to the manufacturer’s protocol. iMGs were transduced with pLenti-C-mGFP-Human NCKAP1 (NM_013436), the cDNA ORF Clone (OriGene Technologies, Rockville, MD, USA), or pGFP-C-shLenti-NCKAP1 Human shRNA lentiviral particles (ID 10,787) according to the manufacturer’s protocol. After transfection, cells were assessed by western blotting and RT-qPCR as previously described [32]. Antibody and primers information are shown in Supplemental Table 3.

### Enzyme-Linked Immunosorbent Assay

Secretion of pro- and anti-inflammatory cytokines (TNF-α, IL-1β, IL-6, IL-10, and TGF-β1) during LPS stimulation from culture supernatants was tested using a commercially available cytokine assay kit obtained from Millipore (Billerica, MA), according to the manufacturer’s protocol. Human IL-6, interferon (IFN)-γ, IL-8, TNF-α, and CCL2/MCP-1 (R&D Systems) were used to determine cytokine concentration in plasma samples of patients with ALS and healthy controls according to the manufacturer’s instructions. Each assay was performed in triplicate.

### Statistical Analysis

Data are presented as either mean ± SD or SEM. Normality was accessed by Shapiro–Wilk and Kolmogorov–Smirnov normality tests. For normal distribution, comparisons between groups were performed using the unpaired *t* test or paired *t* test (two-tailed) while multiple comparisons were performed using ordinary one-way ANOVA followed by Tukey’s multiple comparisons test (to compare multiple treatment groups versus control). For non-normal distribution, the Mann–Whitney *U* test (two-tailed) was used to compare between groups. For non-normal multiple comparisons, a Kruskal–Wallis one-way ANOVA (non-parametric) followed by Dunn’s multiple comparisons test was performed (to compare multiple treatment groups versus controls). Correlations were analyzed by Spearman rank correlation test. All statistical analyses were performed using Prism 9 (GraphPad Software, San Diego, CA, USA). *P < 0.05 was considered statistically significant.

## Results

### Healthy Control (HC)-iMGs Present the Main Signatures of Microglia and Show Intrinsic Functions

**Fig. 1:**
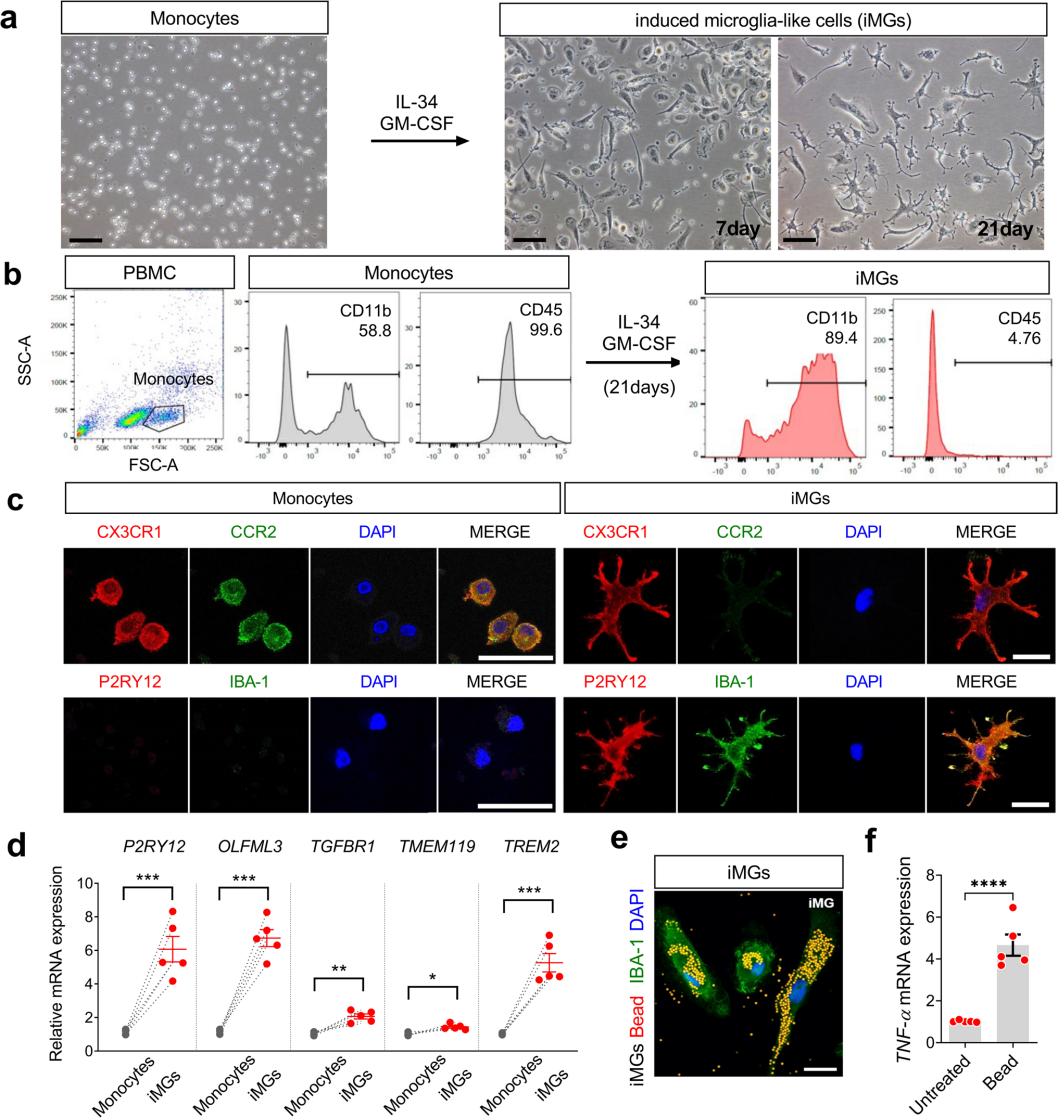
Characterization of iMGs for subsequent experiments using the iMGs model. **a** Representative image of monocytes and iMGs in different stages, taken on days 7 and 21. Scale bar: 50 μm. **b** Flow cytometry plots showing microglia (CD45^low^ CD11b.^+^) from iMGs generated by treating monocytes with GM-CSF and IL-34 for 21 days. **c** Confocal images of microglial markers P2RY12 (green), IBA-1 (green), monocytes marker CCR2 (green), and DAPI counterstain (blue) in HC-iMGs. Scale bar: 25 μm. **d** The relative mRNA expression of microglial signature genes (*P2RY12, OLFML3, TGFBR1, TMEM119*, and *TREM2*) in HC-iMGs by qRT-PCR. Each dot represents data from individual-subject-derived HC-iMGs and monocytes (*n* = 5). **e** Phagocytic activity of HC-iMGs (IBA-1: green) incubated with fluorescent latex beads (red) for 24 h and immunostained with P2RY12, as iMG markers (green) with DAPI counterstain (blue). Scale bar: 25 μm. **f** After bead phagocytosis for 72 h, *TNF-α* mRNA expression in iMGs. Each dot represents data from individual-subject-derived HC-iMGs and monocytes (*n* = 5). Values are means ± SEM. Comparisons were made against control (*P < 0.05, **P < 0.01, ***P < 0.001, ****P < 0.0001; unpaired *t* test or paired *t* test)

For generating human iMGs, we obtained PBMCs from five healthy controls (HCs) and treated them with GM-CSF (10 ng/mL) and IL-34 (100 ng/mL) for 21 days to induce microglia-like cell branched morphology (iMGs) (Fig. 1a). The cytokine cocktail shifted the cell population toward CD11b^+^ CD45^low^ cells by flow cytometry analysis (Fig. 1b). In immunofluorescence analysis, HC-iMGs were characterized by upregulated expression of resident microglia surface markers including P2RY12 and IBA-1 and disappeared monocyte marker CCR2 (Fig. 1c). In qRT‑PCR analysis, resident microglial signature genes, *P2RY12*, *OLFML3*, *TGFBR1*, *TMEM119*, and *TREM2*, were upregulated in iMGs compared to monocytes (Fig. 1d). In addition, HC-iMGs showed normal phagocytic function and increased expression of *TNF-α* mRNA upon stimulation with latex beads (Fig. 1e and f), consistent with previous studies that iMGs could recapitulate the main signatures of microglia [21].

### iMGs Express Key Genetic Signature of Brain Microglia

Although our data and the results of previous studies [21, 24] suggest that HC-iMGs present key signatures of microglia and show intrinsic microglial functions, it is yet unclear whether iMGs derived from PBMCs accurately reflect brain microglia in the same person. To address this question, we obtained fresh brain tissue and peripheral blood from an sALS patient just before death who consented to the body being donated and immediately isolated microglial cells from autopsy brain tissue using CD11b beads.

**Fig. 2:**
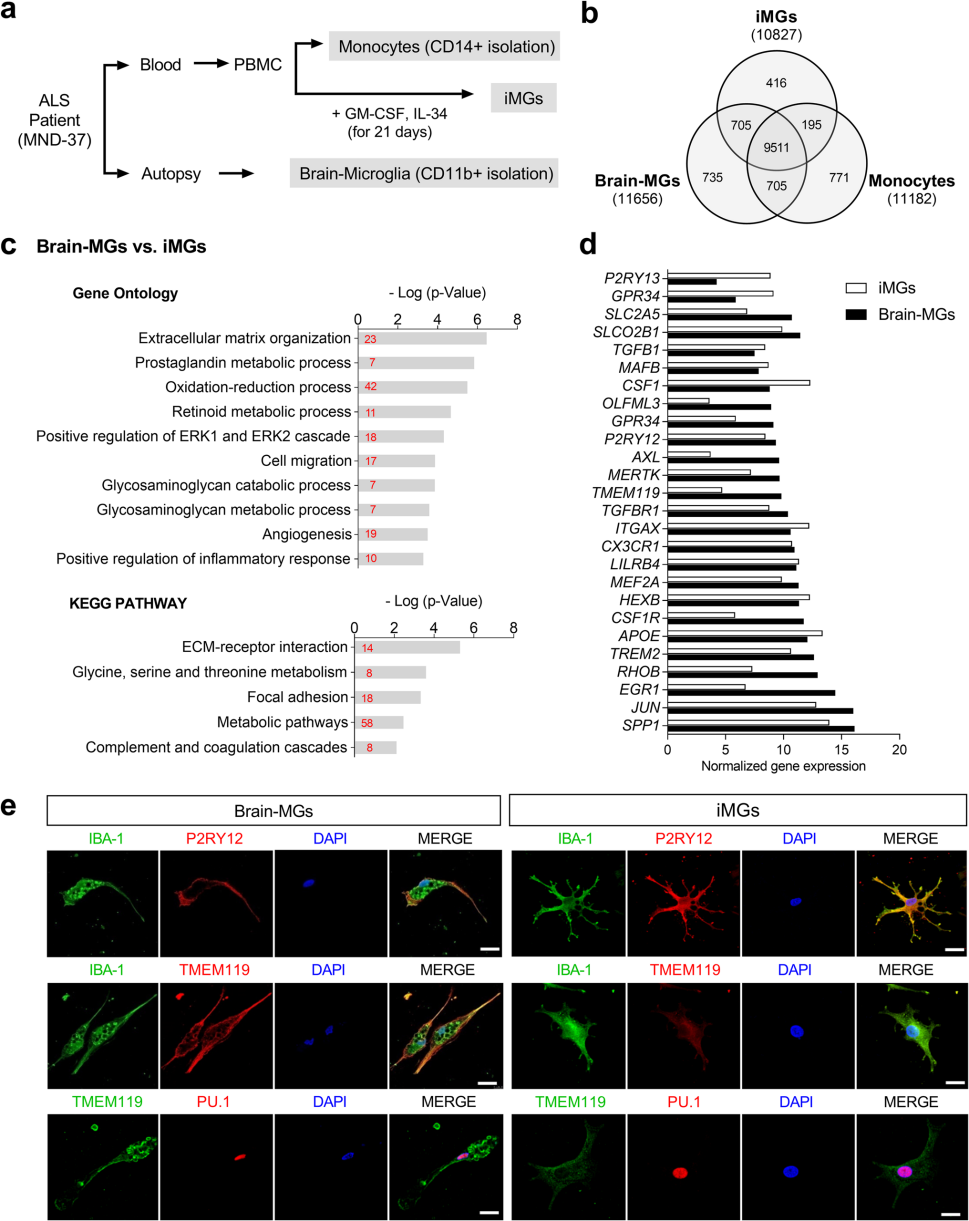
Transcriptome comparison between iMGs and brain microglia obtained simultaneously from a single ALS patient. **a** Schematic representation of the experimental procedure used to compare iMGs with brain microglia (brain-MGs, CD11b^+^ isolation) and monocytes (CD14^+^ isolation) in an ALS patient. **b** Venn diagram showing unique and intersecting genes (13,038) that are differentially expressed (DE) in monocytes, iMGs, and brain-MGs according to RNA-seq (fold change >|3|). **c** GO analysis of ten significant pathway modules and five KEGG pathway modules in the 705 genes shared between iMGs and brain-MGs. The number within each bar indicates the number of genes in the database for the specified term. **d** Bar graphs of microglial-specific or -enriched genes measured in iMGs and brain-MG as [log^2^ (FPKM + 1)] presented as mean ± SEM. **e** Confocal image of microglial markers such as IBA-1 (green), P2RY12 (red), TMEM119 (red/green), PU.1 (red), and DAPI counterstain (blue). The figure is representative of independent experiments performed in replicates (*n* = 10). Scale bar: 25 μm

To identify similarities between iMGs and brain microglia, we performed immunostaining and RNA sequencing (RNA-seq) of iMGs, brain microglia (brain-MGs, CD11b +), and monocytes (CD14 +) (Fig. 2a). DEGs analysis revealed 13,038 genes that had a greater than threefold difference in either iMGs or brain-MGs compared to monocytes. Of these, 705 (5.4%) overlapped between iMGs and brain-MG (Fig. 2b, Supplemental Table 4). However, only 195 (1.5%) of the enriched genes overlapped between iMGs and monocytes. The identified the 705 overlapping genes between iMGs and brain-MG were uploaded to the DAVID software for Gene ontology (GO) and Kyoto Encyclopedia of Genes and Genomes (KEGG) pathway analyses. The results of the GO analysis revealed that they were significantly enriched in biological processes, including “extracellular matrix organization,” “prostaglandin metabolic process,” “oxidation–reduction processes,” “cell migration,” and “positive regulation of inflammatory responses” (Fig. 2c, Supplemental Table 5). KEGG pathway analysis revealed that they were highly associated with pathways including “ECM-receptor interaction,” “glycine, serine, and threonine metabolism,” “focal adhesion,” “metabolic pathways,” and “complements and coagulation cascades” (Fig. 2c, Supplemental Table 5). Regarding microglial signature genes [38], the expression levels of *SPP1*, *JUN*, *TREM2*, *APOE*, *HEXB*, *MEF2A*, *LILRB4*, *CX3CR1*, *ITGAX*, *TGFBR1*, *P2RY12*, *MAFB*, *TGFB1*, and *SLCO2B1* in iMGs were similar to those in brain-MG, although the expression levels of *OLFML3*, *AXL*, *CSF1R*, *RHOB*, *EGR1*, and *TMEM119* in iMGs were relatively low (Fig. 2d). Immunofluorescence staining showed that P2RY12, IBA-1, and the transcription factor PU.1 were well preserved in both iMGs and brain-MG, although immunoreactivity (IR) of TMEM119 in iMGs was less than in brain-MG (Fig. 2e).

### ALS(R)-iMGs Show Dystrophic Morphology and Severely Impaired Phagocytic Function

To verify our hypothesis that microglial dysfunction was associated with the speed of progression of ALS, we enrolled 29 participants with ALS and five HCs and tentatively sub-grouped 29 ALS patients between extremely rapidly progressive ALS [ALS(R); ∆FS ≥ 1.0, *n* = 15] or slowly progressive ALS [ALS(S); ∆FS < 0.5, *n* = 14].

**Fig. 3:**
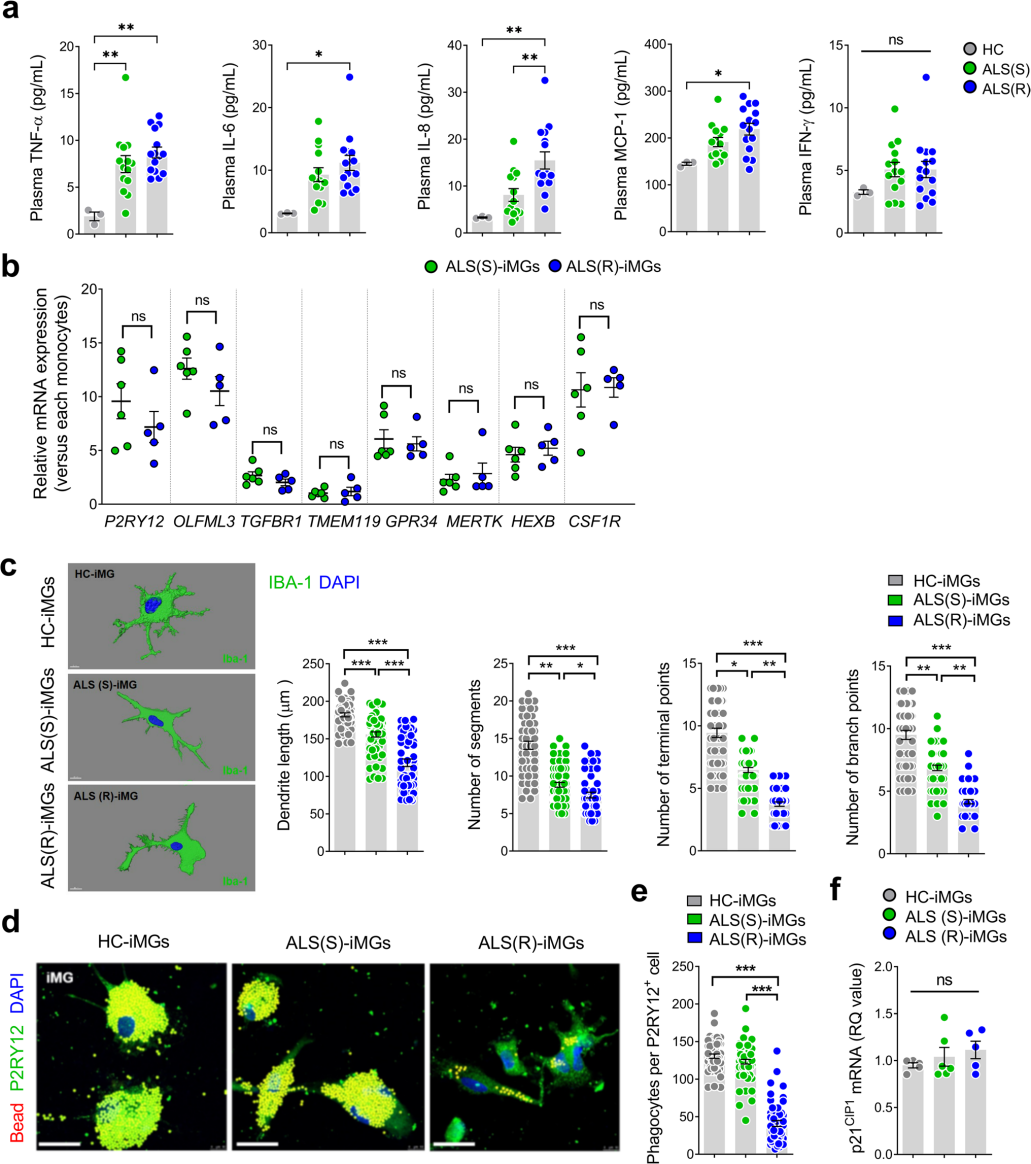
Impaired phagocytic function of ALS(R)-iMGs. **a** Plasma cytokine levels in the 29 ALS patients (rapidly progressive ALS [ALS(R); ∆FS ≥ 1.0, *n* = 15] or slowly progressive ALS [ALS(S); ∆FS < 0.5, *n* = 14]) and HCs (*n* = 3). **b** The mRNA expression of microglial signature genes from ALS(R)-iMGs (*n* = 5) and ALS(S)-iMGs (*n* = 6) by RT-qPCR. Each dot represents data from individual-subject-derived iMGs. **c** Imaris-based morphometric analysis comparing the three groups’ iMGs (IBA-1; green). Each column represents the mean of each group’s iMGs with at least ten randomly selected cells per subject-derived iMGs. **d** Confocal images of bead phagocytosis in the three groups’ iMGs fed with red-fluorescence bead by immunostaining with P2RY12 (green) and DAPI counterstain (blue). Scale bar: 25 μm. **e** Quantification of phagocytosed by the number of beads per P2RY12-positive cell. Each data point represents the mean of each group’s iMGs with at least ten cells per subject-derived iMGs (HC: *n* = 3; ALS(S)-iMGs: *n* = 6; ALS(R)-iMGs: *n* = 5). **f** The p21 mRNA expression in the three groups’ iMGs by qRT-PCR. Each dot represents data from individual-subject-derived iMGs (HC: *n* = 3; ALS(S)-iMGs: *n* = 6; ALS(R)-iMGs: *n* = 5). Values are means ± SEM. Comparisons were made against control (*P < 0.05, **P < 0.01, ***P < 0.001, ****P < 0.0001; ns, not significant; unpaired *t* test or one-way ANOVA with post hoc Tukey’s tests)

In this study, the mean ∆FS was 1.6 in ALS(R) and 0.2 in ALS(S) (Supplemental Table 1). The mean ∆FS disease duration was 13.7 months in ALS(R) and 53.0 months in ALS(S). Age at symptom onset was younger in ALS(S), but the age at the iMG sampling time was similar between the two groups. The mean difference between ALSFRS-R and ∆FS in the two groups was 10.2 (*P* < 0.001), 1.4 points/months (*P* < 0.001), suggesting that clinical status at sampling time had deteriorated more in the ALS(R) group. ALSFRS-R measured at sampling time was 27.7 in ALS(R) and 37.9 in ALS(S). We analyzed plasma cytokines from ALS patients and HCs. Plasma TNF-α and IL-8 levels were increased in both ALS groups compared with HCs (Fig. 3a**)**, consistent with a previous study [39]. In addition, Plasma IL-6 and MCP-1 levels were only increased in ALS(R). This indicated that plasma cytokines were also different between the two groups in ALS, suggesting an intrinsic role in disease progression.

For serial comparative studies, we generated iMGs from ALS(R) (R1 – R5) and ALS(S) (S1 – S6) patients. In qRT‑PCR analysis, microglia signature genes (*P2RY12*, *OLFML3*, *TGFBR1*, *GPR34*, *MERTK*, *HEXB*, *CSF1R*, and *TMEM119*) [40] were upregulated in ALS(S)-iMGs and ALS(R)-iMGs versus individual monocytes, which two-group expression level was not significantly different (Fig. 3b). Despite the lack of expression level differences in microglial signature genes between iMGs from the two ALS groups, Imaris-based morphometric analysis revealed that ALS(R)-iMGs were significantly different from ALS(S)-iMGs and HC-iMGs (Fig. 3c). Based on morphological parameters, dendritic length, number of segmentations, number of terminal points, and number of branching points were significantly reduced in both ALS group-iMGs compared to HC-iMGs, especially markedly reduced in ALS(R)-iMGs compared to ALS(S)-iMGs.

Furthermore, phagocytic function was compared between ALS(S)-iMGs and ALS(R)-iMGs. The most remarkable finding was that phagocytic function was severely impaired in ALS(R)-iMGs, whereas ALS(S)-iMGs exhibited no significant differences in phagocytic function compared to HC-iMGs (Fig. 3d, e). However, to exclude the possibility of senescence-related factors affecting to phagocytic dysfunction of microglia [41], we identified no difference in the mRNA expression of cellular senescence markers *p21*^*CIP1*^ (Fig. 3f). Thus, we ruled out aging as a factor in the phagocytic dysfunction of ALS(R)-iMGs.

### Defective Phagocytosis in ALS(R)-iMGs Is Associated with Decreased NCKAP1 Expression

**Fig. 4:**
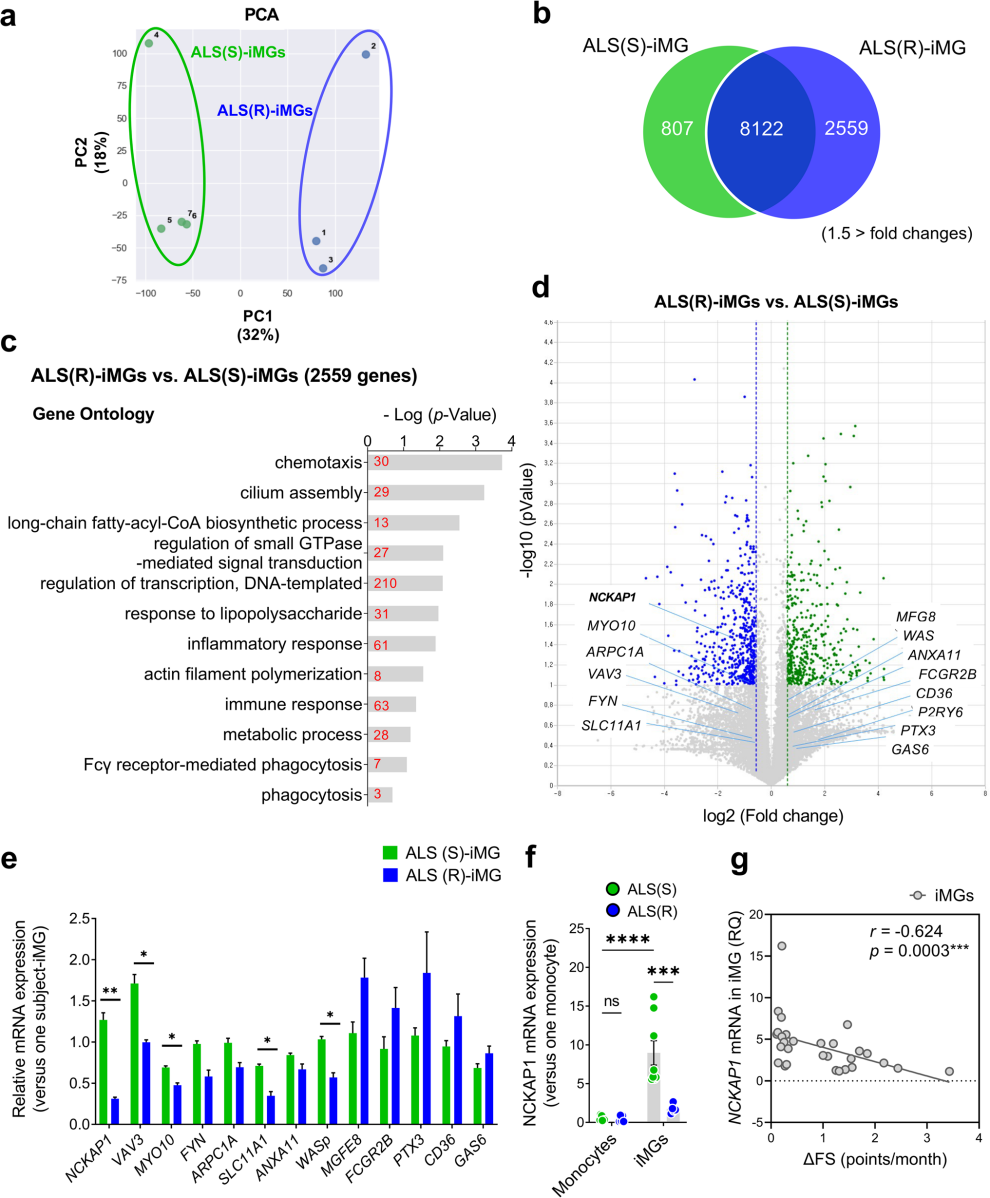
*NCKAP1* is associated with impaired phagocytic function in ALS(R)-iMGs. Transcriptome analysis of iMGs generated from three ALS(R) and four ALS(S) patients. **a** Principal component analysis (PCA) of all expressed genes in individual ALS(R)-iMGs and ALS(S)-iMGs. **b** Venn diagram illustrating that the number and overlap of transcripts differed significantly in the two groups according to RNA-seq (1.5-fold-change). **c** Functional analysis using DAVID software from 2559 transcripts that were significantly altered in ALS(R)-iMGs compared to ALS(S)-iMGs (10 GO analyses). The number within each bar indicates the number of genes in the database for the specified term. **d** Volcano plot showing transcripts with significantly altered abundance in ALS(R)-iMGs compared to ALS(S)-iMGs. We tested for significant differences between ALS(R)-iMGs and ALS(S)-iMGs samples, which are highlighted, using a cutoff of *P* values of 0.05 with a 1.5-fold-change ratio cutoff. GO genes of phagocytosis (GO: 0,006,909) are presented. **e** A subset of 13 transcripts identified as “hits” (phagocytosis-related increased or decreased abundance) in ALS(R)-iMGs compared to ALS(S)-iMGs were validated by RT-qPCR. **f** *NCKAP1* mRNA expression in individual-subject-derived monocytes or iMGs from the two groups. The symbols represent individual-subject-derived monocytes or iMGs (ALS(R): *n* = 5; ALS(S): *n* = 8, from available samples) **g** Correlation of the relative expression of *NCKAP1* in iMGs with the rate of disease progression from the symptom onset time to the sampling time point for the generation of iMGs (∆FS, points/month). Values are means ± SEM. Comparisons were made against control (*P < 0.05, **P < 0.01, ***P < 0.001, ****P < 0.0001, ns, not significant; unpaired *t* test or two-way ANOVA with post hoc Tukey’s tests)

To identify the responsible targets related to ALS(R)-iMGs phagocytic dysfunction, transcriptomic data were analyzed in another set of iMGs from ALS(R) (R6 – R8) and ALS(S) (S7 – S10) patients. In principal component analysis (PCA), ALS(R)-iMGs were distinct from ALS(S)-iMGs (Fig. 4a), and DEGs analysis (fold changes > 1.5) showed that 2559 genes were differentially expressed in ALS(R)-iMGs (Fig. 4b and Supplemental Table 6). GO analysis of 2599 genes revealed that gene subsets, including chemotaxis, cilium assembly, long-chain fatty-acyl-CoA biosynthesis, response to lipopolysaccharide, inflammatory response, actin filament polymerization, metabolic process, and phagocytosis, were differentially expressed between ALS(R)-iMGs and ALS(S)-iMGs (Fig. 4c and Supplemental Table 7).

Next, we focused on phagocytosis-related genes, revealed that genes including *NCKAP1*, *VAV3*, *MYO10*, *FYN*, *ARPCA1*, and *SLC11A1* were significantly downregulated in ALS(R)-iMGs compared to ALS(S)-iMGs (Fig. 4d). We analyzed 13 candidate transcripts related to phagocytosis in all available samples by RT-qPCR (Fig. 4e). Five genes (*NCKAP1*, *VAV3*, *MYO10, SLC11A1*, and *WASp*) reproduced the RNA-Seq results. NCK-associated protein 1 (*NCKAP1*) and guanine nucleotide exchange factor Vav 3 (*VAV3*), *MYO10*, and Wiskott-Aldrich syndrome protein (*WASp*), known intracellular signaling-regulated factors related to the actin-polymerization process in phagocytosis [42], were significantly decreased in ALS(R)-iMGs. Among them, *NCKAP1* was the candidate gene identified related to defective phagocytosis in ALS(R)-iMGs. The mRNA expression level of *NCKAP1* in individual monocytes was barely detectable, but iMGs of both groups expressed higher *NCKAP1* than the monocytes (Fig. 4f). Next, we analyzed the relationship between ALS patient progression speed and NCKAP1. *NCKAP1* mRNA levels in iMGs were negatively correlated with progression speed, such as ∆FS (*r* =  *− *0.624,* p* = 0.0003; Fig. 4g). These results suggest that *NCKAP1* gene is the most important factor related to defective phagocytosis reflecting the progression speed of ALS, and in the next step, we were to further investigate the critical role of the *NCKAP1* gene in phagocytosis.

### NCKAP1 Regulates Actin Polymerization During the Phagocytic Process in iMGs

NCKAP1 is a known member of the WAVE regulatory complex (WRC) that regulates actin filament reorganization via its interaction with the Arp2/3 complex [43]. To further delineate the role of NCKAP1 in phagocytosis, we generated another set of iMGs from ALS(R) (R9 – R15) and ALS(S) (S11 – S14) patients.

**Fig. 5:**
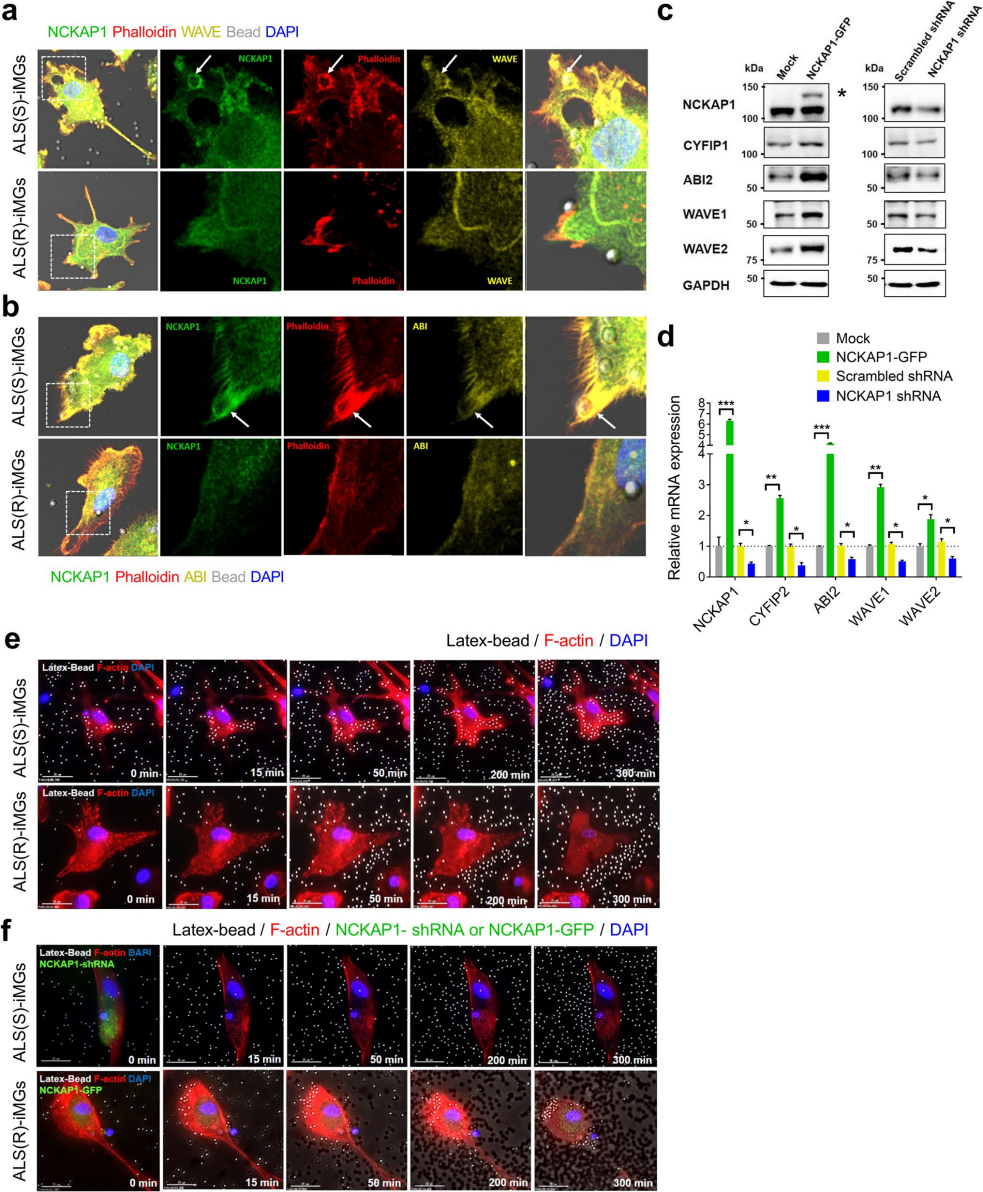
NCKAP1 regulates genes involved in actin polymerization and overexpression of NCKAP1 rescues defective phagocytic function in ALS(R)-iMGs. **a**, **b** Subcellular distribution of actin polymerization-related proteins (members of WAVE complex) and NCKAP1 during bead phagocytosis in both groups of ALS-iMGs (ALS(S)-iMGs and ALS(R)-iMGs). In ALS(S)-iMGs, WAVE (yellow; **a**) and ABI (yellow; **b**) were markedly accumulated at the phagocytic cups along with NCKAP1 (green), phalloidin (red; F-actin marker), and DAPI counterstain (blue). Arrows indicate phagocytic cups surrounding a bead. The figure is representative of independent experiments performed in replicates (*n* = 10). Scale bar: 25 μm. **c** Human NCKAP1 or shNCKAP1 was transfected into HeLa cells and changes in the expression of actin polymerization-related genes such as CYFIP1, ABI2, WAVE1, and WAVE2 were examined by western blot. An empty shRNA vector was used as a control (scramble-shRNA). * represents NCKAP1-GFP. **d** Quantification of the mRNA expression level of each normalized to GAPDH and indicated as relative expression of vehicle control (*n* = 3). Values are means ± SEM. Comparisons were made against control (*P < 0.05, **P < 0.01, ***P < 0.001; two-way ANOVA with post hoc Tukey’s tests). **e**, **f** Snapshots of live cell imaging showing phagocytosis of latex beads in both groups of ALS-iMGs. Merged images of DIC (latex bead) and fluorescence images (F-actin: red; DAPI: blue). ALS(S)-iMGs (upper) showed normal phagocytic function, whereas ALS(R)-iMGs (lower) showed a marked impairment in phagocytosis (**e**). Latex bead phagocytosis in ALS(S)-iMGs transfected with shNCKAP1-GFP (upper) or ALS(R)-iMGs transfected with NCKAP1-GFP (lower) (**f**). The impaired phagocytic function in ALS(R)-iMGs was rescued by NCKAP1 overexpression. Representative frames from a time-lapse image series (0 – 5 h) are shown. The figure is representative of independent experiments performed in replicates (*n* = 10). Scale bar: 25 μm

First of all, we investigated the relationship between NCKAP1 and other actin polymerization-related genes and the effect of NCKAP1 overexpression or knockdown on the expression of related WRC molecules such as cytoplasmic FMR1-interacting protein 1 (CYFIP1), Abelson interactor 2 (ABI2), WASP-family verprolin homologous protein 1 (WAVE1), and WAVE2. As shown in Fig. 5a and b*,* NCKAP1 (green) accumulated at the F-actin-rich apical region of the cell membrane (represented as phalloidin, red) which formed a phagocytic cup during phagocytosis in ALS(S)-iMGs (white dot-lined box and white arrow). Phagocytic cup formation is involved in actin polymerization under the plasma membrane in the initial engulfment step of phagocytosis. This was clearly observed in ALS(S)-iMGs incubated with latex beads. In addition, F-actin-rich cup-like structures co-localized with NCKAP1 (upper figures of Fig. 5a). In contrast, ALS(R)-iMGs exhibited considerably fewer phagocytic cups with accumulated F-actin at the regions of contact with latex beads (bottom of Fig. 5a). Immunofluorescence revealed that WAVE complexes, such as WAVE and ABI, co-localized with NCKAP1 in ALS(S)-iMGs but not in ALS(R)-iMGs (Fig. 5a and b).

Next, to evaluate the role of NCKAP1 in WAVE complex stability, we transfected HeLa cells with GFP-tagged NCKAP1 or GFP-tagged NCKAP1 shRNA and then performed western blots and RT-qPCR to determine the effects of NCKAP1 on actin polymerization-related proteins. We found that NCKAP1 overexpression increased the expression of actin polymerization-related proteins (CYFIP1, ABI2, WAVE1, and WAVE2), whereas NCKAP1 knockdown reduced their expression (Fig. 5c and d).

Finally, we examined whether NCKAP1 overexpression rescued the defective phagocytic function of ALS(R)-iMGs using live cell imaging. While ALS(S)-iMGs phagocytized latex beads (Fig. 5e, upper panel), ALS(R)-iMGs showed defective phagocytosis (Fig. 5e, lower panel). However, when NCKAP1 was overexpressed in ALS(R)-iMGs, phagocytosis was rescued (Fig. 5f, lower panel). As expected, the active phagocytosis in ALS(S)-iMGs was not present when NCKAP1 was knocked down (Fig. 5f, upper panel). Collectively, our data suggest that NCKAP1 plays a pivotal role in the formation of phagocytic cups by participating in the WRC complex-mediated actin polymerization process. Thus, NCKAP1 is an important potential biomarker that could be useful for predicting the state of perturbed phagocytic function of iMGs in rapidly progressing ALS patients.

### ALS(R)-iMGs Have an Exaggerated Response to Inflammatory Signaling

**Fig. 6:**
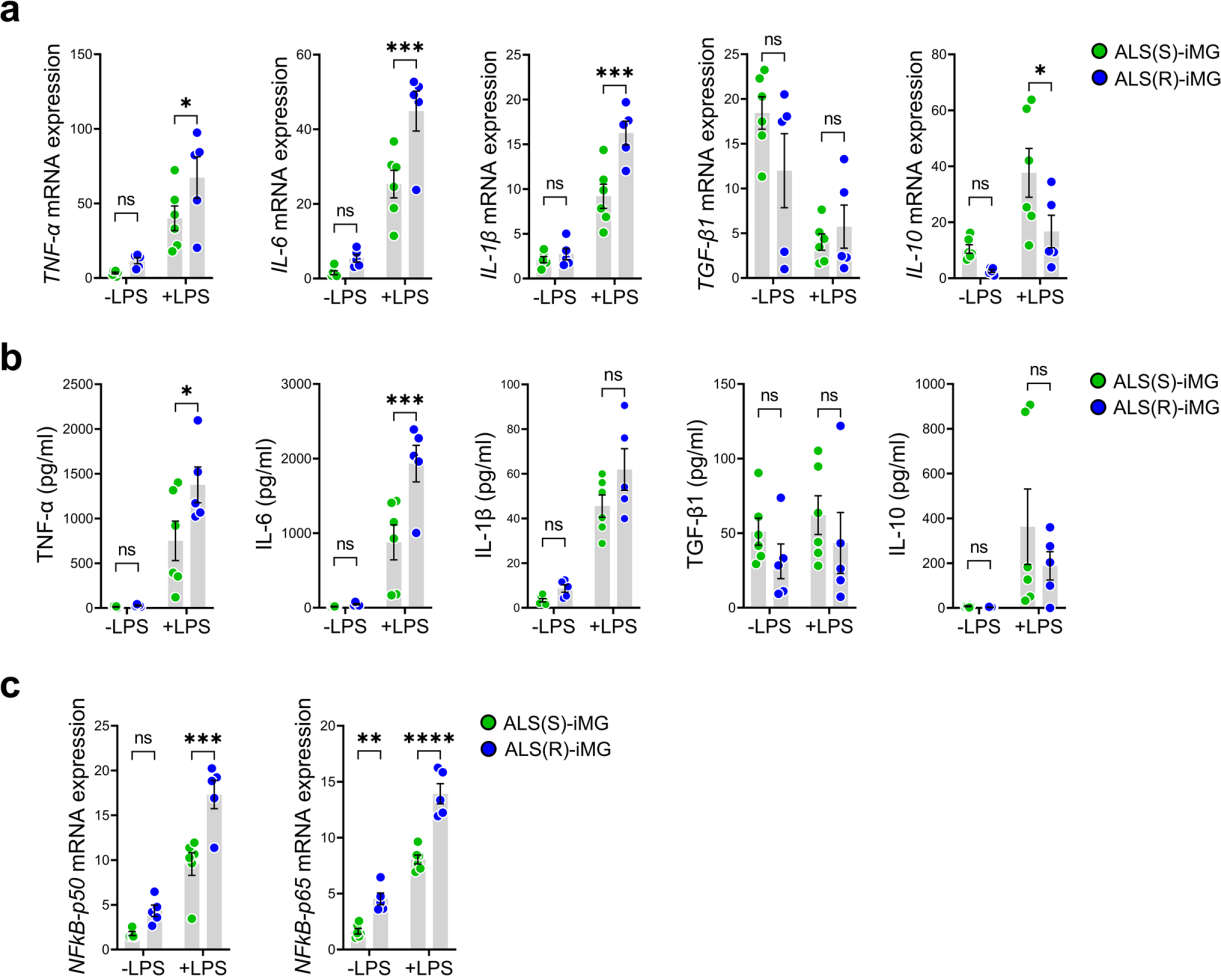
ALS(R)-iMGs exhibit an exaggerated pro-inflammatory response to LPS stimuli compared to ALS(S)-iMGs. **a**, **b** A comparison of cytokine profiles between ALS(S)-iMGs and ALS(R)-iMGs in the resting state or upon LPS stimulation (100 ng/ml), as evaluated by qRT-PCR (**a**) and ELISA (**b**). Each data point represents individual-subject-derived iMGs (ALS(S)-iMGs: *n* = 6; ALS(R)-iMGs: *n* = 5). **c** *NFκB-p50* and *NFκB-p65* mRNA expression in ALS(S)-iMGs and ALS(R)-iMGs in the resting state or upon LPS stimulation. Each data point represents individual-subject-derived iMGs (ALS(S)-iMGs: *n* = 6; ALS(R)-iMGs: *n* = 5). Values are means ± SEM. Comparisons were made against control (*P < 0.05, **P < 0.01, ***P < 0.001, ****P < 0.0001, ns, not significant; two-way ANOVA with post hoc Tukey’s tests

Phagocytosis is traditionally regarded as beneficial for tissue homeostasis; it is responsible for rapid clearance of dying cells or debris, thus preventing spillover of pro-inflammatory and neurotoxic responses [42]. Transcriptome analysis revealed that the immune response pathway, which operates in response to LPS and inflammatory signaling, functions differently in iMGs from the two ALS groups, as shown in Fig. 4c. Thus, we compared the mRNA expression levels of cytokines in response to LPS stimulation in ALS(S)-iMGs and ALS(R)-iMGs. In the unstimulated state, there was no significant difference in mRNA levels of the inflammatory cytokines (*TNF-α*, *IL-6*, *IL-1β*, *TGF-β1*, and *IL-10*) between iMGs from both ALS groups. However, LPS stimulation provoked an increase in mRNA expression of pro-inflammatory cytokines (*TNF-α*, *IL-6*, and *IL-1β*) in iMGs from both ALS groups, especially ALS(R)-iMGs exhibited an exaggerated response compared to ALS(S)-iMGs (Fig. 6a). In addition, cytokine levels in the iMGs culture media, as measured by ELISA, showed a pattern that was similar to the mRNA expression profiles (Fig. 6b). These findings suggest that the response of ALS(R)-iMGs to an inflammatory stimulus is exaggerated in comparison to the response of ALS(S)-iMGs.

To address whether the enhanced pro-inflammatory response seen in ALS(R)-iMGs is associated with decreased NCKAP1 expression, we examined the causal relationship between key inflammatory signals and NCKAP1. Because we had no frame of reference regarding the role of NCKAP1 in microglial function and inflammatory signaling, we speculated that NCKAP1 might show similar acting with NCKAP1L (NCKAP1-like). NCKAP1L and NCKAP1 belong to the same family and have similar structures. NCKAP1L is known as a crucial player in actin polymerization. It is selectively expressed in hematopoietic cells [44]. Recently, NCKAP1L was proposed to be a novel phagocytosis regulator in a phagocyte cell line [45]. Furthermore, NCKAP1L is a common upstream signal with NF-κB, which is a representative inflammatory signal in hematopoietic cells [46]. Thus, we studied whether NCKAP1 reduction is involved in NF-κB signaling in ALS(R)-iMGs. We examined NF-κB signaling in response to LPS stimulation in ALS(R)-iMGs. *NF-κB p-50* and *p-65* mRNA expression levels were upregulated in ALS(R)-iMGs upon LPS stimulation (Fig. 6c). Overall, our results indicate that NCKAP1 reduction may be related to the abnormally exaggerated inflammatory response via the NF-κB signaling pathway. This finding provides a clue as to why an enhanced pro-inflammatory response is present in ALS(R)-iMGs.

In summary, our data indicate that the perturbed phagocytic function seen in ALS(R)-iMGs is related to decreased *NCKAP1*-mediated impairment of proper actin polymerization. In post-hoc analysis indicated that ∆FS, representing the speed of progression of ALS, correlates with each patient’s *NCKAP1* mRNA level. Thus, targeting microglial *NCKAP1* may be an alternative therapeutic target in rapid sALS.

## Discussion

The significance of the current study is that we identified possible therapeutic targets associated with defective microglial function in rapidly progressing ALS using a microglia-like cell model. Using transcriptome analysis, we identified defective phagocytosis corresponding with reduced *NCKAP1* levels as the key factor that gave rise to phagocytic dysfunction in ALS(R)-iMGs. Though there was no significant difference in major homeostatic gene profiles between the two groups of ALS-iMGs, as shown in Fig. 3b, *NCKAP1* levels were reduced in ALS(R)-iMGs as compared with ALS(S)-iMGs. Only ALS(R)-iMGs showed intrinsically perturbed phagocytosis and an exaggerated inflammatory response in response to LPS stimulus. These data imply that *NCKAP1* reduction in ALS(R)-iMGs may be responsible for both defective phagocytosis and the accelerated inflammatory response. This result is supported by data on ALS(S)-iMGs showing intact phagocytic function with less of a pro-inflammatory response than is present in ALS(R)-iMGs.

The expression of NCKAP1 is widespread throughout the body and plays a role in various cellular processes, including cytoskeletal organization, cell migration, and signal transduction. In the brain, NCKAP1 is predominantly expressed in the cerebellum, cerebral cortex, and hippocampus, where it plays a role in synaptic plasticity and memory formation [47]. However, only a few studies have reported an association between NCKAP1 and neurodegenerative diseases; for example, *NCKAP1* gene expression is known to be reduced in AD [48]. Most studies of NCKAP1 have focused on its role in neuronal differentiation and axonal growth as a cytoskeletal regulator during the development [47, 49, 50]. Furthermore, *NCKAP1L*, a hematopoietic cell-specific gene that has a similar structure to *NCKAP1*, has been reported as a key player in actin polymerization. It is also essential for neutrophil and macrophage migration and phagocytosis [44, 45, 51]. NCKAP1L family members are known to regulate actin polymerization, morphogenesis, and immunity [44]. Both NCKAP1 and NCKAP1L are members of the WRC that consists of Abi (Abelson interactor 1 or 2), WAVE (WAVE 1, 2, 3), Sra1 (specifically Rac-associated protein 1), and activated Arp2/3 (actin-related protein-2/3), all of which can promote actin polymerization. In contrast to NCKAP1L, NCKAP1 is enriched in the brain, but absent or less expressed in hematopoietic cells [46]. Our results showed that ALS(R)-iMGs overexpressing NCKAP1 exhibited restored phagocytic function and increased expression levels of CYFIP1, ABI2, WAVE1, and WAVE2. Because the stability of WRC is interdependent on the presence of individual WRC components [44], low NCKAP1 expression may cause instability of the complex and hinder F-actin polymerization in microglia-like cells. Therefore, NCKAP1 is presumed to play a key role in the engulfment process of phagocytosis by regulating actin cytoskeleton dynamics. We demonstrated that NCKAP1 overexpression could restore the phagocytic capacity in this in vitro study. However, further study should be required to determine whether microglial NCKAP1 overexpression could truly slow the disease progression in ALS animal studies.

Numerous studies have focused on toxic microglia as a factor in ALS progression. Reactive microglia can aggravate motor neuron death through pro-inflammatory cytokine secretion in *SOD1* mice. Depletion of defective microglial cells can resolve neuroinflammation and result in prolonged survival [52]. Additionally, ALS(R)-iMGs showed an intense pro-inflammatory reaction to LPS stimuli, in accordance with the results of previous *SOD1* mouse and human studies [10, 53, 54]. This reaction is distinct from the general characteristics of aged microglia [38, 55]. The exaggerated pro-inflammatory response of ALS(R)-iMGs to LPS stimuli (like immune vigilance) [41] may be associated with reduced expression of *NCKAP1* and related WRC genes. In the hematopoietic system, cytokine expression via NF-κB signaling and actin polymerization for phagocytosis have a common upstream signaling molecule, Rac small GTPase. However, these two pathways bifurcate upstream of Rac [44]. Thus, Rac activation by LPS can induce both NF-κB signaling and phagocytosis. However, reduced expression of NCKAP1 and the resulting decrease in actin polymerization-related proteins may shift Rac-GTP signaling toward NF-κB signaling, causing NF-κB over-activation and increased levels of pro-inflammatory cytokines in microglia. Our results support this speculation. ALS(R)-iMGs exhibited lower *NCKAP1* expression but higher NF-κB expression upon LPS stimulation than did ALS(S)-iMGs. Similar findings were observed in neurodegeneration-associated molecular patterns (NAMPs) [56], including mutant *SOD1*, *FUS*, *TDP-43*, RNA foci and RAN dipeptides, and degenerating neuronal debris. NAMPs may trigger a chronic inflammatory milieu in CNS, while microglia acting under acute inflammatory conditions in response to LPS have distinct activation profiles [57]. Regulatory T cells are reliable factor reflecting ALS progression and suppress inflammatory microglia function [58, 59]. Although we did not examine regulatory T cells in ALS patients due to limited amounts of obtained PBMCs, the further study will be useful to compare the function of regulatory T cells and ALS-iMGs in ALS progression.

Our study leaves several unanswered questions. There is still an argument that the iMGs are closer to infiltrated blood-derived macrophages than resident microglia [21]. iMGs are not absolutely identical to resident brain microglia. They cannot precisely reflect the characteristics of yolk sac-originated resident microglia in the non-diseased brain [60]. To overcome these inevitable hurdles of iMGs, further studies should be needed to develop new, detailed markers that can discriminate iMGs from or correlate iMGs with microglia subpopulations and diverse macrophage populations [9]. More precise single-cell assay-based analytic approaches to the study of iMGs are needed.

We did not clarify how *NCKAP1* was reduced in only ALS(R)-iMGs in our culture environment. One possible mechanism is through maintenance of epigenetic memory during direct conversion [61]. Direct conversion methods, including ours, have an advantage in that they preserve the aging-associated features of the donors, while iPSC models alter the epigenetic landscape during rejuvenation [62]. Further studies are needed to clarify whether our model maintains epigenetic memory. Another limitation of this study is related to the isolation of brain microglia from ALS patients using CD11b-positive beads that was done in order to compare the brain microglia with our iMGs model. These cells might be a mixture of infiltrated monocytes/macrophages and resident microglia. In addition, we cannot exclude the possibility that the inflammatory characteristics of monocytes in rapidly progressing ALS patients [39] might contribute to the characteristics of our iMGs, despite the fact that *NCKAP1* expression in the monocytes of ALS patients is rare. This study was aimed at the descriptive difference between induced microglia originating from PBMC of slow-progressed ALS patients and rapid-progressed ALS patients. Our data mainly focused on the difference between two extreme groups of ALS at this time. We only demonstrated only limited small number of healthy control, and the number of both patients was not sufficient. This study would be the preliminary discovery level of a cohort study for the development of biomarkers, which should be more recapitulated and validated in a larger cohort.

## Conclusions

Despite of the abovementioned limitation, we report for the first time that defective phagocytic function in microglia-like cells of rapidly progressing sALS patients is involved in the reduced expression of *NCKAP1*. NCKAP1 reduction in microglia may interfere with the engulfment step of phagocytosis and induce immune vigilance, leading to rapid progression in ALS. In addition, our discovery cohort showed that an increase in the levels of *NCKAP1* expression in iMGs was closely correlated with the clinical speed of progression in sALS patient. Therefore, *NCKAP1*-mediated disruptions in phagocytosis may be a therapeutic target in slowing ALS progression.

## Supplementary Information

Below is the link to the electronic supplementary material.

Supplementary file1 (DOC 1990 KB)

Supplementary file2 (XLSX 582 KB)

Supplementary file3. Live image of phagocytosis of latex bead in ALS(S)-iMGs (AVI 2022 KB)

Supplementary file4. Live image of phagocytosis in ALS(R)-iMGs (AVI 2409 KB)

Supplementary file5. Live image of phagocytosis in ALS(S)-iMGs after NCKAP1 knockdown (AVI 5147 KB)

Supplementary file6. Live image of phagocytosis in ALS(R)-iMGs after NCKAP1 overexpression (AVI 8339 KB)

## Data Availability

All data generated or analyzed during this study are included in the source data file and supplementary data.

## Glossary

ALS(S): Slowly progressive ALS
ALS(R): Rapidly progressive ALS
ALSFRS-R: Amyotrophic Lateral Sclerosis Functional Rating Scale–Revised
DEGs: Differentially expressed genes
ELISA: Enzyme-linked immunosorbent assay
HC: Healthy control
iMG: Microglia-like cell induced by monocyte
NCKAP1: NCK-associated protein 1
NCKAP1L: NCK-associated protein 1 like
WASP: Wiskott-Aldrich syndrome protein
WAVE: WASP-family verprolin homologous protein
WRC: WAVE regulatory complex
