# Supplementary material for: Role of NCKAP1 in the Defective Phagocytic Function of Microglia-Like Cells Derived from Rapidly Progressing Sporadic ALS
Source: Mol Neurobiol. 2023 May 8;60(8):4761–77. doi: 10.1007/s12035-023-03339-2 (PMC10293423; doi:10.1007/s12035-023-03339-2)
Supplement: Supplementary file 1 — Supplementary file1 (DOC 1990 KB) [file 12035_2023_3339_MOESM1_ESM.doc]

**Supplementary Material**

**Role of NCKAP1 in the defective phagocytic function of microglia-like cells derived from rapidly progressing sporadic ALS**

Supplemental Table 1. Demographics of the enrolled participants.

Supplemental Table 2. List of gene panels related to ALS, FTD, and other types of dementia.

Supplemental Table 3. Antibodies and primers.

Supplemental Table 4. List of 705 shared genes in iMGs and Brain-MG according to whole-transcriptome differential gene expression (3-fold-change).

Supplemental Table 5. GO analysis and KEGG pathways in 705 genes shared by iMGs and brain-MG.

Supplemental Table 6. List of 2,559 shared genes in ALS(R)-iMGs compared to ALS(S)-iMGs according to whole-transcriptome differential gene expression (1.5-fold-change).

Supplemental Table 7. GO analysis of 2,559 genes significantly altered in ALS(R)-iMGs compared to ALS(S)-iMGs.

**Supplemental Table 1. Demographics of the enrolled participants.**

| **Participant** | **MND no.** | **Revised El Escorial criteria of ALS** | **Sex** | **Symptom onset (T_0_)** | **Baseline visit (T_1_)** | | **Sampling time for iMGs (T_2_)** | | **Disease duration (T_0_-T_2_) ^a^, months** | **∆FS (T_0_-T_2_) ^b^, point/months** | **Site of onset** | **Family history of ALS** |
| --- | --- | --- | --- | --- | --- | --- | --- | --- | --- | --- | --- | --- |
|  |  |  |  | **Age, year** | **Age, year** | **ALSFRS-R** | **Age, year** | **ALSFRS-R** |  |  |  |  |
| ALS (R)-1 | 1542 | CD | M | 56.1 | 56.5 | 42 | 56.7 | 24 | 7 | 3.43 | Upper limb | No |
| ALS (R)-2 | 1426 | CP | M | 47.2 | 47.8 | 37 | 48.7 | 9 | 16 | 2.44 | Combined | No |
| ALS (R)-3 | 1582 | CP | M | 55.3 | 56.3 | 29 | 56.4 | 28 | 13 | 1.54 | Combined | No |
| ALS (R)-4 | 1518 | CP | F | 47.6 | 48.0 | 39 | 48.4 | 34 | 9 | 1.56 | Lower limb | No |
| ALS (R)-5 | 1745 | CD | M | 68.1 | 68.3 | 34 | 69.7 | 20 | 19 | 1.47 | Bulbar | No |
| ALS (R)-6 | 1862 | CP | F | 56.5 | 56.6 | 38 | 57.1 | 38 | 7 | 1.43 | Upper limb | No |
| ALS (R)-7 | 1875 | CD | M | 61.6 | 61.8 | 33 | 62.7 | 33 | 12 | 1.25 | Lower limb | No |
| ALS (R)-8 | 1914 | CD | M | 64.4 | 65.5 | 31 | 65.5 | 31 | 13 | 1.31 | Bulbar | No |
| ALS (R)-9 | 1667 | CP | F | 55.3 | 56.3 | 37 | 57.3 | 23 | 23 | 1.09 | Lower limb | No |
| ALS (R)-10 | 1596 | CP | F | 50.6 | 51.6 | 38 | 52.5 | 26 | 22 | 1.00 | Bulbar | No |
| ALS (R)-11 | 1917 | CP | M | 59.2 | 59.9 | 39 | 60.0 | 36 | 10 | 1.20 | Bulbar | No |
| ALS (R)-12 | 1901 | CP | M | 49.3 | 49.8 | 39 | 50.5 | 24 | 13 | 1.85 | Upper limb | No |
| ALS (R)-13 | 1956 | CP | F | 55.6 | 55.9 | 40 | 56.2 | 35 | 6 | 2.17 | Axial muscle | No |
| ALS (R)-14 | 1696 | CP | M | 54.0 | 55.0 | 32 | 56.2 | 23 | 26 | 1.00 | Lower limb | No |
| ALS (R)-15 | 1955 | CPL | F | 59.7 | 60.2 | 40 | 60.5 | 31 | 10 | 1.70 | Lower limb | No |
| ALS (S)-1 | 1401 | CPL | F | 57.0 | 59.5 | 45 | 60.2 | 44 | 38 | 0.11 | Upper limb | No |
| ALS (S)-2 | 1537 | CP | M | 55.9 | 61.5 | 37 | 62.8 | 37 | 83 | 0.13 | Lower limb | No |
| ALS (S)-3 | 1406 | CP | F | 63.1 | 66.4 | 41 | 67.1 | 38 | 48 | 0.21 | Upper limb | No |
| ALS (S)-4 | 1538 | CPL | M | 56.8 | 57.6 | 44 | 58.9 | 41 | 24 | 0.29 | Axial muscle | No |
| ALS (S)-5 | 1476 | CPL | M | 47.8 | 48.3 | 46 | 49.0 | 42 | 14 | 0.43 | Upper limb | No |
| ALS (S)-6 | 1530 | CP | F | 46.2 | 47.4 | 39 | 48.8 | 38 | 30 | 0.33 | Upper limb | No |
| ALS (S)-7 | 1757 | CP | M | 46.4 | 47.0 | 42 | 48.6 | 41 | 26 | 0.27 | Lower limb | No |
| ALS (S)-8 | 328 | CP | M | 50.8 | 59.5 | 34 | 68.4 | 18 | 210 | 0.14 | Lower limb | No |
| ALS (S)-9 | 1664 | CP | F | 36.0 | 38.0 | 45 | 39.1 | 43 | 37 | 0.14 | Upper limb | No |
| ALS (S)-10 | 210 | CD | M | 46.6 | 54.5 | 45 | 57.6 | 20 | 132 | 0.21 | Lower limb | No |
| ALS (S)-11 | 1929 | CPL | F | 47.8 | 49.3 | 40 | 50.5 | 40 | 32 | 0.25 | Axial muscle | No |
| ALS (S)-12 | 1713 | P | M | 54.0 | 54.7 | 44 | 56.1 | 43 | 25 | 0.20 | Lower limb | No |
| ALS (S)-13 | 1950 | CP | F | 49.2 | 49.5 | 46 | 50.6 | 45 | 16 | 0.19 | Upper limb | No |
| ALS (S)-14 | 1697 | P | M | 50.8 | 51.8 | 45 | 53.1 | 40 | 27 | 0.30 | Lower limb | No |
| HC-1 | N/A | Control | M | N/A | N/A | N/A | 36.9 | N/A | N/A | N/A | N/A | N/A |
| HC-2 | N/A | Control | M | N/A | N/A | N/A | 33.8 | N/A | N/A | N/A | N/A | N/A |
| HC-3 | N/A | Control | M | N/A | N/A | N/A | 27.7 | N/A | N/A | N/A | N/A | N/A |
| HC-4 | N/A | Control | F | N/A | N/A | N/A | 37.7 | N/A | N/A | N/A | N/A | N/A |
| HC-5 | N/A | Control | F | N/A | N/A | N/A | 27.8 | N/A | N/A | N/A | N/A | N/A |

In out cohort, symptom onset time was defined as T_0_, the baseline point at the initial visit time for diagnosis or treatment as T_1_, and the sampling timepoint for the generation of iMGs as T_2_.

^a^Disease duration: the period from symptom onset time (T_0_) to the sampling time point for the generation of iMGs (T_2_).

^b^The progression rate was defined as ∆FS (T_0_-T_2_), (i.e., (48 - ALSFRS-R score at T_2_)/ (duration from T_0_ to T_2_, months)).

ALS patients were categorized as follows: rapidly progressive (∆FS (T_0_-T_2_) ≥ 1.0) and slowly progressive (∆FS (T_0_-T_2_) < 0.5).

Abbreviations: ALSFRS-R = amyotrophic lateral sclerosis functional rating scale-revised (48 [normal] to 0 [maximally impaired]), CD = clinically definite, CP = clinically probable, CPL = clinically probable with laboratory-supported diagnosis, P = possible, n/c = not checked, N/A = not applicable.

**Supplemental Table 2. List of gene panels related to ALS, FTD, and other types of dementia**

| **Gene symbol** | **RefSeq** | **Gene description** | **Chromosomal**  **location** | **Inheritance** |
| --- | --- | --- | --- | --- |
| **ALS- and FTD-related genes (27 genes)** | | | | |
| *ALS2* | NM_020919.3 | Amyotrophic lateral sclerosis 2 | 2q33.1 | AD |
| *ANG* | NM_001145.4 | Angiogenin, ribonuclease, RNase A family, 5 | 14q11.1-q11.2 | AD |
| *CHMP2B* | NM_014043.3 | Chromatin-modifying protein 2B | 3p11.2 | AD |
| *CHRNA4* | NM_000744.6 | Acetylcholine receptor, neuronal nicotinic, alpha-4 subunit | 20q13.2-q13.3 | AD |
| *DAO* | NM_001917.4 | D-amino-acid oxidase | 12q24 | AD |
| *DCTN1* | NM_004082.4 | Dynactin 1 | 2p13 | AD |
| *FIG4* | NM_014845.5 | FIG4 phosphoinositide 5-phosphatase | 6q21 | AR |
| *FUS* | NM_004960.3 | FUS RNA-binding protein | 16p11.2 | AD |
| *GRN* | NM_002087.2 | Granulin | 17q21.32 | AD |
| *HNRNPA1* | NM_031157.2 | Heterogeneous nuclear ribonucleoprotein A1 | 12q13.1 | AD |
| *HNRNPA2B1* | NM_031243.2 | Heterogeneous nuclear ribonucleoprotein A2/B1 | 7p15 | AD |
| *MAPT* | NM_005910.5 | Microtubule-associated protein tau | 17q21.1 | AD |
| *MATR3* | NM_199189.2 | Matrin3 | 5q31.2 | AD |
| *OPTN* | NM_021980.4 | Optineurin | 10p13 | AD |
| *PRNP* | NM_000311.3 | Prion protein | 20p13 | AD |
| *SETX* | NM_015046.5 | Senataxin | 9q34.13 | AD/AR |
| *SIGMAR1* | NM_005866.2 | Sigma non-opioid intracellular receptor 1 | 9p13.3 | AD/AR |
| *SOD1* | NM_000454.4 | Superoxide dismutase 1 | 21q22.11 | AD |
| *SPG11* | NM_025137.3 | SPG11, spatacsin vesicle trafficking associated | 15q14 | AR |
| *SQSTM1* | NM_003900.4 | Sequestosome 1 | 5q35 | AD |
| *TAF15* | NM_139215.2 | TATA-box binding protein-associated factor 15 | 17q11.1-q11.2 | AD |
| *TARDBP* | NM_007375.3 | TAR DNA-binding protein | 1p36.22 | AD |
| *TBK1* | NM_013254.3 | Tank-binding kinase 1 | 12q14.2 | AD |
| *TREM2* | NM_018965.2 | Triggering receptor expressed on myeloid cells 2 | 6p21.1 | AR |
| *UBQLN2* | NM_013444.3 | Ubiquilin 2 | Xp11.21 | XL |
| *VAPB* | NM_004738.4 | VAMP (vesicle-associated membrane protein)-associated protein B and C | 20q13.33 | AD |
| *VCP* | NM_007126.3 | Valosin-containing protein | 9p13.3 | AD |
| **Other dementia-related genes (19 genes)** | | | | |
| *AARS2* | NM_020745.3 | Alanyl-tRNA synthetase 2, mitochondrial | 6p21.1 | AR |
| *ABCD1* | NM_000033.3 | ATP-binding cassette subfamily D member 1 | Xq28 | XL |
| *APP* | NM_000484.3 | Amyloid-beta precursor protein | 21q21.2 | AD |
| *ARSA* | NM_000487.5 | Arylsulfatase A | 22q13.33 | AR |
| *CSF1R* | NM_005211.3 | Colony-stimulating factor 1 receptor | 5q32 | AD |
| *DARS2* | NM_018122.4 | Aspartyl-tRNA synthetase 2, mitochondrial | 1q25.1 | AR |
| *EIF2B1* | NM_001414.3 | Eukaryotic translation initiation factor 2B subunit alpha | 12q24.31 | AR |
| *EIF2B2* | NM_014239.3 | Eukaryotic translation initiation factor 2B subunit beta | 14q24.3 | AR |
| *EIF2B3* | NM_020365.4 | Eukaryotic translation initiation factor 2B subunit gamma | 1p34.1 | AR |
| *EIF2B4* | NM_015636.3 | Eukaryotic translation initiation factor 2B subunit delta | 2p23.3 | AR |
| *EIF2B5* | NM_003907.2 | Eukaryotic translation initiation factor 2B subunit epsilon | 3q27.1 | AR |
| *GALC* | NM_000153.3 | Galactosylceramidase | 14q31.3 | AR |
| *GBA* | NM_001005741.2 | Glucosidase, beta acid | 1q21 | AD, susceptibility |
| *GLA* | NM_000169.2 | Galactosidase alpha | Xq22.1 | XL |
| *NOTCH3* | NM_000435.2 | Notch 3 | 19q13.12 | AD |
| *PSEN1* | NM_000021.3 | Presenilin-1 (Alzheimer’s disease 3) | 14q24.3 | AD |
| *PSEN2* | NM_000447.2 | Presenilin-2 (Alzheimer’s disease 4) | 1q31-q42 | AD |
| *SNCB* | NM_001001502.1 | Synuclein, beta | 5q35 | AD |
| *TYROBP* | NM_003332.3 | TYRO protein tyrosine kinase-binding protein | 19q13.12 | AR |

Abbreviations: AD = autosomal dominant, AR = autosomal recessive, FTD = Frontotemporal dementia, RefSeq = reference sequence, XL = X-linked

**Supplemental Table 3. Antibodies and primers.**

| **a) Antibody** | **Application** | **Dilution** | **Manufacturer** | **Order No**. |
| --- | --- | --- | --- | --- |
| rabbit anti-P2RY12 | ICC | 1:200 | R&D Systems | ab140862 |
| rabbit anti-P2RY12 | ICC | 1:200 | Novus Biologicals | NBP2-33870 |
| mouse anti-IBA1 | ICC | 1:200 | R&D Systems | ab15690 |
| rabbit anti-IBA1 | ICC | 1:200 | Wako | 019-19741 |
| rabbit anti-NCKAP1 | ICC/IB | 1:200/1:1000 | Novus Biologicals | NBP1-83269 |
| mouse anti-WAVE | ICC | 1:200 | Santa Cruz Biotech | sc-373889 |
| mouse anti-ABI | ICC/IB | 1:200/1:1000 | Santa Cruz Biotech | sc-398554 |
| rabbit anti-CYFIP1 | IB | 1:1000 | Sigma-Aldrich | SAB2700152 |
| mouse anti-WAVE2 | IB | 1:1000 | Santa Cruz Biotech | sc-373889 |
| rabbit anti-WAVE1 | IB | 1:1000 | Sigma-Aldrich | SAB4503508 |
| mouse anti-GAPDH | IB | 1:1000 | Santa Cruz Biotech | sc-25778 |
| Alexa 488-conjugated antibodies | ICC | 1:500 | Invitrogen | A11001, A11005 |
| Alexa 594-conjugated antibodies | ICC | 1:500 | Invitrogen | A11012, A11005 |
| Alexa 647-conjugated antibodies | ICC | 1:500 | Invitrogen | A21235, A21244 |
| Anti-mouse IgG HRP | IB | 1:1000 | Santa Cruz Biotech | sc-516102 |
| Anti-Rabbit IgG HRP | IB | 1:1000 | Santa Cruz Biotech | sc-2357 |
| **b) RT-qPCR Primer** | **Application** | | **Manufacturer** | **Order No**. |
| P2RY12 | RT-qPCR | | Qiagen | PPH02545B |
| OLFML3 | RT-qPCR | | Qiagen | PPH07681A |
| TGFBR1 | RT-qPCR | | Qiagen | PPH00237C |
| TMEM119 | RT-qPCR | | Qiagen | PPH21875A |
| TREM2 | RT-qPCR | | Qiagen | PPH06065E |
| GAPDH | RT-qPCR | | Qiagen | PPH00150F |
| GPR34 | RT-qPCR | | Qiagen | PPH08814A |
| MERTK | RT-qPCR | | Qiagen | PPH 16600A |
| CSF1R | RT-qPCR | | Qiagen | PPH00191F |
| HEXB | RT-qPCR | | Qiagen | PPH09801A |
| p21 (CDKN1A) | RT-qPCR | | Qiagen | PPH00211E |
| p16 (CDKN2A) | RT-qPCR | | Qiagen | PPH00207C |
| NCKAP1 | RT-qPCR | | Qiagen | PPH15666A |
| VAV3 | RT-qPCR | | Qiagen | PPH0150E |
| MYO10 | RT-qPCR | | Qiagen | PPH09689A |
| FYN | RT-qPCR | | Qiagen | PPH15624A |
| ARPC1A | RT-qPCR | | Qiagen | PPH16239A |
| SLC11A1 | RT-qPCR | | Qiagen | PPH05732F |
| MFGE8 | RT-qPCR | | Qiagen | PPH07218A |
| ANXA11 | RT-qPCR | | Qiagen | PPH06949A |
| WAS | RT-qPCR | | Qiagen | PPH07123A |
| PTX3 | RT-qPCR | | Qiagen | PPH01105A |
| CD36 | RT-qPCR | | Qiagen | PPH01456A |
| FCGR2B | RT-qPCR | | Qiagen | PPH02368C |
| GAS6 | RT-qPCR | | Qiagen | PPH00025F |

Abbreviations: IB = immunoblot, ICC = Immunocytochemistry, RT-qPCR **=** quantitative real-time PCR

**Supplemental Table 4. List of 705 shared genes in iMGs and Brain-MG according to whole-transcriptome differential gene expression (3-fold-change).**

| **Gene symbol** | **Transcript_id** | **Description** | **Gene symbol** | **Transcript_id** | **Description** | **Gene symbol** | **Transcript_id** | **Description** |
| --- | --- | --- | --- | --- | --- | --- | --- | --- |
| HES4 | NM_021170 | hes family bHLH transcription factor 4 | SNX33 | NM_153271 | sorting nexin 33 | PRR34 | NM_018280 | proline rich 34 |
| C1orf233 | NM_001242659 | chromosome 1 open reading frame 233 | CSPG4 | NM_001897 | chondroitin sulfate proteoglycan 4 | HDAC10 | NM_032019 | histone deacetylase 10 |
| LOC115110 | NR_037844 | uncharacterized LOC115110 | FBXO22-AS1 | NR_003136 | FBXO22 antisense RNA 1 | MAPK11 | NR_110887 | mitogen-activated protein kinase 11 |
| TNFRSF9 | NM_001561 | tumor necrosis factor receptor superfamily member 9 | STARD5 | NM_181900 | StAR related lipid transfer domain containing 5 | RPL23AP82 | NR_026981 | ribosomal protein L23a pseudogene 82 |
| ERRFI1 | NM_018948 | ERBB receptor feedback inhibitor 1 | NMB | NM_205858 | neuromedin B | SRGAP3 | NM_014850 | SLIT-ROBO Rho GTPase activating protein 3 |
| SLC2A5 | NM_003039 | solute carrier family 2 member 5 | PEX11A | NM_001271572 | peroxisomal biogenesis factor 11 alpha | CIDECP | NR_002786 | cell death-inducing DFFA-like effector c pseudogene |
| SPSB1 | NM_025106 | splA/ryanodine receptor domain and SOCS box containing 1 | TTC23 | NR_109947 | tetratricopeptide repeat domain 23 | HRH1 | NM_001098213 | histamine receptor H1 |
| APITD1-CORT | NM_001270517 | APITD1-CORT readthrough | LRRC28 | NM_144598 | leucine rich repeat containing 28 | PPARG | NM_005037 | peroxisome proliferator activated receptor gamma |
| PDPN | NM_198389 | podoplanin | DECR2 | NM_020664 | 2,4-dienoyl-CoA reductase 2, peroxisomal | FGD5 | NM_152536 | FYVE, RhoGEF and PH domain containing 5 |
| TMEM51-AS1 | NR_027136 | TMEM51 antisense RNA 1 | PIGQ | NM_004204 | phosphatidylinositol glycan anchor biosynthesis class Q | XIRP1 | NM_001198621 | xin actin binding repeat containing 1 |
| TMEM51 | NM_001136216 | transmembrane protein 51 | METRN | NM_024042 | meteorin, glial cell differentiation regulator | CDCP1 | NM_022842 | CUB domain containing protein 1 |
| EPHA2 | NM_004431 | EPH receptor A2 | HAGHL | NM_001290139 | hydroxyacylglutathione hydrolase-like | LOC102724297 | NR_125406 | uncharacterized LOC102724297 |
| IGSF21 | NM_032880 | immunoglobin superfamily member 21 | TELO2 | NM_016111 | telomere maintenance 2 | PRKAR2A | NM_004157 | protein kinase cAMP-dependent type II regulatory subunit alpha |
| NBL1 | NM_001278164 | neuroblastoma 1, DAN family BMP antagonist | IFT140 | NM_014714 | intraflagellar transport 140 | LAMB2 | NM_002292 | laminin subunit beta 2 |
| LDLRAD2 | NM_001013693 | low density lipoprotein receptor class A domain containing 2 | TNFRSF12A | NM_016639 | tumor necrosis factor receptor superfamily member 12A | MST1 | NM_020998 | macrophage stimulating 1 |
| HSPG2 | NM_005529 | heparan sulfate proteoglycan 2 | ZNF205 | NM_003456 | zinc finger protein 205 | HYAL2 | NM_033158 | hyaluronoglucosaminidase 2 |
| C1QC | NM_172369 | complement component 1, q subcomponent, C chain | CORO7-PAM16 | NM_001201479 | CORO7-PAM16 readthrough | DOCK3 | NM_004947 | dedicator of cytokinesis 3 |
| C1QB | NM_000491 | complement component 1, q subcomponent, B chain | PAM16 | NM_016069 | presequence translocase-associated motor 16 homolog (S. cerevisiae) | ACY1 | NM_001198895 | aminoacylase 1 |
| ZNF436 | NM_030634 | zinc finger protein 436 | EMP2 | NM_001424 | epithelial membrane protein 2 | IL17RB | NM_018725 | interleukin 17 receptor B |
| ZNF436-AS1 | NR_033690 | ZNF436 antisense RNA 1 | MKL2 | NM_014048 | MKL1/myocardin like 2 | C3orf14 | NM_001291943 | chromosome 3 open reading frame 14 |
| GALE | NM_000403 | UDP-galactose-4-epimerase | SYT17 | NM_016524 | synaptotagmin 17 | FAM86DP | NR_024241 | family with sequence similarity 86 member D, pseudogene |
| GPN2 | NM_018066 | GPN-loop GTPase 2 | IQCK | NR_130968 | IQ motif containing K | FILIP1L | NM_001282794 | filamin A interacting protein 1-like |
| TMEM222 | NR_037577 | transmembrane protein 222 | GPRC5B | NM_001304771 | G protein-coupled receptor class C group 5 member B | TMEM45A | NM_018004 | transmembrane protein 45A |
| SNORA61 | NR_002987 | small nucleolar RNA, H/ACA box 61 | ACSM3 | NM_202000 | acyl-CoA synthetase medium-chain family member 3 | CCDC80 | NM_199512 | coiled-coil domain containing 80 |
| RAB42 | NM_152304 | RAB42, member RAS oncogene family | EARS2 | NR_003501 | glutamyl-tRNA synthetase 2, mitochondrial | CHCHD6 | NM_032343 | coiled-coil-helix-coiled-coil-helix domain containing 6 |
| MECR | NM_016011 | mitochondrial trans-2-enoyl-CoA reductase | KDM8 | NM_024773 | lysine demethylase 8 | EEFSEC | NM_021937 | eukaryotic elongation factor, selenocysteine-tRNA-specific |
| SDC3 | NM_014654 | syndecan 3 | IL21R-AS1 | NR_037158 | IL21R antisense RNA 1 | FAIM | NM_001033030 | Fas apoptotic inhibitory molecule |
| FABP3 | NM_004102 | fatty acid binding protein 3 | NUPR1 | NM_001042483 | nuclear protein 1, transcriptional regulator | RBP1 | NM_002899 | retinol binding protein 1 |
| MTMR9LP | NR_026850 | myotubularin related protein 9-like, pseudogene | SEZ6L2 | NM_012410 | seizure related 6 homolog like 2 | TM4SF1 | NM_014220 | transmembrane 4 L six family member 1 |
| ZBTB8A | NM_001291496 | zinc finger and BTB domain containing 8A | ASPHD1 | NM_181718 | aspartate beta-hydroxylase domain containing 1 | WWTR1 | NM_001168280 | WW domain containing transcription regulator 1 |
| SH3D21 | NM_001162530 | SH3 domain containing 21 | HSD3B7 | NM_025193 | hydroxy-delta-5-steroid dehydrogenase, 3 beta- and steroid delta-isomerase 7 | B3GALNT1 | NM_003781 | beta-1,3-N-acetylgalactosaminyltransferase 1 (globoside blood group) |
| MANEAL | NM_001031740 | mannosidase endo-alpha like | MMP2 | NM_001302508 | matrix metallopeptidase 2 | LAMP3 | NM_014398 | lysosomal associated membrane protein 3 |
| COL9A2 | NM_001852 | collagen type IX alpha 2 | MT1L | NR_001447 | metallothionein 1L (gene/pseudogene) | LINC00888 | NR_038302 | long intergenic non-protein coding RNA 888 |
| RIMS3 | NM_014747 | regulating synaptic membrane exocytosis 3 | MT1M | NM_176870 | metallothionein 1M | MAP6D1 | NM_024871 | MAP6 domain containing 1 |
| CITED4 | NM_133467 | Cbp/p300 interacting transactivator with Glu/Asp rich carboxy-terminal domain 4 | MT1G | NM_001301267 | metallothionein 1G | LOC344887 | NR_033752 | NmrA-like family domain containing 1 pseudogene |
| CCDC24 | NM_152499 | coiled-coil domain containing 24 | MT1H | NM_005951 | metallothionein 1H | ETV5 | NM_004454 | ETS variant 5 |
| ERI3 | NM_001301698 | ERI1 exoribonuclease family member 3 | CCL22 | NM_002990 | C-C motif chemokine ligand 22 | CLDN1 | NM_021101 | claudin 1 |
| TMEM53 | NM_001300748 | transmembrane protein 53 | GINS3 | NM_001126129 | GINS complex subunit 3 | APOD | NM_001647 | apolipoprotein D |
| POMGNT1 | NM_001290130 | protein O-linked mannose N-acetylglucosaminyltransferase 1 (beta 1,2-) | NDRG4 | NM_020465 | NDRG family member 4 | MUC20 | NM_020790 | mucin 20, cell surface associated |
| ACOT11 | NM_147161 | acyl-CoA thioesterase 11 | BEAN1 | NM_001197224 | brain expressed, associated with NEDD4, 1 | SDHAP1 | NR_003264 | succinate dehydrogenase complex flavoprotein subunit A pseudogene 1 |
| PPAP2B | NM_003713 |  | RRAD | NM_001128850 | Ras-related associated with diabetes | TM4SF19-TCTEX1D2 | NR_037950 | TM4SF19-TCTEX1D2 readthrough (NMD candidate) |
| FGGY | NM_001113411 | FGGY carbohydrate kinase domain containing | NQO1 | NM_000903 | NAD(P)H quinone dehydrogenase 1 | TM4SF19-AS1 | NR_121665 | TM4SF19 antisense RNA 1 |
| DOCK7 | NM_001272000 | dedicator of cytokinesis 7 | BCAR1 | NM_001170717 | BCAR1, Cas family scaffolding protein | TM4SF19 | NM_001204897 | transmembrane 4 L six family member 19 |
| GNG12 | NM_018841 | G protein subunit gamma 12 | GCSH | NR_033249 | glycine cleavage system protein H | IDUA | NM_000203 | iduronidase, alpha-L- |
| CTH | NM_001902 | cystathionine gamma-lyase | OSGIN1 | NM_182981 | oxidative stress induced growth inhibitor 1 | NAT8L | NM_178557 | N-acetyltransferase 8 like |
| DNAJB4 | NM_007034 | DnaJ heat shock protein family (Hsp40) member B4 | MTHFSD | NM_001159380 | methenyltetrahydrofolate synthetase domain containing | RGS12 | NM_002926 | regulator of G-protein signaling 12 |
| DDAH1 | NM_012137 | dimethylarginine dimethylaminohydrolase 1 | C16orf95 | NM_001195125 | chromosome 16 open reading frame 95 | WFS1 | NM_001145853 | wolframin ER transmembrane glycoprotein |
| CYR61 | NM_001554 | cysteine rich angiogenic inducer 61 | CDT1 | NM_030928 | chromatin licensing and DNA replication factor 1 | CCDC96 | NM_153376 | coiled-coil domain containing 96 |
| F3 | NM_001178096 | coagulation factor III, tissue factor | TUBB3 | NM_001197181 | tubulin beta 3 class III | LGI2 | NM_018176 | leucine-rich repeat LGI family member 2 |
| ALG14 | NM_001305242 | ALG14, UDP-N-acetylglucosaminyltransferase subunit | C17orf97 | NM_001013672 | chromosome 17 open reading frame 97 | ZCCHC4 | NM_024936 | zinc finger CCHC-type containing 4 |
| SNX7 | NM_015976 | sorting nexin 7 | ZMYND15 | NM_001267822 | zinc finger MYND-type containing 15 | APBB2 | NM_001166051 | amyloid beta precursor protein binding family B member 2 |
| TAF13 | NM_005645 | TATA-box binding protein associated factor 13 | LOC284023 | NR_024349 | uncharacterized LOC284023 | UCHL1 | NM_004181 | ubiquitin C-terminal hydrolase L1 |
| GSTM4 | NR_024538 | glutathione S-transferase mu 4 | ALOX15B | NM_001141 | arachidonate 15-lipoxygenase, type B | CDS1 | NM_001263 | CDP-diacylglycerol synthase 1 |
| GSTM3 | NM_000849 | glutathione S-transferase mu 3 (brain) | PIK3R6 | NM_001290211 | phosphoinositide-3-kinase regulatory subunit 6 | SPP1 | NM_001251829 | secreted phosphoprotein 1 |
| LOC100996251 | NR_103777 | uncharacterized LOC100996251 | PMP22 | NM_001281456 | peripheral myelin protein 22 | HPGDS | NM_014485 | hematopoietic prostaglandin D synthase |
| PTGFRN | NM_020440 | prostaglandin F2 receptor inhibitor | TRIM16 | NM_006470 | tripartite motif containing 16 | LOC105377348 | NR_131186 | uncharacterized LOC105377348 |
| LOC101929147 | NR_125974 | uncharacterized LOC101929147 | MEIS3P1 | NR_002211 | Meis homeobox 3 pseudogene 1 | ANK2 | NM_001127493 | ankyrin 2, neuronal |
| PHGDH | NM_006623 | phosphoglycerate dehydrogenase | ATPAF2 | NM_145691 | ATP synthase mitochondrial F1 complex assembly factor 2 | LOC101929762 | NR_125931 | uncharacterized LOC101929762 |
| BOLA1 | NM_016074 | bolA family member 1 | USP32P2 | NR_003554 | ubiquitin specific peptidase 32 pseudogene 2 | FGF2 | NM_002006 | fibroblast growth factor 2 |
| NES | NM_006617 | nestin | TRIM16L | NM_001037330 | tripartite motif containing 16-like | SPRY1 | NM_199327 | sprouty RTK signaling antagonist 1 |
| SLAMF8 | NM_020125 | SLAM family member 8 | SLC47A1 | NM_018242 | solute carrier family 47 member 1 | SLC7A11-AS1 | NR_038380 | SLC7A11 antisense RNA 1 |
| SLAMF9 | NM_033438 | SLAM family member 9 | C17orf51 | NM_001113434 | chromosome 17 open reading frame 51 | SLC7A11 | NM_014331 | solute carrier family 7 member 11 |
| PVRL4 | NM_030916 |  | ERAL1 | NM_005702 | Era like 12S mitochondrial rRNA chaperone 1 | TRIM2 | NM_001130067 | tripartite motif containing 2 |
| OLFML2B | NM_015441 | olfactomedin like 2B | SH3GL1P2 | NR_033420 | SH3 domain containing GRB2 like 1 pseudogene 2 | ANXA2P1 | NR_001562 | annexin A2 pseudogene 1 |
| UCK2 | NM_012474 | uridine-cytidine kinase 2 | CCL7 | NM_006273 | C-C motif chemokine ligand 7 | CPE | NM_001873 | carboxypeptidase E |
| GPR161 | NM_153832 | G protein-coupled receptor 161 | CCL8 | NM_005623 | C-C motif chemokine ligand 8 | LRP2BP | NM_018409 | LRP2 binding protein |
| SFT2D2 | NM_199344 | SFT2 domain containing 2 | CCL13 | NM_005408 | C-C motif chemokine ligand 13 | LOC728613 | NR_003713 | programmed cell death 6 pseudogene |
| C1orf112 | NM_018186 | chromosome 1 open reading frame 112 | CCT6B | NM_001193529 | chaperonin containing TCP1 subunit 6B | MYO10 | NM_012334 | myosin X |
| PRRX1 | NM_022716 | paired related homeobox 1 | NLE1 | NM_001014445 | notchless homolog 1 (Drosophila) | C1QTNF3-AMACR | NR_037951 | C1QTNF3-AMACR readthrough (NMD candidate) |
| FMO4 | NM_002022 | flavin containing monooxygenase 4 | DUSP14 | NM_007026 | dual specificity phosphatase 14 | RAI14 | NM_015577 | retinoic acid induced 14 |
| DARS2 | NM_018122 | aspartyl-tRNA synthetase 2, mitochondrial | C17orf96 | NM_001130677 | chromosome 17 open reading frame 96 | SLC1A3 | NM_001289939 | solute carrier family 1 member 3 |
| RASAL2-AS1 | NR_027982 | RASAL2 antisense RNA 1 | IGFBP4 | NM_001552 | insulin like growth factor binding protein 4 | SEPP1 | NM_001085486 | selenoprotein P, plasma, 1 |
| RASAL2 | NM_170692 | RAS protein activator like 2 | P3H4 | NM_006455 | prolyl 3-hydroxylase family member 4 (non-enzymatic) | ITGA2 | NM_002203 | integrin subunit alpha 2 |
| LAMC1 | NM_002293 | laminin subunit gamma 1 | FKBP10 | NM_021939 | FK506 binding protein 10 | HOMER1 | NM_001277077 | homer scaffolding protein 1 |
| PHLDA3 | NM_012396 | pleckstrin homology like domain family A member 3 | PTRF | NM_012232 | polymerase I and transcript release factor | ATP6AP1L | NM_001017971 | ATPase H+ transporting accessory protein 1 like |
| SHISA4 | NR_030775 | shisa family member 4 | TUBG1 | NM_001070 | tubulin gamma 1 | NUDT12 | NM_001300741 | nudix hydrolase 12 |
| MYBPH | NM_004997 | myosin binding protein H | TUBG2 | NM_016437 | tubulin gamma 2 | LINC01023 | NR_046368 | long intergenic non-protein coding RNA 1023 |
| CHI3L1 | NM_001276 | chitinase 3 like 1 | PLEKHH3 | NR_073573 | pleckstrin homology, MyTH4 and FERM domain containing H3 | TRIM36 | NM_018700 | tripartite motif containing 36 |
| CHIT1 | NM_003465 | chitinase 1 | ITGA3 | NM_002204 | integrin subunit alpha 3 | PPIC | NM_000943 | peptidylprolyl isomerase C |
| CDK18 | NM_212503 | cyclin-dependent kinase 18 | ACSF2 | NM_001288968 | acyl-CoA synthetase family member 2 | ALDH7A1 | NM_001201377 | aldehyde dehydrogenase 7 family member A1 |
| PIGR | NM_002644 | polymeric immunoglobulin receptor | COX11 | NR_027941 | COX11 cytochrome c oxidase copper chaperone | MARCH3 | NM_178450 | membrane associated ring-CH-type finger 3 |
| LAMB3 | NM_001017402 | laminin subunit beta 3 | SEPT4 | NM_004574 | septin 4 | P4HA2-AS1 | NR_047470 | P4HA2 antisense RNA 1 |
| BATF3 | NM_018664 | basic leucine zipper ATF-like transcription factor 3 | WFDC21P | NR_030732 | WAP four-disulfide core domain 21, pseudogene | P4HA2 | NM_001142598 | prolyl 4-hydroxylase subunit alpha 2 |
| CDC42BPA | NM_003607 | CDC42 binding protein kinase alpha | MRC2 | NM_006039 | mannose receptor C type 2 | PDLIM4 | NM_001131027 | PDZ and LIM domain 4 |
| LOC100130093 | NR_024485 |  | ACE | NM_000789 | angiotensin I converting enzyme | LRRTM2 | NM_015564 | leucine rich repeat transmembrane neuronal 2 |
| MT1HL1 | NM_001276687 | metallothionein 1H-like 1 | GPRC5C | NM_022036 | G protein-coupled receptor class C group 5 member C | DNAJC18 | NM_152686 | DnaJ heat shock protein family (Hsp40) member C18 |
| DIP2C | NM_014974 | disco interacting protein 2 homolog C | CDR2L | NM_014603 | cerebellar degeneration related protein 2 like | VTRNA1-1 | NR_026703 | vault RNA 1-1 |
| AKR1C1 | NM_001353 | aldo-keto reductase family 1, member C1 | TRIM47 | NM_033452 | tripartite motif containing 47 | PCDHGC3 | NM_032403 | protocadherin gamma subfamily C, 3 |
| AKR1C2 | NM_001354 | aldo-keto reductase family 1, member C2 | ZACN | NM_180990 | zinc activated ion channel | PCDH1 | NM_032420 | protocadherin 1 |
| AKR1C3 | NM_001253908 | aldo-keto reductase family 1, member C3 | CYGB | NM_134268 | cytoglobin | DPYSL3 | NM_001197294 | dihydropyrimidinase like 3 |
| ASB13 | NR_024581 | ankyrin repeat and SOCS box containing 13 | TBC1D16 | NM_001271845 | TBC1 domain family member 16 | GPX3 | NM_002084 | glutathione peroxidase 3 |
| FRMD4A | NM_018027 | FERM domain containing 4A | PCYT2 | NM_001256435 | phosphate cytidylyltransferase 2, ethanolamine | SH3PXD2B | NM_001308175 | SH3 and PX domains 2B |
| MRC1 | NM_002438 | mannose receptor, C type 1 | PYCR1 | NM_153824 | pyrroline-5-carboxylate reductase 1 | STC2 | NM_003714 | stanniocalcin 2 |
| NRP1 | NM_001244973 | neuropilin 1 | LRRC45 | NM_144999 | leucine rich repeat containing 45 | PDLIM7 | NM_005451 | PDZ and LIM domain 7 |
| C10orf10 | NM_007021 | chromosome 10 open reading frame 10 | COLEC12 | NM_130386 | collectin subfamily member 12 | PXDC1 | NM_183373 | PX domain containing 1 |
| ARHGAP22 | NR_045675 | Rho GTPase activating protein 22 | L3MBTL4 | NM_173464 | l(3)mbt-like 4 (Drosophila) | DSP | NM_001008844 | desmoplakin |
| H2AFY2 | NM_018649 | H2A histone family member Y2 | L3MBTL4-AS1 | NR_110765 | L3MBTL4 antisense RNA 1 | PHACTR1 | NM_030948 | phosphatase and actin regulator 1 |
| PCBD1 | NM_000281 | pterin-4 alpha-carbinolamine dehydratase 1 | PTPRM | NM_001105244 | protein tyrosine phosphatase, receptor type M | NHLRC1 | NM_198586 | NHL repeat containing E3 ubiquitin protein ligase 1 |
| CDH23 | NM_001171931 | cadherin-related 23 | CABLES1 | NR_023359 | Cdk5 and Abl enzyme substrate 1 | HIST1H3D | NM_003530 | histone cluster 1, H3d |
| USP54 | NM_152586 | ubiquitin specific peptidase 54 | CABYR | NM_001308231 | calcium binding tyrosine phosphorylation regulated | ZNF322 | NM_001242799 | zinc finger protein 322 |
| PLAU | NM_002658 | plasminogen activator, urokinase | SIGLEC15 | NM_213602 | sialic acid binding Ig like lectin 15 | NKAPL | NM_001007531 | NFKB activating protein like |
| KCNMA1 | NM_001014797 | potassium calcium-activated channel subfamily M alpha 1 | MEX3D | NM_001174118 | mex-3 RNA binding family member D | NFKBIL1 | NM_001144962 | NFKB inhibitor like 1 |
| ZMIZ1-AS1 | NR_024429 | ZMIZ1 antisense RNA 1 | DIRAS1 | NM_145173 | DIRAS family GTP binding RAS like 1 | CFB | NM_001710_3 | complement factor B |
| CH25H | NM_003956 | cholesterol 25-hydroxylase | MFSD12 | NM_174983 | major facilitator superfamily domain containing 12 | PPT2 | NM_138717_3 | palmitoyl-protein thioesterase 2 |
| ANKRD1 | NM_014391 | ankyrin repeat domain 1 | SEMA6B | NM_032108 | semaphorin 6B | TREM2 | NM_018965 | triggering receptor expressed on myeloid cells 2 |
| MORN4 | NM_001098831 | MORN repeat containing 4 | C3 | NM_000064 | complement component 3 | PEX6 | NM_000287 | peroxisomal biogenesis factor 6 |
| AVPI1 | NM_021732 | arginine vasopressin induced 1 | TRIP10 | NR_110231 | thyroid hormone receptor interactor 10 | CUL7 | NM_001168370 | cullin 7 |
| PKD2L1 | NM_001253837 | polycystin 2 like 1, transient receptor potential cation channel | CERS4 | NM_024552 | ceramide synthase 4 | TDRD6 | NM_001010870 | tudor domain containing 6 |
| SFXN2 | NM_178858 | sideroflexin 2 | ANGPTL4 | NR_104213 | angiopoietin like 4 | TRAM2-AS1 | NR_103446 | TRAM2 antisense RNA 1 (head to head) |
| HTRA1 | NM_002775 | HtrA serine peptidase 1 | ZNF561-AS1 | NR_122038 | ZNF561 antisense RNA 1 (head to head) | RAB23 | NM_001278666 | RAB23, member RAS oncogene family |
| DOCK1 | NM_001380 | dedicator of cytokinesis 1 | FDX1L | NM_001031734 | ferredoxin 1-like | ME1 | NM_002395 | malic enzyme 1 |
| SPRN | NM_001012508 | shadow of prion protein homolog (zebrafish) | QTRT1 | NM_031209 | queuine tRNA-ribosyltransferase catalytic subunit 1 | POPDC3 | NM_022361 | popeye domain containing 3 |
| IFITM10 | NM_001170820 | interferon induced transmembrane protein 10 | DOCK6 | NM_020812 | dedicator of cytokinesis 6 | SMPD2 | NM_003080 | sphingomyelin phosphodiesterase 2 |
| SLC22A18AS | NM_007105 | solute carrier family 22 member 18 antisense | FBXW9 | NM_032301 | F-box and WD repeat domain containing 9 | SLC16A10 | NM_018593 | solute carrier family 16 member 10 |
| PRKCDBP | NM_145040 | protein kinase C delta binding protein | NOTCH3 | NM_000435 | notch 3 | KIAA1919 | NM_153369 |  |
| CYB5R2 | NM_001302826 | cytochrome b5 reductase 2 | GDF15 | NM_004864 | growth differentiation factor 15 | CTGF | NM_001901 | connective tissue growth factor |
| ST5 | NM_213618 | suppression of tumorigenicity 5 | CERS1 | NM_021267 | ceramide synthase 1 | EYA4 | NM_172105 | EYA transcriptional coactivator and phosphatase 4 |
| C11orf16 | NM_020643 | chromosome 11 open reading frame 16 | FAAP24 | NM_001300978 | Fanconi anemia core complex associated protein 24 | ARFGEF3 | NM_020340 | ARFGEF family member 3 |
| NRIP3 | NM_020645 | nuclear receptor interacting protein 3 | LRP3 | NM_002333 | LDL receptor related protein 3 | GINM1 | NM_138785 | glycoprotein integral membrane 1 |
| SCUBE2 | NM_001170690 | signal peptide, CUB domain and EGF like domain containing 2 | HAMP | NM_021175 | hepcidin antimicrobial peptide | LRP11 | NM_032832 | LDL receptor related protein 11 |
| SBF2-AS1 | NR_036485 | SBF2 antisense RNA 1 | PRX | NM_181882 | periaxin | RMND1 | NM_001271937 | required for meiotic nuclear division 1 homolog |
| GALNT18 | NM_198516 | polypeptide N-acetylgalactosaminyltransferase 18 | ITPKC | NM_025194 | inositol-trisphosphate 3-kinase C | ESR1 | NM_001122742 | estrogen receptor 1 |
| PARVA | NM_018222 | parvin alpha | CYP2S1 | NM_030622 | cytochrome P450 family 2 subfamily S member 1 | THBS2 | NM_003247 | thrombospondin 2 |
| TMEM86A | NM_153347 | transmembrane protein 86A | ZNF574 | NM_022752 | zinc finger protein 574 | LOC101927021 | NR_110066 | uncharacterized LOC101927021 |
| NAV2 | NM_001111018 | neuron navigator 2 | APOE | NM_000041 | apolipoprotein E | MICALL2 | NM_182924 | MICAL like 2 |
| TCP11L1 | NM_018393 | t-complex 11 like 1 | APOC1 | NM_001645 | apolipoprotein C1 | AMZ1 | NM_133463 | archaelysin family metallopeptidase 1 |
| C11orf96 | NM_001145033 | chromosome 11 open reading frame 96 | APOC1P1 | NR_028414 | apolipoprotein C1 pseudogene 1 | SCIN | NM_001112706 | scinderin |
| FAM180B | NM_001164379 | family with sequence similarity 180 member B | APOC2 | NM_000483 | apolipoprotein C2 | MACC1 | NM_182762 | metastasis associated in colon cancer 1 |
| TNKS1BP1 | NM_033396 | tankyrase 1 binding protein 1 | RTN2 | NM_005619 | reticulon 2 | IL6 | NM_000600 | interleukin 6 |
| YPEL4 | NM_145008 | yippee like 4 | CCDC61 | NM_001267723 | coiled-coil domain containing 61 | GPNMB | NM_002510 | glycoprotein nmb |
| RAB3IL1 | NM_001271686 | RAB3A interacting protein like 1 | PPP5C | NM_001204284 | protein phosphatase 5 catalytic subunit | DFNA5 | NM_004403 | DFNA5, deafness associated tumor suppressor |
| C11orf84 | NM_138471 | chromosome 11 open reading frame 84 | DACT3 | NM_001301046 | dishevelled binding antagonist of beta catenin 3 | FKBP9 | NM_001284341 | FK506 binding protein 9 |
| KLC2 | NM_001134775 | kinesin light chain 2 | EHD2 | NM_014601 | EH domain containing 2 | YAE1D1 | NM_001282446 | Yae1 domain containing 1 |
| PC | NM_000920 | pyruvate carboxylase | SEC1P | NR_004401 | secretory blood group 1, pseudogene | RASA4CP | NR_024116 | RAS p21 protein activator 4C, pseudogene |
| CLCF1 | NM_013246 | cardiotrophin-like cytokine factor 1 | HSD17B14 | NM_016246 | hydroxysteroid (17-beta) dehydrogenase 14 | IGFBP3 | NM_001013398 | insulin-like growth factor binding protein 3 |
| FAM86C2P | NR_024249 | family with sequence similarity 86 member C2, pseudogene | PLEKHA4 | NM_001161354 | pleckstrin homology domain containing A4 | PSPH | NM_004577 | phosphoserine phosphatase |
| CCND1 | NM_053056 | cyclin D1 | C19orf73 | NM_018111 | chromosome 19 open reading frame 73 | LOC100996437 | NR_110037 | uncharacterized LOC100996437 |
| FOLR2 | NM_001113534 | folate receptor beta | SCAF1 | NM_021228 | SR-related CTD associated factor 1 | MLXIPL | NM_032951 | MLX interacting protein like |
| ARHGEF17 | NM_014786 | Rho guanine nucleotide exchange factor 17 | POLD1 | NR_046402 | polymerase (DNA) delta 1, catalytic subunit | CLIP2 | NM_032421 | CAP-Gly domain containing linker protein 2 |
| SLCO2B1 | NM_001145212 | solute carrier organic anion transporter family member 2B1 | CTU1 | NM_145232 | cytosolic thiouridylase subunit 1 | GTF2IRD1 | NM_005685 | GTF2I repeat domain containing 1 |
| TSKU | NM_001258210 | tsukushi, small leucine rich proteoglycan | ZNF818P | NR_073396 | zinc finger protein 818, pseudogene | CLDN12 | NM_001185072 | claudin 12 |
| ME3 | NM_006680 | malic enzyme 3 | EPS8L1 | NM_133180 | EPS8 like 1 | COL1A2 | NM_000089 | collagen type I alpha 2 |
| MMP7 | NM_002423 | matrix metallopeptidase 7 | ISOC2 | NM_024710 | isochorismatase domain containing 2 | PDK4 | NM_002612 | pyruvate dehydrogenase kinase 4 |
| MMP10 | NM_002425 | matrix metallopeptidase 10 | ZNF579 | NM_152600 | zinc finger protein 579 | PUS7 | NM_019042 | pseudouridylate synthase 7 (putative) |
| ZC3H12C | NM_033390 | zinc finger CCCH-type containing 12C | ZSCAN5A | NM_024303 | zinc finger and SCAN domain containing 5A | GPR85 | NM_001146266 | G protein-coupled receptor 85 |
| C11orf1 | NM_022761 | chromosome 11 open reading frame 1 | TRAPPC2B | NR_002166 | trafficking protein particle complex 2B | KCP | NM_001135914 | kielin/chordin-like protein |
| DIXDC1 | NM_001278542 | DIX domain containing 1 | ZNF17 | NM_006959 | zinc finger protein 17 | PODXL | NM_001018111 | podocalyxin like |
| PHLDB1 | NM_015157 | pleckstrin homology like domain family B member 1 | ZNF584 | NM_173548 | zinc finger protein 584 | ZNF767P | NR_027789 | zinc finger family member 767, pseudogene |
| ZNF202 | NM_003455 | zinc finger protein 202 | FAM150B | NM_001002919 | family with sequence similarity 150 member B | RPL23AP53 | NR_003572 | ribosomal protein L23a pseudogene 53 |
| HYLS1 | NM_145014 | hydrolethalus syndrome 1 | KCNS3 | NM_001282428 | potassium voltage-gated channel modifier subfamily S member 3 | ARHGEF10 | NM_014629 | Rho guanine nucleotide exchange factor 10 |
| C11orf45 | NM_145013 | chromosome 11 open reading frame 45 | SDC1 | NM_001006946 | syndecan 1 | CLDN23 | NM_194284 | claudin 23 |
| RPL13P5 | NR_002803 | ribosomal protein L13 pseudogene 5 | EFR3B | NM_014971 | EFR3 homolog B | DLC1 | NM_006094 | DLC1 Rho GTPase activating protein |
| ATN1 | NM_001007026 | atrophin 1 | DTNB | NM_001256308 | dystrobrevin beta | MTUS1 | NM_001001931 | microtubule associated tumor suppressor 1 |
| C1S | NM_001734 | complement component 1, s subcomponent | KIF3C | NM_002254 | kinesin family member 3C | LPL | NM_000237 | lipoprotein lipase |
| C1R | NM_001733 | complement C1r subcomponent | MAPRE3 | NM_012326 | microtubule associated protein RP/EB family member 3 | SORBS3 | NM_005775 | sorbin and SH3 domain containing 3 |
| LOC100506159 | NR_104634 | uncharacterized LOC100506159 | EMILIN1 | NM_007046 | elastin microfibril interfacer 1 | LOC389641 | NR_033928 | uncharacterized LOC389641 |
| CLEC9A | NM_207345 | C-type lectin domain family 9 member A | IFT172 | NM_015662 | intraflagellar transport 172 | ADAMDEC1 | NM_001145271 | ADAM-like, decysin 1 |
| RASSF8-AS1 | NR_038227 | RASSF8 atnisense RNA 1 | RBKS | NM_001287580 | ribokinase | HTRA4 | NM_153692 | HtrA serine peptidase 4 |
| RASSF8 | NM_007211 | Ras association domain family member 8 | GEMIN6 | NM_024775 | gem nuclear organelle associated protein 6 | GGH | NM_003878 | gamma-glutamyl hydrolase |
| BHLHE41 | NM_030762 | basic helix-loop-helix family member e41 | MORN2 | NM_001145450 | MORN repeat containing 2 | LACTB2-AS1 | NR_038881 | LACTB2 antisense RNA 1 |
| SSPN | NM_001135823 | sarcospan | HAAO | NM_012205 | 3-hydroxyanthranilate 3,4-dioxygenase | MSC | NM_005098 | musculin |
| ARNTL2 | NM_001248004 | aryl hydrocarbon receptor nuclear translocator like 2 | DYNC2LI1 | NM_015522 | dynein cytoplasmic 2 light intermediate chain 1 | ATP6V0D2 | NM_152565 | ATPase H+ transporting V0 subunit d2 |
| TMEM117 | NM_032256 | transmembrane protein 117 | EPAS1 | NM_001430 | endothelial PAS domain protein 1 | SDC2 | NM_002998 | syndecan 2 |
| ADCY6 | NM_015270 | adenylate cyclase 6 | CNRIP1 | NM_001111101 | cannabinoid receptor interacting protein 1 | BAALC | NM_001024372 | brain and acute leukemia, cytoplasmic |
| CACNB3 | NM_001206917 | calcium voltage-gated channel auxiliary subunit beta 3 | C2orf42 | NM_017880 | chromosome 2 open reading frame 42 | CTHRC1 | NM_138455 | collagen triple helix repeat containing 1 |
| RND1 | NM_014470 | Rho family GTPase 1 | SPR | NM_003124 | sepiapterin reductase (7,8-dihydrobiopterin:NADP+ oxidoreductase) | DCSTAMP | NM_001257317 | dendrocyte expressed seven transmembrane protein |
| PRPF40B | NM_001031698 | pre-mRNA processing factor 40 homolog B | PRADC1 | NM_032319 | protease-associated domain containing 1 | TNFRSF11B | NM_002546 | tumor necrosis factor receptor superfamily member 11b |
| LOC283335 | NR_033854 | uncharacterized LOC283335 | SEMA4F | NM_004263 | ssemaphorin 4F | KHDRBS3 | NM_006558 | KH domain containing, RNA binding, signal transduction associated 3 |
| AAAS | NM_001173466 | aladin WD repeat nucleoporin | POLR1A | NM_015425 | polymerase (RNA) I subunit A | LYNX1 | NM_023946 | Ly6/neurotoxin 1 |
| GPR84 | NM_020370 | G protein-coupled receptor 84 | ZNF2 | NM_001017396 | zinc finger protein 2 | LINC01604 | NR_120682 |  |
| PPP1R1A | NM_006741 | protein phosphatase 1 regulatory inhibitor subunit 1A | FAHD2A | NM_016044 | fumarylacetoacetate hydrolase domain containing 2A | FAM83H | NM_198488 | family with sequence similarity 83 member H |
| METTL7B | NM_152637 | methyltransferase like 7B | GPAT2 | NM_207328 | glycerol-3-phosphate acyltransferase 2, mitochondrial | OPLAH | NM_017570 | 5-oxoprolinase (ATP-hydrolysing) |
| ITGA7 | NM_001144996 | integrin subunit alpha 7 | NEURL3 | NM_001285485 | neuralized E3 ubiquitin protein ligase 3 | HGH1 | NM_016458_1 | HGH1 homolog |
| CYP27B1 | NM_000785 | cytochrome P450 family 27 subfamily B member 1 | LINC01125 | NR_038386 | long intergenic non-protein coding RNA 1125 | PPP1R16A | NM_032902 | protein phosphatase 1 regulatory subunit 16A |
| METTL21B | NM_015433 | methyltransferase like 21B | SULT1C2 | NM_001056 | sulfotransferase family 1C member 2 | MFSD3 | NR_130120 | major facilitator superfamily domain containing 3 |
| SRGAP1 | NM_020762 | SLIT-ROBO Rho GTPase activating protein 1 | SH3RF3 | NM_001099289 | SH3 domain containing ring finger 3 | C9orf66 | NM_152569 | chromosome 9 open reading frame 66 |
| TBC1D30 | NM_015279 | TBC1 domain family member 30 | SEPT10 | NR_047585 | septin 10 | KANK1 | NM_001256876 | KN motif and ankyrin repeat domains 1 |
| CAPS2 | NM_001286547 | calcyphosine 2 | SOWAHC | NM_023016 | sosondowah ankyrin repeat domain family member C | VLDLR | NM_001018056 | very low density lipoprotein receptor |
| CRADD | NM_003805 | CASP2 and RIPK1 domain containing adaptor with death domain | IL1A | NM_000575 | interleukin 1 alpha | PDCD1LG2 | NM_025239 | programmed cell death 1 ligand 2 |
| IGF1 | NM_001111284 | insulin like growth factor 1 | RPL23AP7 | NR_024529 | ribosomal protein L23a pseudogene 7 | CDKN2A | NM_000077 | cyclin-dependent kinase inhibitor 2A |
| SLC41A2 | NM_032148 | solute carrier family 41 member 2 | SLC35F5 | NR_104470 | solute carrier family 35 member F5 | TUSC1 | NM_001004125 | tumor suppressor candidate 1 |
| ALDH1L2 | NR_027752 | aldehyde dehydrogenase 1 family member L2 | TMEM37 | NM_183240 | transmembrane protein 37 | DNAJB5 | NM_012266 | DnaJ heat shock protein family (Hsp40) member B5 |
| FICD | NM_007076 | FIC domain containing | TMEM177 | NM_001105199 | transmembrane protein 177 | UNC13B | NM_006377 | unc-13 homolog B (C. elegans) |
| ALKBH2 | NM_001205180 | alkB homolog 2, alpha-ketoglutarate-dependent dioxygenase | TFCP2L1 | NM_014553 | transcription factor CP2-like 1 | FBXO10 | NM_012166 | F-box protein 10 |
| TRPV4 | NM_001177428 | transient receptor potential cation channel subfamily V member 4 | RAB6C | NM_032144 | RAB6C, member RAS oncogene family | SHB | NM_003028 | SH2 domain containing adaptor protein B |
| SDSL | NM_001304993 | serine dehydratase like | TMEM163 | NM_030923 | transmembrane protein 163 | APBA1 | NM_001163 | amyloid beta precursor protein binding family A member 1 |
| PXN-AS1 | NR_038924 | PXN antisense RNA 1 | RND3 | NM_001254738 | Rho family GTPase 3 | GADD45G | NM_006705 | growth arrest and DNA damage inducible gamma |
| RILPL1 | NM_178314 | Rab interacting lysosomal protein-like 1 | BBS5 | NM_152384 | Bardet-Biedl syndrome 5 | FANCC | NM_001243743 | Fanconi anemia complementation group C |
| TCTN2 | NM_024809 | tectonic family member 2 | NCKAP1 | NM_205842 | NCK associated protein 1 | ANKS6 | NM_173551 | ankyrin repeat and sterile alpha motif domain containing 6 |
| PXMP2 | NM_018663 | peroxisomal membrane protein 2 | MYO1B | NM_001130158 | myosin IB | GALNT12 | NM_024642 | polypeptide N-acetylgalactosaminyltransferase 12 |
| FLT1 | NM_002019 | fms related tyrosine kinase 1 | IDH1-AS1 | NR_046452 | IDH1 antisense RNA 1 | TMEFF1 | NM_003692 | transmembrane protein with EGF like and two follistatin like domains 1 |
| STARD13 | NM_052851 | StAR related lipid transfer domain containing 13 | MREG | NM_018000 | melanoregulin | ZNF462 | NM_021224 | zinc finger protein 462 |
| NDFIP2 | NM_019080 | Nedd4 family interacting protein 2 | CHPF | NM_001195731 | chondroitin polymerizing factor | PTGR1 | NM_001146109 | prostaglandin reductase 1 |
| SPRY2 | NM_005842 | sprouty RTK signaling antagonist 2 | ARMC9 | NM_025139 | armadillo repeat containing 9 | HDHD3 | NM_001304510 | haloacid dehalogenase like hydrolase domain containing 3 |
| COL4A2 | NM_001846 | collagen type IV alpha 2 | KIF1A | NM_004321 | kinesin family member 1A | TNFSF15 | NM_005118 | tumor necrosis factor superfamily member 15 |
| LINC00346 | NR_027701 | long intergenic non-protein coding RNA 346 | SDCBP2 | NM_080489 | syndecan binding protein 2 | DAB2IP | NM_032552 | DAB2 interacting protein |
| GAS6-AS1 | NR_044995 | GAS6 antisense RNA 1 | FKBP1A-SDCBP2 | NR_037661 | FKBP1A-SDCBP2 readthrough (NMD candidate) | STXBP1 | NM_003165 | syntaxin binding protein 1 |
| MMP14 | NM_004995 | matrix metallopeptidase 14 | ZNF133 | NM_001283003 | zinc finger protein 133 | PTRH1 | NM_001002913 | peptidyl-tRNA hydrolase 1 homolog |
| BCL2L2 | NM_004050 | BCL2 like 2 | NANP | NM_152667 | N-acetylneuraminic acid phosphatase | CERCAM | NM_001286760 | cerebral endothelial cell adhesion molecule |
| FERMT2 | NM_001134999 | fermitin family member 2 | TPX2 | NM_012112 | TPX2, microtubule-associated | LINC00963 | NR_038955 | long intergenic non-protein coding RNA 963 |
| SAMD4A | NM_015589 | sterile alpha motif domain containing 4A | NECAB3 | NM_031231 | N-terminal EF-hand calcium binding protein 3 | PTGES | NM_004878 | prostaglandin E synthase |
| WDHD1 | NM_007086 | WD repeat and HMG-box DNA binding protein 1 | PIGU | NM_080476 | phosphatidylinositol glycan anchor biosynthesis class U | NCS1 | NM_014286 | neuronal calcium sensor 1 |
| HIF1A-AS1 | NR_047116 | HIF1A antisense RNA 1 | PROCR | NM_006404 | protein C receptor | GPSM1 | NM_001145638 | G-protein signaling modulator 1 |
| PLEK2 | NM_016445 | pleckstrin 2 | EPB41L1 | NM_001258331 | erythrocyte membrane protein band 4.1 like 1 | EGFL7 | NR_046367 | EGF like domain multiple 7 |
| ACOT4 | NM_152331 | acyl-CoA thioesterase 4 | TGM2 | NM_004613 | transglutaminase 2 | NOXA1 | NM_001256067 | NADPH oxidase activator 1 |
| PTGR2 | NM_001146154 | prostaglandin reductase 2 | LPIN3 | NR_126051 | lipin 3 | PNPLA7 | NM_152286 | patatin like phospholipase domain containing 7 |
| LTBP2 | NM_000428 | latent transforming growth factor beta binding protein 2 | EMILIN3 | NM_052846 | elastin microfibril interfacer 3 | CD99P1 | NR_033381 | CD99 molecule pseudogene 1 |
| RPS6KL1 | NM_031464 | ribosomal protein S6 kinase like 1 | ZSWIM3 | NR_037628 | zinc finger SWIM-type containing 3 | ANOS1 | NM_000216 | anosmin 1 |
| IFT43 | NR_045664 | intraflagellar transport 43 | LAMA5 | NM_005560 | laminin subunit alpha 5 | PIR | NM_001018109 | pirin |
| EFCAB11 | NM_001284267 | EF-hand calcium binding domain 11 | SLCO4A1 | NM_016354 | solute carrier organic anion transporter family member 4A1 | APOO | NR_026545 | apolipoprotein O |
| KCNK13 | NM_022054 | potassium two pore domain channel subfamily K member 13 | TNFRSF6B | NM_003823 | tumor necrosis factor receptor superfamily member 6b | ZNF711 | NM_021998 | zinc finger protein 711 |
| UNC79 | NM_020818 | unc-79 homolog (C. elegans) | CBR3 | NM_001236 | carbonyl reductase 3 | NOX1 | NM_007052 | NADPH oxidase 1 |
| CKB | NM_001823 | creatine kinase B | BACE2 | NM_138991 | beta-site APP-cleaving enzyme 2 | TIMM8A | NM_004085 | translocase of inner mitochondrial membrane 8 homolog A (yeast) |
| AHNAK2 | NM_138420 | AHNAK nucleoprotein 2 | CBS | NM_001178009 | cystathionine-beta-synthase | ARMCX1 | NM_016608 | armadillo repeat containing, X-linked 1 |
| TMEM121 | NM_025268 | transmembrane protein 121 | LRRC3 | NM_030891 | leucine rich repeat containing 3 | ARMCX2 | NM_001282231 | armadillo repeat containing, X-linked 2 |
| TJP1 | NM_175610 | tight junction protein 1 | COL6A1 | NM_001848 | collagen type VI alpha 1 | GPRASP2 | NM_001004051 | G protein-coupled receptor associated sorting protein 2 |
| SCG5 | NM_001144757 | secretogranin V | YBEY | NM_001006114 | ybeY metallopeptidase (putative) | PLS3 | NM_005032 | plastin 3 |
| PGBD4 | NM_152595 | piggyBac transposable element derived 4 | CECR2 | NM_001290046 | cat eye syndrome chromosome region, candidate 2 | SOWAHD | NM_001105576 | sosondowah ankyrin repeat domain family member D |
| GPR176 | NM_007223 | G protein-coupled receptor 176 | DGCR6 | NM_005675 | DiGeorge syndrome critical region gene 6 | TMEM255A | NM_001104544 | transmembrane protein 255A |
| SRP14-AS1 | NR_040060 | SRP14 antisense RNA1 (head to head) | GSTT2 | NR_126445 | glutathione S-transferase theta 2 (gene/pseudogene) | GPC4 | NM_001448 | glypican 4 |
| GCHFR | NM_005258 | GTP cyclohydrolase I feedback regulator | TTC28 | NM_001145418 | tetratricopeptide repeat domain 28 | FAM127C | NM_001078173 | family with sequence similarity 127 member C |
| CHAC1 | NM_024111 | ChaC glutathione-specific gamma-glutamylcyclotransferase 1 | RHBDD3 | NM_012265 | rhomboid domain containing 3 | MAMLD1 | NM_001177465 | mastermind like domain containing 1 |
| TYRO3 | NM_006293 | TYRO3 protein tyrosine kinase | NEFH | NM_021076 | neurofilament, heavy polypeptide | BGN | NM_001711 | biglycan |
| ADAL | NM_001012969 | adenosine deaminase-like | LIF | NM_001257135 | leukemia inhibitory factor | CH17-340M24.3 | NR_103768 | uncharacterized protein BC009467 |
| ELL3 | NM_025165 | elongation factor for RNA polymerase II 3 | SEC14L2 | NM_033382 | SEC14 like lipid binding 2 | CLIC2 | NM_001289 | chloride intracellular channel 2 |
| GATM | NM_001482 | glycine amidinotransferase | SLC35E4 | NM_001001479 | solute carrier family 35 member E4 | RPS4Y1 | NM_001008 | ribosomal protein S4, Y-linked 1 |
| TNFAIP8L3 | NM_207381 | TNF alpha induced protein 8 like 3 | SELM | NM_080430 | selenoprotein M | ZFY | NM_001145276 | zinc finger protein, Y-linked |
| CYP19A1 | NM_031226 | cytochrome P450 family 19 subfamily A member 1 | TIMP3 | NM_000362 | TIMP metallopeptidase inhibitor 3 | PRKY | NR_028062 | protein kinase, Y-linked, pseudogene |
| ALDH1A2 | NM_170696 | aldehyde dehydrogenase 1 family member A2 | RBFOX2 | NM_014309 | RNA binding protein, fox-1 homolog 2 | TTTY15 | NR_001545 | testis-specific transcript, Y-linked 15 (non-protein coding) |
| SMAD6 | NR_027654 | SMAD family member 6 | FOXRED2 | NM_024955 | FAD dependent oxidoreductase domain containing 2 | USP9Y | NM_004654 | ubiquitin specific peptidase 9, Y-linked |
| PAQR5 | NM_017705 | progestin and adipoQ receptor family member 5 | PICK1 | NM_001039584 | protein interacting with PRKCA 1 | DDX3Y | NM_001122665 | DEAD-box helicase 3, Y-linked |
| LARP6 | NM_001286679 | La ribonucleoprotein domain family member 6 | CBY1 | NM_015373 | chibby homolog 1 (Drosophila) | UTY | NR_047643 | ubiquitously transcribed tetratricopeptide repeat containing, Y-linked |
| CD276 | NM_025240 | CD276 molecule | FAM109B | NM_001002034 | family with sequence similarity 109 member B | TXLNGY | NR_045128 | taxilin gamma pseudogene, Y-linked |
| LOXL1 | NM_005576 | lysyl oxidase like 1 | MCAT | NR_046423 | malonyl-CoA-acyl carrier protein transacylase | KDM5D | NM_004653 | lysine demethylase 5D |
| RPP25 | NM_017793 | ribonuclease P/MRP subunit p25 | PRR5 | NM_001017529 | proline rich 5 | EIF1AY | NM_001278612 | eukaryotic translation initiation factor 1A, Y-linked |

**Supplemental Table 5. GO analysis and KEGG pathways in 705 genes shared by iMGs and brain-MG.**

| **Category** | **Term** | **Count** | **Genes** |
| --- | --- | --- | --- |
| GOTERM_BP_DIRECT | GO:0030198~extracellular matrix organization | 23 | COL4A2, OLFML2B, NOX1, CCDC80, HSPG2, ITGA2, ITGA3, EMILIN1, LAMB3, TNFRSF11B, COL9A2, BGN, LAMB2, LAMA5, ITGA7, COL1A2, COL6A1, LAMC1, APBB2, FGF2, LOXL1, SPP1, CYR61 |
| GOTERM_BP_DIRECT | GO:0006693~prostaglandin metabolic process | 7 | AKR1C3, AKR1C2, PTGR1, PTGR2, PDPN, PTGES, HPGDS |
| GOTERM_BP_DIRECT | GO:0055114~oxidation-reduction process | 42 | ME1, ME3, CYB5R2, HSD3B7, HSD17B14, UTY, CYP2S1, OSGIN1, ALDH1L2, AKR1C3, FMO4, ALDH1A2, AKR1C2, CYP27B1, P4HA2, PIR, CH25H, GPX3, HAAO, SPR, NQO1, LOXL1, AKR1C1, KDM5D, CYP19A1, SH3PXD2B, PTGR1, PTGR2, PCBD1, FOXRED2, NOX1, DECR2, CBR3, PYCR1, ALDH7A1, ALOX15B, PHGDH, KDM8, ASPHD1, FDX1L, MECR, CBS |
| GOTERM_BP_DIRECT | GO:0001523~retinoid metabolic process | 11 | AKR1C3, GPC4, LPL, SDC1, RBP1, APOE, HSPG2, APOC2, AKR1C1, SDC2, SDC3 |
| GOTERM_BP_DIRECT | GO:0070374~positive regulation of ERK1 and ERK2 cascade | 18 | TNFAIP8L3, IL6, NRP1, CHI3L1, CCL8, PRKCDBP, CCL7, SPRY2, CCL22, CCL13, CTGF, NDRG4, TRPV4, FAM150B, GPNMB, TREM2, ST5, FGF2 |
| GOTERM_BP_DIRECT | GO:0016477~cell migration | 17 | CTHRC1, FLT1, USP9Y, BCAR1, PODXL, NOX1, CSPG4, EPHA2, SDC2, SDC3, SDC1, DOCK1, CTGF, LAMA5, CDC42BPA, LAMC1, EMP2 |
| GOTERM_BP_DIRECT | GO:0006027~glycosaminoglycan catabolic process | 7 | GPC4, HYAL2, SDC1, HSPG2, SDC2, IDUA, SDC3 |
| GOTERM_BP_DIRECT | GO:0030203~glycosaminoglycan metabolic process | 7 | GPC4, SDC1, BGN, CSPG4, HSPG2, SDC2, SDC3 |
| GOTERM_BP_DIRECT | GO:0001525~angiogenesis | 19 | COL4A2, DAB2IP, NRP1, FLT1, EPAS1, EGFL7, TNFRSF12A, NOX1, HSPG2, CSPG4, MMP14, MMP2, SHB, ARHGAP22, APOD, CTGF, LAMA5, PIK3R6, ANGPTL4 |
| GOTERM_BP_DIRECT | GO:0050729~positive regulation of inflammatory response | 10 | LPL, HYAL2, CCL13, TGM2, CCL8, TRPV4, ITGA2, GPRC5B, CCL7, IL17RB |
| GOTERM_BP_DIRECT | GO:0022617~extracellular matrix disassembly | 10 | SH3PXD2B, MMP10, LAMB3, HTRA1, HSPG2, MMP7, LAMC1, MMP14, MMP2, SPP1 |
| GOTERM_BP_DIRECT | GO:0001558~regulation of cell growth | 10 | EPB41L1, HTRA1, CTGF, BCAR1, H2AFY2, HTRA4, IGFBP3, IGFBP4, IL17RB, CYR61 |
| GOTERM_BP_DIRECT | GO:0043627~response to estrogen | 9 | GSTM3, TNFRSF11B, CCND1, CYP27B1, SMAD6, PPARG, ESR1, MMP14, CITED4 |
| GOTERM_BP_DIRECT | GO:0043410~positive regulation of MAPK cascade | 10 | LIF, TNFRSF6B, DAB2IP, SORBS3, TNFRSF11B, IL6, FLT1, IGF1, IGFBP3, IGFBP4 |
| GOTERM_BP_DIRECT | GO:0035987~endodermal cell differentiation | 6 | COL4A2, LAMB3, ITGA7, COL6A1, MMP14, MMP2 |
| GOTERM_BP_DIRECT | GO:0032966~negative regulation of collagen biosynthetic process | 4 | IL6, PPARG, CYGB, ERRFI1 |
| GOTERM_BP_DIRECT | GO:0007155~cell adhesion | 28 | BCAR1, PCDHGC3, SORBS3, LAMB3, LAMB2, CTGF, DGCR6, MYBPH, COL6A1, GPNMB, THBS2, APBA1, SPP1, CYR61, TYRO3, EGFL7, PODXL, ITGA2, ITGA3, CERCAM, SSPN, EMILIN1, RND3, ITGA7, ANOS1, LAMC1, EMP2, PARVA |
| GOTERM_BP_DIRECT | GO:0071347~cellular response to interleukin-1 | 9 | HYAL2, DAB2IP, CCL13, IL6, CCL22, CHI3L1, CCL8, ANKRD1, CCL7 |
| GOTERM_BP_DIRECT | GO:0002548~monocyte chemotaxis | 7 | CCL13, IL6, CCL22, FLT1, FOLR2, CCL8, CCL7 |
| GOTERM_BP_DIRECT | GO:0048012~hepatocyte growth factor receptor signaling pathway | 4 | NRP1, BCAR1, MUC20, MST1 |
| GOTERM_BP_DIRECT | GO:0045926~negative regulation of growth | 5 | MT1L, MT1M, ALOX15B, MT1H, MT1G |
| GOTERM_BP_DIRECT | GO:0071356~cellular response to tumor necrosis factor | 11 | HYAL2, DAB2IP, CCL13, IL6, CCL22, HAMP, CHI3L1, CCL8, ANKRD1, CCL7, DCSTAMP |
| GOTERM_BP_DIRECT | GO:0045765~regulation of angiogenesis | 6 | IL6, TNFRSF12A, GPNMB, EMP2, FGF2, EPHA2 |
| GOTERM_BP_DIRECT | GO:0030324~lung development | 9 | ALDH1A2, EPAS1, PDPN, LAMA5, CTGF, CHI3L1, ITGA3, MMP14, FGF2 |
| GOTERM_BP_DIRECT | GO:0034447~very-low-density lipoprotein particle clearance | 3 | APOE, APOC1, VLDLR |
| GOTERM_BP_DIRECT | GO:0030574~collagen catabolic process | 8 | MMP10, COL4A2, MRC2, COL1A2, MMP7, COL6A1, MMP14, MMP2 |
| GOTERM_BP_DIRECT | GO:0043407~negative regulation of MAP kinase activity | 6 | SPRY2, SPRY1, HYAL2, DAB2IP, APOE, UCHL1 |
| GOTERM_BP_DIRECT | GO:0071395~cellular response to jasmonic acid stimulus | 3 | AKR1C3, AKR1C2, AKR1C1 |
| GOTERM_BP_DIRECT | GO:0042157~lipoprotein metabolic process | 6 | LPL, SDC1, APOE, APOC1, HSPG2, APOC2 |
| GOTERM_BP_DIRECT | GO:0034446~substrate adhesion-dependent cell spreading | 6 | MICALL2, TYRO3, LAMA5, FERMT2, LAMC1, PARVA |
| GOTERM_BP_DIRECT | GO:0070328~triglyceride homeostasis | 5 | LPL, APOE, MLXIPL, APOC2, ANGPTL4 |
| GOTERM_BP_DIRECT | GO:0045618~positive regulation of keratinocyte differentiation | 4 | CYP27B1, ALOX15B, H2AFY2, TRIM16 |
| GOTERM_BP_DIRECT | GO:0007566~embryo implantation | 6 | LIF, STC2, MST1, EMP2, MMP2, SPP1 |
| GOTERM_BP_DIRECT | GO:0009612~response to mechanical stimulus | 7 | LRP11, PPARG, CHI3L1, TRPV4, MMP14, PSPH, SMPD2 |
| GOTERM_BP_DIRECT | GO:0045669~positive regulation of osteoblast differentiation | 7 | CTHRC1, IL6, PDLIM7, CD276, IGF1, FGF2, CYR61 |
| GOTERM_BP_DIRECT | GO:0048661~positive regulation of smooth muscle cell proliferation | 7 | NOTCH3, IL6, NOX1, TGM2, IGF1, ITGA2, FGF2 |
| GOTERM_BP_DIRECT | GO:0007165~signal transduction | 52 | DLC1, GPR85, NRP1, PPARG, TNFSF15, RRAD, RASSF8, CRADD, ARHGAP22, SHB, TNFRSF11B, ANK2, PTGES, PPP1R1A, FGF2, TYRO3, PTPRM, PDPN, ESR1, CLIC2, CDS1, PLEKHH3, SRGAP3, UNC13B, SRGAP1, C3, CCL8, NMB, GNG12, CCL7, STARD13, RASAL2, CCL22, DOCK1, RGS12, TRIP10, HPGDS, MRC1, EPAS1, MRC2, NOX1, NDFIP2, IGF1, MAPK11, RPS6KL1, PEX11A, MYO10, CCL13, GDF15, PLAU, IGFBP4, VLDLR |
| GOTERM_BP_DIRECT | GO:0034382~chylomicron remnant clearance | 3 | APOE, APOC1, APOC2 |
| GOTERM_BP_DIRECT | GO:0007229~integrin-mediated signaling pathway | 9 | DOCK1, LAMA5, CTGF, BCAR1, FERMT2, ITGA7, ITGA2, ITGA3, ADAMDEC1 |
| GOTERM_BP_DIRECT | GO:0030335~positive regulation of cell migration | 13 | FLT1, BCAR1, PODXL, IGF1, MMP14, CCL7, SEMA6B, SEMA4F, F3, FAM83H, GPNMB, PLAU, CYR61 |
| GOTERM_BP_DIRECT | GO:0071276~cellular response to cadmium ion | 4 | AKR1C3, MT1H, MT1G, PPP5C |
| GOTERM_BP_DIRECT | GO:0043065~positive regulation of apoptotic process | 18 | KCNMA1, SEPT4, DAB2IP, IL6, TNFRSF12A, ARHGEF17, OSGIN1, ANKRD1, ALDH1A2, CDKN2A, NUPR1, GADD45G, SCIN, TGM2, APBB2, IGFBP3, UNC13B, PHLDA3 |
| GOTERM_BP_DIRECT | GO:0051897~positive regulation of protein kinase B signaling | 8 | AKR1C3, SPRY2, TNFAIP8L3, AKR1C2, IL6, F3, CHI3L1, FGF2 |
| GOTERM_BP_DIRECT | GO:0001974~blood vessel remodeling | 5 | LIF, ACE, BGN, EPAS1, TGM2 |
| GOTERM_BP_DIRECT | GO:0033189~response to vitamin A | 4 | ALDH1A2, RBP1, HAMP, PPARG |
| GOTERM_BP_DIRECT | GO:0070050~neuron cellular homeostasis | 3 | TYRO3, FGGY, HAAO |
| GOTERM_BP_DIRECT | GO:0034372~very-low-density lipoprotein particle remodeling | 3 | LPL, APOE, APOC2 |
| GOTERM_BP_DIRECT | GO:0071294~cellular response to zinc ion | 4 | MT1L, MT1M, MT1H, MT1G |
| GOTERM_BP_DIRECT | GO:0010976~positive regulation of neuron projection development | 8 | DAB2IP, IL6, NDRG4, DPYSL3, ITGA3, ANKRD1, FAM150B, GPRC5B |
| GOTERM_BP_DIRECT | GO:0046697~decidualization | 4 | LIF, CYP27B1, STC2, SPP1 |
| GOTERM_BP_DIRECT | GO:0044598~doxorubicin metabolic process | 3 | AKR1C3, AKR1C2, AKR1C1 |
| GOTERM_BP_DIRECT | GO:0044597~daunorubicin metabolic process | 3 | AKR1C3, AKR1C2, AKR1C1 |
| GOTERM_BP_DIRECT | GO:0010764~negative regulation of fibroblast migration | 3 | HYAL2, CYGB, FGF2 |
| GOTERM_BP_DIRECT | GO:0009409~response to cold | 5 | LPL, IL6, LRP11, ACOT11, PPARG |
| GOTERM_BP_DIRECT | GO:0048010~vascular endothelial growth factor receptor signaling pathway | 7 | SHB, DOCK1, NRP1, FLT1, BCAR1, MAPK11, NCKAP1 |
| GOTERM_BP_DIRECT | GO:0048844~artery morphogenesis | 4 | NOTCH3, NRP1, APOE, PRRX1 |
| GOTERM_BP_DIRECT | GO:0008285~negative regulation of cell proliferation | 21 | DLC1, DFNA5, DAB2IP, IL6, SMAD6, LIF, SPRY2, ALDH1A2, TNFRSF9, SPRY1, CDKN2A, CYP27B1, PTGES, ALOX15B, SCIN, FABP3, PMP22, GPNMB, IGFBP3, FGF2, IL1A |
| GOTERM_BP_DIRECT | GO:0045766~positive regulation of angiogenesis | 9 | FLT1, C3, F3, CHI3L1, PIK3R6, FGF2, DDAH1, IL1A, ANGPTL4 |
| GOTERM_BP_DIRECT | GO:0042448~progesterone metabolic process | 3 | AKR1C3, AKR1C2, AKR1C1 |
| GOTERM_BP_DIRECT | GO:0048245~eosinophil chemotaxis | 3 | CCL13, HRH1, CCL7 |
| GOTERM_BP_DIRECT | GO:0010863~positive regulation of phospholipase C activity | 3 | FLT1, ESR1, FGF2 |
| GOTERM_BP_DIRECT | GO:0043589~skin morphogenesis | 3 | COL1A2, ITGA2, ERRFI1 |
| GOTERM_BP_DIRECT | GO:0035924~cellular response to vascular endothelial growth factor stimulus | 4 | DAB2IP, NRP1, FLT1, MT1G |
| GOTERM_BP_DIRECT | GO:0008360~regulation of cell shape | 10 | DLC1, MYO10, CCL13, IL6, PDPN, FERMT2, ITGA7, FGD5, CCL7, PARVA |
| GOTERM_BP_DIRECT | GO:0051592~response to calcium ion | 6 | KCNMA1, IL6, CCND1, SDC1, PTGES, HOMER1 |
| GOTERM_BP_DIRECT | GO:0070373~negative regulation of ERK1 and ERK2 cascade | 6 | LIF, SPRY2, SPRY1, DAB2IP, ERRFI1, TIMP3 |
| GOTERM_BP_DIRECT | GO:0035414~negative regulation of catenin import into nucleus | 3 | DAB2IP, DACT3, WWTR1 |
| GOTERM_BP_DIRECT | GO:0046888~negative regulation of hormone secretion | 3 | LIF, IL6, NMB |
| GOTERM_BP_DIRECT | GO:0019371~cyclooxygenase pathway | 3 | AKR1C3, PTGES, HPGDS |
| GOTERM_BP_DIRECT | GO:0048870~cell motility | 4 | PHACTR1, PDPN, MMP14, EPHA2 |
| GOTERM_BP_DIRECT | GO:0048041~focal adhesion assembly | 4 | DLC1, LAMA5, FERMT2, ITGA2 |
| GOTERM_BP_DIRECT | GO:0042593~glucose homeostasis | 8 | IL6, WFS1, PDK4, PPARG, MLXIPL, TRPV4, NMB, GPRC5B |
| GOTERM_BP_DIRECT | GO:0046580~negative regulation of Ras protein signal transduction | 4 | RASAL2, SPRY2, SPRY1, DAB2IP |
| GOTERM_BP_DIRECT | GO:0006024~glycosaminoglycan biosynthetic process | 5 | GPC4, SDC1, HSPG2, SDC2, SDC3 |
| GOTERM_BP_DIRECT | GO:0060445~branching involved in salivary gland morphogenesis | 3 | IL6, LAMA5, TGM2 |
| GOTERM_BP_DIRECT | GO:0050999~regulation of nitric-oxide synthase activity | 4 | SPR, CYGB, DDAH1, GCHFR |
| GOTERM_BP_DIRECT | GO:0001649~osteoblast differentiation | 8 | MRC2, COL6A1, WWTR1, GPNMB, IGFBP3, EPHA2, SPP1, CYR61 |
| GOTERM_BP_DIRECT | GO:0010977~negative regulation of neuron projection development | 5 | ADCY6, TRPV4, DPYSL3, PMP22, KANK1 |
| GOTERM_BP_DIRECT | GO:0042632~cholesterol homeostasis | 6 | ACSM3, LPL, APOE, FABP3, APOC2, AKR1C1 |
| GOTERM_BP_DIRECT | GO:0042574~retinal metabolic process | 3 | AKR1C3, ALDH1A2, AKR1C1 |
| GOTERM_BP_DIRECT | GO:0015909~long-chain fatty acid transport | 3 | APOE, PPARG, FABP3 |
| GOTERM_BP_DIRECT | GO:0046330~positive regulation of JNK cascade | 6 | DAB2IP, CTGF, GADD45G, NOX1, TRPV4, IL1A |
| GOTERM_BP_DIRECT | GO:0014047~glutamate secretion | 4 | SLC1A3, STXBP1, UNC13B, APBA1 |
| GOTERM_BP_DIRECT | GO:0070252~actin-mediated cell contraction | 2 | EMP2, PARVA |
| GOTERM_BP_DIRECT | GO:0031345~negative regulation of cell projection organization | 2 | SPRY2, ITGA3 |
| GOTERM_BP_DIRECT | GO:0031532~actin cytoskeleton reorganization | 5 | MICALL2, PHACTR1, CDC42BPA, TRPV4, PARVA |
| GOTERM_BP_DIRECT | GO:0006954~inflammatory response | 19 | TNFRSF6B, IL6, DAB2IP, C3, NOX1, CHI3L1, CCL8, EPHA2, CCL7, TNFRSF9, SDC1, HRH1, CCL22, TNFRSF11B, CCL13, FOLR2, IGFBP4, IL1A, SPP1 |
| GOTERM_BP_DIRECT | GO:0072657~protein localization to membrane | 3 | SH3PXD2B, CPE, FERMT2 |
| GOTERM_BP_DIRECT | GO:0043568~positive regulation of insulin-like growth factor receptor signaling pathway | 3 | IGF1, IGFBP3, IGFBP4 |
| GOTERM_BP_DIRECT | GO:0002526~acute inflammatory response | 3 | IL6, NUPR1, PTGES |
| GOTERM_BP_DIRECT | GO:0006809~nitric oxide biosynthetic process | 3 | SPR, NQO1, GCHFR |
| GOTERM_BP_DIRECT | GO:0055089~fatty acid homeostasis | 3 | APOE, POLD1, MLXIPL |
| GOTERM_BP_DIRECT | GO:0007605~sensory perception of sound | 9 | SPRY2, EYA4, DFNA5, ALDH7A1, TJP1, SLC1A3, WFS1, NAV2, CDH23 |
| GOTERM_BP_DIRECT | GO:0048662~negative regulation of smooth muscle cell proliferation | 4 | APOD, NDRG4, PPARG, IGFBP3 |
| GOTERM_BP_DIRECT | GO:0006629~lipid metabolic process | 10 | PNPLA7, LPL, APOD, ALOX15B, CH25H, PPARG, APOC1, APOC2, ZNF202, PC |
| GOTERM_BP_DIRECT | GO:0021915~neural tube development | 4 | ALDH1A2, PHGDH, CECR2, EPHA2 |
| GOTERM_BP_DIRECT | GO:0060271~cilium morphogenesis | 9 | RILPL1, BBS5, IFT172, RAB23, IFT43, TCTN2, WWTR1, PARVA, IFT140 |
| GOTERM_BP_DIRECT | GO:0030207~chondroitin sulfate catabolic process | 3 | BGN, CSPG4, IDUA |
| GOTERM_BP_DIRECT | GO:0035729~cellular response to hepatocyte growth factor stimulus | 3 | IL6, NRP1, BCAR1 |
| GOTERM_BP_DIRECT | GO:0033700~phospholipid efflux | 3 | APOE, APOC1, APOC2 |
| GOTERM_BP_DIRECT | GO:0006874~cellular calcium ion homeostasis | 7 | CCL13, STC2, ANK2, APOE, CCL8, TRPV4, CCL7 |
| GOTERM_BP_DIRECT | GO:0007015~actin filament organization | 6 | SORBS3, RND1, MYO1B, BCAR1, TRPV4, EMP2 |
| GOTERM_BP_DIRECT | GO:0035024~negative regulation of Rho protein signal transduction | 3 | DLC1, ITGA3, KANK1 |
| GOTERM_BP_DIRECT | GO:0006508~proteolysis | 23 | AMZ1, NRIP3, ACY1, CFB, C3, MST1, MMP7, GGH, C1R, C1S, MMP14, MMP2, C1QC, RHBDD3, MMP10, C1QB, CUL7, HTRA1, BACE2, YBEY, HTRA4, ADAMDEC1, PLAU |
| GOTERM_BP_DIRECT | GO:0006633~fatty acid biosynthetic process | 5 | ACSM3, LPL, CH25H, MCAT, MECR |
| GOTERM_BP_DIRECT | GO:0006631~fatty acid metabolic process | 5 | C3, ACOT11, FABP3, MECR, ACSF2 |
| GOTERM_BP_DIRECT | GO:0051895~negative regulation of focal adhesion assembly | 3 | DLC1, APOD, MMP14 |
| GOTERM_BP_DIRECT | GO:0008152~metabolic process | 10 | ACSM3, GSTM3, GSTM4, DIP2C, CPE, MCAT, HDHD3, ISOC2, FAHD2A, ACSF2 |
| GOTERM_BP_DIRECT | GO:0007412~axon target recognition | 2 | UCHL1, STXBP1 |
| GOTERM_BP_DIRECT | GO:0034371~chylomicron remodeling | 2 | LPL, APOC2 |
| GOTERM_BP_DIRECT | GO:0072592~oxygen metabolic process | 2 | ME3, NOX1 |
| GOTERM_BP_DIRECT | GO:0046320~regulation of fatty acid oxidation | 2 | PDK4, FABP3 |
| GOTERM_BP_DIRECT | GO:0060978~angiogenesis involved in coronary vascular morphogenesis | 2 | NRP1, FGF2 |
| GOTERM_BP_DIRECT | GO:0019343~cysteine biosynthetic process via cystathionine | 2 | CTH, CBS |
| GOTERM_BP_DIRECT | GO:0030308~negative regulation of cell growth | 8 | HYAL2, CDKN2A, CYP27B1, DACT3, PPARG, OSGIN1, APBB2, DCSTAMP |
| GOTERM_BP_DIRECT | GO:0006935~chemotaxis | 8 | CCL13, CCL22, CCL8, ANOS1, FGF2, CCL7, PLAU, CYR61 |
|  |  |  |  |
| **Category** | **Term** | **Count** | **Genes** |
| KEGG_PATHWAY | hsa04512:ECM-receptor interaction | 14 | COL4A2, HSPG2, ITGA2, ITGA3, LAMB3, SDC1, LAMB2, LAMA5, ITGA7, COL1A2, COL6A1, LAMC1, THBS2, SPP1 |
| KEGG_PATHWAY | hsa00260:Glycine, serine and threonine metabolism | 8 | ALDH7A1, CTH, GATM, PHGDH, SDSL, GCSH, PSPH, CBS |
| KEGG_PATHWAY | hsa04510:Focal adhesion | 18 | COL4A2, FLT1, BCAR1, ITGA2, IGF1, ITGA3, LAMB3, CCND1, DOCK1, LAMB2, LAMA5, ITGA7, COL1A2, COL6A1, LAMC1, THBS2, SPP1, PARVA |
| KEGG_PATHWAY | hsa01100:Metabolic pathways | 58 | COX11, CYP2S1, ITPKC, PSPH, GPAT2, ACOT4, CKB, AKR1C3, P4HA2, PTGES, B3GALNT1, SPR, PCYT2, ATP6V0D2, NANP, IDUA, HYAL2, GATM, POLR1A, PIGU, CBR3, PIGQ, CDS1, LPIN3, EARS2, PYCR1, CTH, ALDH7A1, ALOX15B, CHPF, MECR, ME1, ME3, HSD3B7, PPT2, CERS4, ALDH1A2, CYP27B1, CERS1, HAAO, GCSH, GALE, GALNT18, UCK2, GALNT12, HPGDS, CYP19A1, ACY1, MCAT, SDSL, ACSM3, POLD1, PHGDH, NAT8L, SMPD2, PC, ALG14, CBS |
| KEGG_PATHWAY | hsa04610:Complement and coagulation cascades | 8 | C1QB, C3, CFB, F3, C1R, C1S, C1QC, PLAU |
| KEGG_PATHWAY | hsa01230:Biosynthesis of amino acids | 8 | PYCR1, CTH, ACY1, PHGDH, SDSL, PSPH, PC, CBS |
| KEGG_PATHWAY | hsa05133:Pertussis | 8 | C1QB, IL6, C3, MAPK11, C1R, C1S, C1QC, IL1A |
| KEGG_PATHWAY | hsa05222:Small cell lung cancer | 8 | COL4A2, CCND1, LAMB3, LAMB2, LAMA5, ITGA2, ITGA3, LAMC1 |
| KEGG_PATHWAY | hsa05020:Prion diseases | 5 | C1QB, IL6, LAMC1, C1QC, IL1A |
| KEGG_PATHWAY | hsa05150:Staphylococcus aureus infection | 6 | C1QB, C3, CFB, C1R, C1S, C1QC |
| KEGG_PATHWAY | hsa05205:Proteoglycans in cancer | 13 | ESR1, HSPG2, ITGA2, IGF1, MAPK11, TIMP3, MMP2, SDC2, SDC1, CCND1, ANK2, FGF2, PLAU |
| KEGG_PATHWAY | hsa00980:Metabolism of xenobiotics by cytochrome P450 | 7 | AKR1C2, GSTM3, GSTM4, CYP2S1, GSTT2, CBR3, AKR1C1 |
| KEGG_PATHWAY | hsa04151:PI3K-Akt signaling pathway | 19 | COL4A2, IL6, FLT1, IGF1, ITGA2, ITGA3, GNG12, EPHA2, LAMB3, CCND1, LAMB2, LAMA5, ITGA7, COL1A2, COL6A1, LAMC1, THBS2, FGF2, SPP1 |
| KEGG_PATHWAY | hsa00620:Pyruvate metabolism | 5 | ME1, ALDH7A1, ME3, HAGHL, PC |
| KEGG_PATHWAY | hsa05410:Hypertrophic cardiomyopathy (HCM) | 7 | ACE, IL6, ITGA7, IGF1, ITGA2, CACNB3, ITGA3 |
| KEGG_PATHWAY | hsa00590:Arachidonic acid metabolism | 6 | AKR1C3, PTGES, ALOX15B, GPX3, CBR3, HPGDS |
| KEGG_PATHWAY | hsa04060:Cytokine-cytokine receptor interaction | 14 | TNFRSF6B, IL6, TNFRSF12A, TNFSF15, CCL8, CCL7, IL17RB, LIF, TNFRSF9, CCL22, TNFRSF11B, CCL13, CLCF1, IL1A |
| KEGG_PATHWAY | hsa00330:Arginine and proline metabolism | 5 | PYCR1, ALDH7A1, P4HA2, GATM, CKB |
| KEGG_PATHWAY | hsa00480:Glutathione metabolism | 5 | GSTM3, GSTM4, OPLAH, GPX3, GSTT2 |

**Supplemental Table 6. List of 2,559 shared genes in ALS(R)-iMGs compared to ALS(S)-iMGs according to whole-transcriptome differential gene expression (1.5-fold-change)**

| **Gene symbol** | **Transcript_id** | **Description** | **Gene symbol** | **Transcript_id** | **Description** | **Gene symbol** | **Transcript_id** | **Description** |
| --- | --- | --- | --- | --- | --- | --- | --- | --- |
| APOC1 | NM_001645 | apolipoprotein C1 | ZMYND15 | NM_001267822 | zinc finger MYND-type containing 15 | CYP27B1 | NM_000785 | cytochrome P450 family 27 subfamily B member 1 |
| ACAP3 | NM_030649 | ArfGAP with coiled-coil, ankyrin repeat and PH domains 3 | SNAR-B2 | NR_024230 | small ILF3/NF90-associated RNA B2 | DCTN6 | NM_006571 | dynactin subunit 6 |
| SLC35E2B | NM_001110781 | solute carrier family 35 member E2B | HERC4 | NM_001278186 | HECT and RLD domain containing E3 ubiquitin protein ligase 4 | TTI2 | NM_025115 | TELO2-interacting protein 2 |
| MEGF6 | NM_001409 | multiple EGF-like domains 6 | ARHGAP23 | NM_001199417 | Rho GTPase-activating protein 23 | RNF122 | NM_024787 | ring finger protein 122 |
| LINC00520 | NR_026796 | long intergenic non-protein coding RNA 520 | CIR1 | NM_004882 | corepressor interacting with RBPJ, 1 | SLC20A2 | NM_006749 | solute carrier family 20 member 2 |
| TMEM201 | NM_001010866 | transmembrane protein 201 | PPA1 | NM_021129 | pyrophosphatase (inorganic) 1 | TMEM68 | NM_001286657 | transmembrane protein 68 |
| APITD1-CORT | NM_001270517 | APITD1-CORT readthrough | PALD1 | NM_014431 | phosphatase domain containing, paladin 1 | YTHDF3-AS1 | NR_102684 | YTHDF3 antisense RNA 1 (head to head) |
| RHBDF1 | NM_022450 | rhomboid 5 homolog 1 (Drosophila) | NRCAM | NM_001193584 | neuronal cell adhesion molecule | CSF3R | NM_172313 | Colony-stimulating factor 3 receptor |
| COL6A1 | NM_001848 | collagen type VI alpha 1 | ADK | NM_001202450 | adenosine kinase | IL10 | NM_000572 | interleukin 10 |
| OVOL1 | NM_004561 | ovo like zinc finger 1 | CD9 | NM_001769 | CD9 molecule | STON2 | NM_001256430 | stonin 2 |
| ITGA3 | NM_002204 | integrin subunit alpha 3 | COL1A1 | NM_000088 | collagen type I alpha 1 | ENPP2 | NM_001130863 | ectonucleotide pyrophosphatase/phosphodiesterase 2 |
| RCC2 | NM_018715 | regulator of chromosome condensation 2 | CCSER2 | NM_018999 | coiled-coil serine rich protein 2 | PID1 | NM_017933 | phosphotyrosine-interaction domain containing 1 |
| LOC344887 | NR_033752 | NmrA-like family domain containing 1 pseudogene | LOC439994 | NR_029408 |  | TACSTD2 | NM_002353 | tumor-associated calcium signal transducer 2 |
| LOC100506730 | NR_037847 | uncharacterized LOC100506730 | AK5 | NM_174858 | adenylate kinase 5 | MROH6 | NM_001100878 | maestro heat-like repeat family member 6 |
| PIR | NM_001018109 | pirin | ZNF219 | NM_016423 | zinc finger protein 219 | SNAI1 | NM_005985 | snail family zinc finger 1 |
| TMEM151A | NM_153266 | transmembrane protein 151A | BLNK | NR_047682 | B-cell linker | BOP1 | NM_015201 | block of proliferation 1 |
| PLAU | NM_002658 | plasminogen activator, urokinase | E2F1 | NM_005225 | E2F transcription factor 1 | TMEM249 | NM_001252404 | transmembrane protein 249 |
| USP2 | NM_001243759 | Ubiquitin-specific peptidase 2 | ACOT4 | NM_152331 | acyl-CoA thioesterase 4 | FBXL6 | NM_012162 | F-box and leucine-rich repeat protein 6 |
| WWTR1 | NM_001168280 | WW domain containing transcription regulator 1 | HAGHL | NM_001290139 | hydroxyacylglutathione hydrolase-like | BST1 | NM_004334 | bone marrow stromal cell antigen 1 |
| MFSD12 | NM_174983 | major facilitator superfamily domain containing 12 | NOLC1 | NM_004741 | nucleolar and coiled-body phosphoprotein 1 | FYN | NM_153047 | FYN proto-oncogene, Src family tyrosine kinase |
| TOE1 | NM_025077 | target of EGR1, member 1 (nuclear) | ABCD1 | NM_000033 | ATP-binding cassette subfamily D member 1 | FCER2 | NM_001207019 | Fc fragment of IgE receptor II |
| FAM81A | NM_152450 | family with sequence similarity 81 member A | TMEM45A | NM_018004 | transmembrane protein 45A | CCDC171 | NM_173550 | coiled-coil domain containing 171 |
| PLIN2 | NR_038064 | perilipin 2 | LRRC6 | NM_012472 | leucine-rich repeat containing 6 | FAM118A | NM_001104595 | family with sequence similarity 118 member A |
| CCNA1 | NM_001111046 | cyclin A1 | LACTB2-AS1 | NR_038881 | LACTB2 antisense RNA 1 | ARFGEF1 | NM_006421 | ADP ribosylation factor guanine nucleotide exchange factor 1 |
| TTTY15 | NR_001545 | testis-specific transcript, Y-linked 15 (non-protein coding) | LOC103344931 | NR_120684 | uncharacterized LOC103344931 | S100P | NM_005980 | S100 calcium-binding protein P |
| PGM1 | NM_002633 | phosphoglucomutase 1 | TRUB1 | NM_139169 | TruB pseudouridine synthase family member 1 | SNCA | NM_007308 | synuclein, alpha (non A4 component of amyloid precursor) |
| MIER1 | NM_001146111 | MIER1 transcriptional regulator | INPP5F | NM_001243195 | inositol polyphosphate-5-phosphatase F | LOC403323 | NR_122076 | uncharacterized LOC403323 |
| S100A16 | NM_080388 | S100 calcium-binding protein A16 | WDR11 | NM_018117 | WD repeat domain 11 | PTAR1 | NM_001099666 | protein prenyltransferase alpha subunit repeat containing 1 |
| FNIP2 | NM_020840 | Folliculin-interacting protein 2 | FAM24B | NM_001204364 | family with sequence similarity 24 member B | ATR | NM_001184 | ATR serine/threonine kinase |
| CD274 | NM_014143 | CD274 molecule | LPP-AS2 | NR_036497 | LPP antisense RNA 2 | TC2N | NM_001128595 | tandem C2 domains, nuclear |
| MT1E | NM_175617 | metallothionein 1E | PRSS21 | NM_006799 | protease, serine 21 | KCNA3 | NR_109846 | potassium voltage-gated channel subfamily A member 3 |
| SH3D21 | NM_001162530 | SH3 domain containing 21 | PRADC1 | NM_032319 | protease-associated domain containing 1 | TGFBR3 | NM_003243 | transforming growth factor-beta receptor 3 |
| SLC35A3 | NM_012243 | solute carrier family 35 member A3 | LRRC56 | NM_198075 | leucine-rich repeat containing 56 | CENPP | NM_001286969 | centromere protein P |
| TCHH | NM_007113 | trichohyalin | LMNTD2 | NM_173573 | lamin tail domain containing 2 | IPPK | NM_022755 | inositol-pentakisphosphate 2-kinase |
| DENND2D | NM_001271833 | DENN domain containing 2D | PPP1R16A | NM_032902 | protein phosphatase 1 regulatory subunit 16A | IPW | NR_023915 | imprinted in Prader-Willi syndrome (non-protein coding) |
| SPSB1 | NM_025106 | splA/ryanodine receptor domain and SOCS box containing 1 | LILRB4 | NM_001278428 | leukocyte immunoglobulin like receptor B4 | GBP1 | NM_002053 | guanylate-binding protein 1 |
| NUDT17 | NM_001012758 | nudix hydrolase 17 | SYT17 | NM_016524 | synaptotagmin 17 | PTPDC1 | NM_177995 | protein tyrosine phosphatase domain containing 1 |
| RPP25 | NM_017793 | ribonuclease P/MRP subunit p25 | KCNQ1OT1 | NR_002728 | KCNQ1 opposite strand/antisense transcript 1 (non-protein coding) | LINC00476 | NR_023390 | long intergenic non-protein coding RNA 476 |
| CHD1L | NM_004284 | chromodomain helicase DNA-binding protein 1-like | TMEM5-AS1 | NR_126167 | TMEM5 antisense RNA 1 | ERCC6L2 | NM_001010895 | excision repair cross-complementation group 6 like 2 |
| DOCK4 | NM_014705 | dedicator of cytokinesis 4 | MT1X | NM_005952 | metallothionein 1X | CD101 | NM_001256111 | CD101 molecule |
| RPRD2 | NM_001297673 | regulation of nuclear pre-mRNA domain containing 2 | MRGPRF-AS1 | NR_120541 | MRGPRF antisense RNA 1 | CD2AP | NM_012120 | CD2-associated protein |
| TUFT1 | NM_020127 | tuftelin 1 | PIGZ | NM_025163 | phosphatidylinositol glycan anchor biosynthesis class Z | MRPL50 | NM_019051 | mitochondrial ribosomal protein L50 |
| ST5 | NM_213618 | suppression of tumorigenicity 5 | ZNF579 | NM_152600 | zinc finger protein 579 | SAMSN1 | NM_022136 | SAM domain, SH3 domain and nuclear localization signals 1 |
| FLG | NM_002016 | filaggrin | NCR3LG1 | NM_001202439 | natural killer cell cytotoxicity receptor 3 ligand 1 | SESN3 | NM_144665 | sestrin 3 |
| PACSIN3 | NM_001184974 | protein kinase C and casein kinase substrate in neurons 3 | TREX1 | NM_007248 | three prime repair exonuclease 1 | C1RL | NM_001297642 | complement C1r subcomponent like |
| ASIP | NM_001672 | agouti signaling protein | LOC100506675 | NR_120526 | uncharacterized LOC100506675 | CD200R1 | NM_138806 | CD200 receptor 1 |
| CECR2 | NM_001290046 | cat eye syndrome chromosome region, candidate 2 | TCP11L1 | NM_018393 | t-complex 11-like 1 | IRAK2 | NM_001570 | interleukin 1 receptor associated kinase 2 |
| RALA | NM_005402 | v-ral simian leukemia viral oncogene homolog A (ras related) | LOC101927768 | NR_125845 | uncharacterized LOC101927768 | RHOBTB3 | NM_014899 | Rho related BTB domain containing 3 |
| SUCO | NM_016227 | SUN domain containing ossification factor | SEC14L1P1 | NR_026952 | SEC14-like 1 pseudogene 1 | TMEFF1 | NM_003692 | transmembrane protein with EGF like and two follistatin like domains 1 |
| HILPDA | NM_001098786 | Hypoxia-inducible lipid droplet associated | GATS | NM_178831 | GATS, stromal antigen 3 opposite strand | TTC16 | NM_144965 | tetratricopeptide repeat domain 16 |
| EDEM3 | NM_025191 | ER degradation-enhancing alpha-mannosidase-like protein 3 | LRP4-AS1 | NR_038909 | LRP4 antisense RNA 1 | TOR2A | NM_001252018 | torsin family 2 member A |
| ANXA2P3 | NR_001446 | annexin A2 pseudogene 3 | SLC39A3 | NM_144564 | solute carrier family 39 member 3 | SLC25A25-AS1 | NR_033374 | SLC25A25 antisense RNA 1 |
| CDC73 | NM_024529 | cell division cycle 73 | FAM166B | NM_001099951 | family with sequence similarity 166 member B | DNM1 | NM_001005336 | dynamin 1 |
| APOC1P1 | NR_028414 | apolipoprotein C1 pseudogene 1 | ZNF818P | NR_073396 | zinc finger protein 818, pseudogene | CCBL1 | NR_109829 |  |
| ZC3H11A | NM_014827 | zinc finger CCCH-type containing 11A | DTX4 | NM_001300727 | deltex 4, E3 ubiquitin ligase | PHYHD1 | NM_174933 | phytanoyl-CoA dioxygenase domain containing 1 |
| TMCC2 | NM_014858 | transmembrane and coiled-coil domain family 2 | CCDC86 | NM_024098 | coiled-coil domain containing 86 | DOLK | NM_014908 | dolichol kinase |
| INTS7 | NM_015434 | integrator complex subunit 7 | OAS1 | NM_001032409 | 2'-5'-oligoadenylate synthetase 1 | HSPA7 | NR_024151 | heat shock protein family A (Hsp70) member 7 |
| ZNF816 | NM_001031665 | zinc finger protein 816 | ZNHIT2 | NM_014205 | zinc finger HIT-type containing 2 | LINC01503 | NR_120685 | long intergenic non-protein coding RNA 1503 |
| RRP15 | NM_016052 | ribosomal RNA processing 15 homolog | LRCH1 | NM_001164211 | leucine-rich repeats and calponin homology (CH) domain containing 1 | IL1R2 | NM_004633 | interleukin 1 receptor type 2 |
| HSPB1 | NM_001540 | heat shock protein family B (small) member 1 | RRP7BP | NR_002184 | ribosomal RNA processing 7 homolog B, pseudogene | NFATC2 | NM_001136021 | nuclear factor of activated T-cells 2 |
| RNF215 | NM_001017981 | ring finger protein 215 | STARD10 | NM_006645 | StAR related lipid transfer domain containing 10 | NCKAP1 | NM_205842 | NCK-associated protein 1 |
| RHOBTB2 | NM_001160036 | Rho-related BTB domain containing 2 | BBS1 | NM_024649 | Bardet-Biedl syndrome 1 | STK17B | NM_004226 | serine/threonine kinase 17b |
| GNPAT | NM_014236 | glyceronephosphate O-acyltransferase | RCE1 | NM_005133 | Ras-converting CAAX endopeptidase 1 | LBH | NM_030915 | limb bud and heart development |
| FABP5 | NM_001444 | fatty acid-binding protein 5 | ARMCX1 | NM_016608 | armadillo repeat containing, X-linked 1 | CST7 | NM_003650 | cystatin F |
| GGPS1 | NM_001037277 | geranylgeranyl diphosphate synthase 1 | DHRS9 | NM_199204 | dehydrogenase/reductase (SDR family) member 9 | CCL18 | NM_002988 | C-C motif chemokine ligand 18 |
| DLC1 | NM_006094 | DLC1 Rho GTPase-activating protein | SLC35G2 | NM_001097600 | solute carrier family 35 member G2 | EXD3 | NM_017820 | exonuclease 3'-5' domain containing 3 |
| H2AFY2 | NM_018649 | H2A histone family member Y2 | ACTA2-AS1 | NR_125373 | ACTA2 antisense RNA 1 | PADI2 | NM_007365 | peptidyl arginine deiminase 2 |
| CEP170 | NM_014812 | centrosomal protein 170 | SRD5A3 | NM_024592 | steroid 5 alpha-reductase 3 | GTPBP6 | NM_012227 | GTP-binding protein 6 (putative) |
| ZNF672 | NM_024836 | zinc finger protein 672 | LOC100507291 | NR_121608 | uncharacterized LOC100507291 | ASMTL | NM_001173474 | acetylserotonin O-methyltransferase-like |
| TNS2 | NM_198316 | tensin 2 | ACTA2 | NM_001141945 | actin, alpha 2, smooth muscle, aorta | ARSD | NM_001669 | arylsulfatase D |
| MMP15 | NM_002428 | matrix metallopeptidase 15 | CDH1 | NM_004360 | cadherin 1 | LOC389906 | NR_034031 | zinc finger protein 839 pseudogene |
| MAP7 | NM_003980 | microtubule-associated protein 7 | FLJ46906 | NR_033896 | uncharacterized LOC441172 | CDKN2D | NM_079421 | cyclin-dependent kinase inhibitor 2D |
| VILL | NM_015873 | villin-like | P2RY6 | NM_176797 | pyrimidinergic receptor P2Y6 | MAP3K15 | NM_001001671 | mitogen-activated protein kinase kinase kinase 15 |
| MSRB2 | NM_012228 | methionine sulfoxide reductase B2 | NPAS2 | NM_002518 | neuronal PAS domain protein 2 | CPNE5 | NM_020939 | copine 5 |
| TUBB3 | NM_001197181 | tubulin beta 3 class III | RAB30-AS1 | NR_038903 | RAB30 antisense RNA 1 (head to head) | GK | NM_000167 | glycerol kinase |
| ARHGAP12 | NM_018287 | Rho GTPase activating protein 12 | SLC35E4 | NM_001001479 | solute carrier family 35 member E4 | ANK2 | NM_001127493 | ankyrin 2, neuronal |
| ZFAND4 | NM_001282906 | zinc finger AN1-type containing 4 | MMP8 | NM_001304442 | matrix metallopeptidase 8 | FTSJ1 | NM_012280 | FtsJ RNA methyltransferase homolog 1 (E. coli) |
| LOC101928324 | NR_125953 | uncharacterized LOC101928324 | SAMD13 | NM_001010971 | sterile alpha motif domain containing 13 | USP27X | NM_001145073 | Ubiquitin-specific peptidase 27, X-linked |
| RUFY2 | NR_103475 | RUN and FYVE domain containing 2 | PTS | NM_000317 | 6-pyruvoyltetrahydropterin synthase | C2orf88 | NM_001042521 | chromosome 2 open reading frame 88 |
| KAT6B | NM_001256468 | lysine acetyltransferase 6B | TTC12 | NM_017868 | tetratricopeptide repeat domain 12 | IGIP | NM_001007189 | IgA-inducing protein |
| EIF2AK3 | NM_004836 | eukaryotic translation initiation factor 2 alpha kinase 3 | NAALADL1 | NM_005468 | N-acetylated alpha-linked acidic dipeptidase-like 1 | ARHGEF9 | NM_001173479 | Cdc42 guanine nucleotide exchange factor 9 |
| ST6GALNAC4 | NM_175039 | ST6 N-acetylgalactosaminide alpha-2,6-sialyltransferase 4 | CBY1 | NM_015373 | chibby homolog 1 (Drosophila) | AQP3 | NM_004925 | aquaporin 3 (Gill blood group) |
| C1QTNF9B-AS1 | NM_001014442 | C1QTNF9B antisense RNA 1 | HMBS | NM_001258208 | hydroxymethylbilane synthase | SORL1 | NM_003105 | sortilin-related receptor, L(DLR class) A repeats containing |
| ZNF618 | NM_133374 | zinc finger protein 618 | TRAPPC2L | NM_016209 | trafficking protein particle complex 2-like | TAF1 | NM_138923 | TATA-box binding protein-associated factor 1 |
| GPR137B | NM_003272 | G protein-coupled receptor 137B | LOC341056 | NR_027288 | SUMO1-activating enzyme subunit 1 pseudogene | HDX | NM_144657 | highly divergent homeobox |
| BEAN1 | NM_001197224 | brain expressed, associated with NEDD4, 1 | VWA5A | NM_014622 | von Willebrand factor A domain containing 5A | BEX5 | NM_001159560 | brain expressed X-linked 5 |
| SPNS1 | NM_001142448 | spinster homolog 1 (Drosophila) | C16orf46 | NM_001100873 | chromosome 16 open reading frame 46 | VLDLR | NM_001018056 | very-low-density lipoprotein receptor |
| TRPA1 | NM_007332 | transient receptor potential cation channel subfamily A member 1 | LOC100049716 | NR_122124 | uncharacterized LOC100049716 | CD93 | NM_012072 | CD93 molecule |
| KIF17 | NM_020816 | kinesin family member 17 | BATF3 | NM_018664 | basic leucine zipper ATF-like transcription factor 3 | ACSL4 | NM_022977 | acyl-CoA synthetase long-chain family member 4 |
| COL9A2 | NM_001852 | collagen type IX alpha 2 | TSTA3 | NM_003313 | tissue-specific transplantation antigen P35B | ZBTB33 | NM_006777 | zinc finger and BTB domain containing 33 |
| TAF5 | NM_006951 | TATA-box binding protein-associated factor 5 | PCDH1 | NM_032420 | protocadherin 1 | GTPBP6 | NM_012227_1 | GTP-binding protein 6 (putative) |
| TTC28 | NM_001145418 | tetratricopeptide repeat domain 28 | TBC1D7 | NM_016495 | TBC1 domain family member 7 | RGS18 | NM_130782 | regulator of G-protein signaling 18 |
| ZDHHC6 | NM_001303134 | zinc finger DHHC-type containing 6 | ITGA2 | NM_002203 | integrin subunit alpha 2 | MMP12 | NM_002426 | matrix metallopeptidase 12 |
| DHX32 | NM_018180 | DEAH-box helicase 32 (putative) | ATP9A | NM_006045 | ATPase phospholipid transporting 9A (putative) | RAI14 | NM_015577 | retinoic acid induced 14 |
| LRP12 | NM_001135703 | low density lipoprotein receptor-related protein 12 | MRPL48 | NM_016055 | mitochondrial ribosomal protein L48 | BAALC | NM_001024372 | brain and acute leukemia, cytoplasmic |
| ERI3 | NM_001301698 | ERI1 exoribonuclease family member 3 | FAM86FP | NR_024254 | family with sequence similarity 86 member F, pseudogene | BCAR1 | NM_001170717 | BCAR1, Cas family scaffolding protein |
| TMEM80 | NM_174940 | transmembrane protein 80 | PTER | NM_030664 | phosphotriesterase related | RHCG | NR_110261 | Rh family C glycoprotein |
| PIDD1 | NM_145886 | p53-induced death domain protein 1 | C19orf73 | NM_018111 | chromosome 19 open reading frame 73 | CLIC6 | NM_053277 | chloride intracellular channel 6 |
| POLR2L | NM_021128 | polymerase (RNA) II subunit L | C9orf116 | NM_144654 | chromosome 9 open reading frame 116 | DDIT4L | NM_145244 | DNA damage-inducible transcript 4-like |
| UNC79 | NM_020818 | unc-79 homolog (C. elegans) | SYNGR1 | NM_145731 | synaptogyrin 1 | S1PR1 | NM_001400 | sphingosine-1-phosphate receptor 1 |
| DUSP8 | NM_004420 | dual-specificity phosphatase 8 | BAG3 | NM_004281 | BCL2-associated athanogene 3 | CSRP2 | NM_001321 | cysteine and glycine rich protein 2 |
| GPR85 | NM_001146266 | G protein-coupled receptor 85 | LMAN2L | NR_024518 | lectin, mannose-binding 2-like | SMAD3 | NM_005902 | SMAD family member 3 |
| FAR1 | NM_032228 | fatty acyl-CoA reductase 1 | C2CD5 | NM_001286177 | C2 calcium-dependent domain containing 5 | ITGBL1 | NM_004791 | integrin subunit beta like 1 |
| AKR7A2 | NM_003689 | aldo-keto reductase family 7, member A2 | LMLN | NR_026787 | Leishmanolysin-like peptidase | STAC2 | NM_198993 | SH3 and cysteine rich domain 2 |
| CYP2R1 | NM_024514 | cytochrome P450 family 2 subfamily R member 1 | RUSC1 | NM_001105204 | RUN and SH3 domain containing 1 | MRGPRF | NM_001098515 | MAS-related GPR family member F |
| PCK2 | NM_001291556 | phosphoenolpyruvate carboxykinase 2, mitochondrial | ASUN | NM_018164 | asunder, spermatogenesis regulator | HTRA3 | NM_053044 | HtrA serine peptidase 3 |
| TNFAIP6 | NM_007115 | TNF alpha-induced protein 6 | SNORD8 | NR_002916 | small nucleolar RNA, C/D box 8 | LINC01050 | NR_125747 | long intergenic non-protein coding RNA 1050 |
| TTC17 | NM_001307943 | tetratricopeptide repeat domain 17 | KIF21A | NM_017641 | kinesin family member 21A | SHOX2 | NM_001163678 | short stature homeobox 2 |
| MADD | NM_130470 | MAP kinase-activating death domain | SLC2A13 | NM_052885 | solute carrier family 2 member 13 | PCOLCE2 | NM_013363 | procollagen C-endopeptidase enhancer 2 |
| LPXN | NM_001143995 | leupaxin | ST6GALNAC2 | NM_006456 | ST6 N-acetylgalactosaminide alpha-2,6-sialyltransferase 2 | ZNF436-AS1 | NR_033690 | ZNF436 antisense RNA 1 |
| TMEM216 | NM_001173990 | transmembrane protein 216 | PTX3 | NM_002852 | pentraxin 3 | REEP1 | NM_001164730 | receptor accessory protein 1 |
| SPRY1 | NM_199327 | sprouty RTK signaling antagonist 1 | PFKM | NM_001166686 | phosphofructokinase, muscle | TM4SF1 | NM_014220 | transmembrane 4 L six family member 1 |
| POLR2G | NM_002696 | polymerase (RNA) II subunit G | RHEBL1 | NM_144593 | Ras homolog enriched in brain like 1 | ARHGAP6 | NR_109776 | Rho GTPase activating protein 6 |
| PLXNA3 | NM_017514 | plexin A3 | PRPF40B | NM_001031698 | pre-mRNA processing factor 40 homolog B | RABGGTB | NM_004582 | Rab geranylgeranyltransferase beta subunit |
| CATSPER1 | NM_053054 | cation channel sperm associated 1 | LOC100287015 | NR_040040 | uncharacterized LOC100287015 | ST6GALNAC3 | NM_152996 | ST6 N-acetylgalactosaminide alpha-2,6-sialyltransferase 3 |
| DPP3 | NM_001256670 | dipeptidyl peptidase 3 | TMEM163 | NM_030923 | transmembrane protein 163 | FNBP1L | NM_017737 | formin-binding protein 1-like |
| DOCK7 | NM_001272000 | dedicator of cytokinesis 7 | TNFRSF10A | NM_003844 | tumor necrosis factor receptor superfamily member 10a | CTH | NM_001902 | cystathionine gamma-lyase |
| CDC42BPA | NM_003607 | CDC42-binding protein kinase alpha | NHLRC1 | NM_198586 | NHL repeat containing E3 ubiquitin protein ligase 1 | PEG3 | NM_001146185 | paternally expressed 3 |
| AP2S1 | NM_001301076 | adaptor -elated protein complex 2 sigma 1 subunit | NCDN | NM_001014839 | neurochondrin | FAM212B | NM_019099 | family with sequence similarity 212 member B |
| EOGT | NM_001278689 | EGF domain-specific O-linked N-acetylglucosamine transferase | DYRK4 | NM_003845 | dual specificity tyrosine phosphorylation regulated kinase 4 | ALDH1A2 | NM_170696 | aldehyde dehydrogenase 1 family member A2 |
| S100A2 | NM_005978 | S100 calcium-binding protein A2 | TSPAN31 | NM_005981 | tetraspanin 31 | PALLD | NM_016081 | palladin, cytoskeletal associated protein |
| USP12 | NM_182488 | Ubiquitin-specific peptidase 12 | ZG16B | NM_145252 | zymogen granule protein 16B | PROC | NM_000312 | protein C, inactivator of coagulation factors Va and VIIIa |
| PBX3 | NM_006195 | pre-B-cell leukemia homeobox 3 | TSFM | NM_001172696 | Ts translation elongation factor, mitochondrial | SERPINE2 | NM_001136528 | serpin family E member 2 |
| MRE11A | NM_005590 | MRE11 homolog A, double strand break repair nuclease | PKIB | NM_181794 | protein kinase (cAMP-dependent, catalytic) inhibitor beta | IL17RC | NM_032732 | interleukin 17 receptor C |
| MSANTD4 | NM_032424 | Myb/SANT DNA-binding domain containing 4 with coiled-coils | C12orf66 | NM_001300940 | chromosome 12 open reading frame 66 | SPRR3 | NM_005416 | small proline-rich protein 3 |
| SRGAP1 | NM_020762 | SLIT-ROBO Rho GTPase activating protein 1 | PLXNA2 | NM_025179 | plexin A2 | SPRR2D | NM_006945 | small proline-rich protein 2D |
| DCBLD1 | NM_173674 | discoidin, CUB and LCCL domain containing 1 | IRAK3 | NM_007199 | interleukin 1 receptor associated kinase 3 | HSPG2 | NM_005529 | heparan sulfate proteoglycan 2 |
| SLC25A23 | NM_024103 | solute carrier family 25 member 23 | SNRNP25 | NM_024571 | small nuclear ribonucleoprotein U11/U12 subunit 25 | TMEM173 | NM_198282 | transmembrane protein 173 |
| TRPM4 | NM_017636 | transient receptor potential cation channel subfamily M member 4 | BCL7C | NM_001286526 | B-cell CLL/lymphoma 7C | MACC1 | NM_182762 | metastasis associated in colon cancer 1 |
| CCDC24 | NM_152499 | coiled-coil domain containing 24 | THNSL2 | NM_001244678 | threonine synthase-like 2 | GPRC5A | NM_003979 | G protein-coupled receptor class C group 5 member A |
| TMEM136 | NM_001198674 | transmembrane protein 136 | LINC01481 | NR_120460 | long intergenic non-protein coding RNA 1481 | MDK | NM_001270550 | midkine (neurite growth-promoting factor 2) |
| ZNF202 | NM_003455 | zinc finger protein 202 | RPL23AP53 | NR_003572 | ribosomal protein L23a pseudogene 53 | ARL4D | NM_001661 | ADP ribosylation factor like GTPase 4D |
| HACD1 | NM_014241 | 3-hydroxyacyl-CoA dehydratase 1 | A1BG-AS1 | NR_015380 | A1BG antisense RNA 1 | LDLRAD2 | NM_001013693 | low-density lipoprotein receptor class A domain containing 2 |
| USP53 | NM_019050 | Ubiquitin-specific peptidase 53 | COL4A1 | NM_001845 | collagen type IV alpha 1 | MAP3K14 | NM_003954 | mitogen-activated protein kinase kinase kinase 14 |
| P2RY1 | NM_002563 | purinergic receptor P2Y1 | PLEKHG7 | NM_001004330 | pleckstrin homology and RhoGEF domain containing G7 | S100A13 | NM_005979 | S100 calcium-binding protein A13 |
| DKK2 | NM_014421 | dickkopf WNT signaling pathway inhibitor 2 | EEA1 | NM_003566 | early endosome antigen 1 | LAMB3 | NM_001017402 | laminin subunit beta 3 |
| ST14 | NM_021978 | suppression of tumorigenicity 14 | NR2C1 | NM_003297 | nuclear receptor subfamily 2 group C member 1 | LY86 | NM_004271 | lymphocyte antigen 86 |
| ZNF584 | NM_173548 | zinc finger protein 584 | CDK17 | NM_002595 | cyclin-dependent kinase 17 | CCL13 | NM_005408 | C-C motif chemokine ligand 13 |
| CD27-AS1 | NR_015382 | CD27 antisense RNA 1 | SLC39A14 | NM_015359 | solute carrier family 39 member 14 | KIAA1804 | NM_032435 | mixed lineage kinase 4 |
| NCAPD2 | NM_014865 | non-SMC condensin I complex subunit D2 | SCYL2 | NM_017988 | SCY1 like pseudokinase 2 | SPINK6 | NM_001195290 | serine peptidase inhibitor, Kazal type 6 |
| ZNF384 | NM_001039920 | zinc finger protein 384 | WDR19 | NM_025132 | WD repeat domain 19 | MYOZ1 | NM_021245 | myozenin 1 |
| COPS7A | NM_016319 | COP9 signalosome subunit 7A | EFCAB2 | NM_032328 | EF-hand calcium-binding domain 2 | TEKT4P2 | NR_038328 | tektin 4 pseudogene 2 |
| C4orf48 | NM_001168243 | chromosome 4 open reading frame 48 | MCM5 | NM_006739 | minichromosome maintenance complex component 5 | ZWINT | NM_007057 | ZW10-interacting kinetochore protein |
| MSANTD3-TMEFF1 | NM_001198812 | MSANTD3-TMEFF1 readthrough | LINC00173 | NR_027345 | long intergenic non-protein coding RNA 173 | NMB | NM_205858 | neuromedin B |
| DDX47 | NM_016355 | DEAD-box helicase 47 | C12orf43 | NM_022895 | chromosome 12 open reading frame 43 | POLR2J4 | NR_003655 | polymerase (RNA) II subunit J4, pseudogene |
| KIAA1467 | NM_020853 |  | BCL7A | NM_001024808 | B-cell CLL/lymphoma 7A | TNKS2 | NM_025235 | tankyrase 2 |
| FGFR1OP2 | NM_001171887 | FGFR1 oncogene partner 2 | VPS37B | NM_024667 | VPS37B, ESCRT-I subunit | CCDC78 | NM_001031737 | coiled-coil domain containing 78 |
| BICD1 | NM_001003398 | BICD cargo adaptor 1 | TAMM41 | NM_001284401 | TAM41 mitochondrial translocator assembly and maintenance homolog | NHLRC2 | NM_198514 | NHL repeat containing 2 |
| SNORA34 | NR_002968 |  | FAM109B | NM_001002034 | family with sequence similarity 109 member B | ACADSB | NM_001609 | acyl-CoA dehydrogenase, short/branched chain |
| CDK14 | NM_001287135 | cyclin-dependent kinase 14 | GREB1 | NM_014668 | growth regulation by estrogen in breast cancer 1 | STARD13 | NM_052851 | StAR-related lipid transfer domain containing 13 |
| ITGA7 | NM_001144996 | integrin subunit alpha 7 | ZDHHC20 | NR_104487 | zinc finger DHHC-type containing 20 | ASB2 | NM_016150 | ankyrin repeat and SOCS box containing 2 |
| C11orf71 | NM_019021 | chromosome 11 open reading frame 71 | RNF6 | NM_183043 | ring finger protein 6 | LOC728084 | NR_038385 | uncharacterized LOC728084 |
| MON2 | NM_001278471 | MON2 homolog, regulator of endosome-to-Golgi trafficking | GPATCH2 | NM_018040 | G-patch domain containing 2 | DNAJC5B | NM_033105 | DnaJ heat shock protein family (Hsp40) member C5 beta |
| LEMD3 | NM_001167614 | LEM domain containing 3 | CAMK2B | NM_001293170 | calcium/calmodulin dependent protein kinase II beta | NUDT12 | NM_001300741 | nudix hydrolase 12 |
| PABPC4 | NM_003819 | poly(A)-binding protein cytoplasmic 4 | DNAJC15 | NM_013238 | DnaJ heat shock protein family (Hsp40) member C15 | LIAS | NM_006859 | lipoic acid synthetase |
| ZDHHC17 | NM_015336 | zinc finger DHHC-type containing 17 | GPALPP1 | NM_018559 | GPALPP motifs containing 1 | ANKRD42 | NR_125355 | ankyrin repeat domain 42 |
| SIGMAR1 | NM_001282205 | sigma non-opioid intracellular receptor 1 | DHRS12 | NM_001270424 | dehydrogenase/reductase (SDR family) member 12 | IL1RN | NM_173843 | interleukin 1 receptor antagonist |
| NFYB | NM_006166 | nuclear transcription factor Y subunit beta | INSIG2 | NM_016133 | Insulin-induced gene 2 | CTSV | NM_001333 | cathepsin V |
| KIAA1033 | NM_001293640 | KIAA1033 | MZT1 | NM_001071775 | mitotic spindle organizing protein 1 | PUS3 | NM_031307 | pseudouridylate synthase 3 |
| GPN3 | NM_016301 | GPN-loop GTPase 3 | PIBF1 | NM_006346 | progesterone immunomodulatory-binding factor 1 | MVB12B | NM_033446 | multivesicular body subunit 12B |
| FAM3C | NM_014888 | family with sequence similarity 3 member C | ZNF234 | NM_001144824 | zinc finger protein 234 | SCG5 | NM_001144757 | secretogranin V |
| CAMKK2 | NM_001270486 | calcium/calmodulin-dependent protein kinase kinase 2 | RNF219 | NM_024546 | ring finger protein 219 | DYNC2LI1 | NM_015522 | dynein cytoplasmic 2 light intermediate chain 1 |
| C12orf65 | NM_152269 | chromosome 12 open reading frame 65 | TGDS | NR_130731 | TDP-glucose 4,6-dehydratase | FABP4 | NM_001442 | fatty acid-binding protein 4 |
| KLHDC10 | NM_014997 | kelch domain containing 10 | TFPI | NM_006287 | tissue factor pathway inhibitor | PROCR | NM_006404 | protein C receptor |
| ZMYM2 | NM_197968 | zinc finger MYM-type containing 2 | NEFH | NM_021076 | neurofilament, heavy polypeptide | CLLU1OS | NM_001025232 | chronic lymphocytic leukemia up-regulated 1 opposite strand |
| CEBPA | NM_001287435 | CCAAT/enhancer-binding protein alpha | TRMT12 | NM_017956 | tRNA methyltransferase 12 homolog (S. cerevisiae) | CMKLR1 | NM_001142345 | chemerin chemokine-like receptor 1 |
| LNX2 | NM_153371 | ligand of numb-protein X 2 | KAT8 | NM_182958 | lysine acetyltransferase 8 | JAKMIP2 | NM_001270934 | janus kinase and microtubule-interacting protein 2 |
| IFT122 | NM_001280541 | intraflagellar transport 122 | LOC101927438 | NR_110224 | uncharacterized LOC101927438 | CCDC64 | NM_207311 |  |
| RFC3 | NM_181558 | replication factor C subunit 3 | HDHD3 | NM_001304510 | haloacid dehalogenase-like hydrolase domain containing 3 | SEC14L2 | NM_033382 | SEC14-like lipid-binding 2 |
| ACADS | NM_001302554 | acyl-CoA dehydrogenase, C-2 to C-3 short chain | DHRS4 | NM_001282988 | dehydrogenase/reductase (SDR family) member 4 | CENPJ | NM_018451 | centromere protein J |
| HHLA3 | NM_001036646 | HERV-H LTR-associating 3 | LTB4R2 | NM_019839 | leukotriene B4 receptor 2 | ZNF761 | NM_001289951 | zinc finger protein 761 |
| WBP4 | NM_007187 | WW domain-binding protein 4 | SCFD1 | NM_182835 | sec1 family domain containing 1 | SEMA3A | NM_006080 | semaphorin 3A |
| TBL3 | NM_006453 | transducin beta like 3 | BRMS1L | NM_032352 | breast cancer metastasis-suppressor 1-like | GAS6 | NM_000820 | growth arrest-specific 6 |
| TMEM147 | NM_032635 | transmembrane protein 147 | C14orf28 | NM_001017923 | chromosome 14 open reading frame 28 | MPEG1 | NM_001039396 | macrophage expressed 1 |
| METTL7B | NM_152637 | methyltransferase like 7B | MMP19 | NM_001272101 | matrix metallopeptidase 19 | DNAAF2 | NM_018139 | dynein (axonemal) assembly factor 2 |
| TULP3 | NM_001160408 | tubby like protein 3 | IGF2R | NM_000876 | insulin-like growth factor 2 receptor | LILRA2 | NM_006866 | leukocyte immunoglobulin like receptor A2 |
| SETDB2 | NM_031915 | SET domain bifurcated 2 | FAM173A | NM_023933 | family with sequence similarity 173 member A | WTIP | NM_001080436 | Wilms tumor 1-interacting protein |
| STYXL1 | NM_016086 | serine/threonine/tyrosine-interacting-like 1 | NUDT8 | NM_181843 | nudix hydrolase 8 | SBF2-AS1 | NR_036485 | SBF2 antisense RNA 1 |
| HIST1H2BK | NM_080593 | histone cluster 1, H2bk | CAB39L | NM_001287338 | calcium-binding protein 39-like | SERINC4 | NM_001258032 | serine incorporator 4 |
| MTUS1 | NM_001001931 | Microtubule-associated tumor suppressor 1 | STYX | NM_001130701 | serine/threonine/tyrosine-interacting protein | CCNF | NM_001761 | cyclin F |
| TRAPPC1 | NM_001166621 | trafficking protein particle complex 1 | MSL3 | NM_078628 | male-specific lethal 3 homolog (Drosophila) | ZSCAN32 | NM_017810 | zinc finger and SCAN domain containing 32 |
| SGTB | NM_019072 | small glutamine rich tetratricopeptide repeat containing beta | EXOC5 | NM_006544 | exocyst complex component 5 | DCUN1D3 | NM_173475 | defective in cullin neddylation 1 domain containing 3 |
| TECR | NR_038104 | trans-2,3-enoyl-CoA reductase | PCNXL4 | NM_022495 |  | CDR2 | NM_001802 | cerebellar degeneration related protein 2 |
| PDZD11 | NM_016484 | PDZ domain containing 11 | IGHMBP2 | NM_002180 | immunoglobulin mu-binding protein 2 | PRSS36 | NM_173502 | protease, serine 36 |
| CHAMP1 | NM_032436 | chromosome alignment maintaining phosphoprotein 1 | FAM136A | NM_032822 | family with sequence similarity 136 member A | TMEM121 | NM_025268 | transmembrane protein 121 |
| SRRD | NM_001013694 | SRR1 domain containing | MTHFD1 | NM_005956 | methylenetetrahydrofolate dehydrogenase, cyclohydrolase and formyltetrahydrofolate synthetase 1 | FXYD2 | NM_021603 | FXYD domain containing ion transport regulator 2 |
| RECQL4 | NM_004260 | RecQ like helicase 4 | PLEKHH1 | NM_020715 | pleckstrin homology, MyTH4 and FERM domain containing H1 | TANC2 | NM_025185 | tetratricopeptide repeat, ankyrin repeat and coiled-coil containing 2 |
| NDRG2 | NM_201541 | NDRG family member 2 | PIGH | NM_004569 | phosphatidylinositol glycan anchor biosynthesis class H | PDIA5 | NM_006810 | protein disulfide isomerase family A member 5 |
| OSBPL6 | NM_001201480 | Oxysterol-binding protein-like 6 | SLC5A6 | NR_028323 | solute carrier family 5 member 6 | ZNF232 | NM_014519 | zinc finger protein 232 |
| TIMM50 | NM_001001563 | translocase of inner mitochondrial membrane 50 | TSPAN5 | NM_005723 | tetraspanin 5 | ETNK2 | NM_001297761 | ethanolamine kinase 2 |
| C11orf16 | NM_020643 | chromosome 11 open reading frame 16 | RPL19P12 | NR_026660 | ribosomal protein L19 pseudogene 12 | CENPV | NM_181716 | centromere protein V |
| RBM23 | NM_001077351 | RNA-binding motif protein 23 | CRY2 | NM_001127457 | cryptochrome circadian clock 2 | FXYD6-FXYD2 | NM_001243598 | FXYD6-FXYD2 readthrough |
| SCAND2P | NR_004859 | SCAN domain containing 2 pseudogene | RAB6C | NM_032144 | RAB6C, member RAS oncogene family | RNFT1 | NM_016125 | ring finger protein, transmembrane 1 |
| RTN2 | NM_005619 | reticulon 2 | GPR137 | NM_001170726 | G protein-coupled receptor 137 | DHCR7 | NM_001163817 | 7-dehydrocholesterol reductase |
| MUT | NM_000255 | methylmalonyl-CoA mutase | SMIM13 | NM_001135575 | small integral membrane protein 13 | TK1 | NM_003258 | thymidine kinase 1 |
| INCENP | NM_020238 | inner centromere protein | IFT43 | NR_045664 | intraflagellar transport 43 | SERPINB13 | NM_012397 | serpin family B member 13 |
| ISG15 | NM_005101 | ISG15 ubiquitin-like modifier | ABCB9 | NM_001243014 | ATP-binding cassette subfamily B member 9 | GNG7 | NM_052847 | G protein subunit gamma 7 |
| G2E3 | NM_001308097 | G2/M-phase specific E3 ubiquitin protein ligase | DYNLT3 | NM_006520 | dynein light-chain Tctex-type 3 | PEX11G | NM_001270539 | peroxisomal biogenesis factor 11 gamma |
| ARHGAP5 | NM_001030055 | Rho GTPase activating protein 5 | DDO | NM_003649 | D-aspartate oxidase | ZNF317 | NR_102435 | zinc finger protein 317 |
| CTAGE5 | NM_203354 | CTAGE family member 5 | ZNF497 | NM_198458 | zinc finger protein 497 | ZNF136 | NM_003437 | zinc finger protein 136 |
| KLHL28 | NM_001308112 | kelch like family member 28 | C9orf16 | NM_024112 | chromosome 9 open reading frame 16 | ZNF566 | NM_001145345 | zinc finger protein 566 |
| GNPNAT1 | NM_198066 | glucosamine-phosphate N-acetyltransferase 1 | DCP1B | NM_152640 | decapping mRNA 1B | FCGBP | NM_003890 | Fc fragment of IgG-binding protein |
| DDHD1 | NM_030637 | DDHD domain containing 1 | MRPL28 | NM_006428 | mitochondrial ribosomal protein L28 | ZNF28 | NR_036599 | zinc finger protein 28 |
| CFD | NM_001928 | complement factor D (adipsin) | PPP1R13B | NM_015316 | protein phosphatase 1 regulatory subunit 13B | ID2-AS1 | NR_110154 | ID2 antisense RNA 1 (head to head) |
| C7orf25 | NM_024054 | chromosome 7 open reading frame 25 | BANF1 | NM_001143985 | barrier to autointegration factor 1 | ICA1L | NM_001288622 | islet cell autoantigen 1-like |
| FZR1 | NM_016263 | fizzy/cell division cycle 20 related 1 | LOC646214 | NR_027053 | p21 protein (Cdc42/Rac)-activated kinase 2 pseudogene | ZSCAN25 | NM_145115 | zinc finger and SCAN domain containing 25 |
| JKAMP | NM_016475 | JNK1/MAPK8-associated membrane protein | ZBED3 | NM_032367 | zinc finger BED-type containing 3 | PER2 | NM_022817 | period circadian clock 2 |
| DPH5 | NM_001077395 | diphthamide biosynthesis 5 | TUBGCP5 | NM_052903 | tubulin gamma complex-associated protein 5 | MORC4 | NM_001085354 | MORC family CW-type zinc finger 4 |
| TSPAN17 | NM_001006616 | tetraspanin 17 | NIPA1 | NM_144599 | non imprinted in Prader-Willi/Angelman syndrome 1 | SMARCAL1 | NM_014140 | SWI/SNF related, matrix associated, actin dependent regulator of chromatin, subfamily a-like 1 |
| MRPL4 | NM_146387 | mitochondrial ribosomal protein L4 | TMEM106B | NM_001134232 | transmembrane protein 106B | C1QC | NM_172369 | complement component 1, q subcomponent, C chain |
| LCMT1 | NM_016309 | leucine carboxyl methyltransferase 1 | TBC1D17 | NM_001168222 | TBC1 domain family member 17 | EHBP1 | NM_001142615 | EH domain-binding protein 1 |
| DNAL1 | NM_031427 | dynein axonemal light-chain 1 | ZSWIM1 | NM_080603 | zinc finger SWIM-type containing 1 | C21orf58 | NM_001286462 | chromosome 21 open reading frame 58 |
| CXorf56 | NM_001170570 | chromosome X open reading frame 56 | ABHD15 | NM_198147 | abhydrolase domain containing 15 | TRIM32 | NM_012210 | tripartite motif containing 32 |
| ISCA2 | NM_001272007 | iron-sulfur cluster assembly 2 | INO80 | NM_017553 | INO80 complex subunit | MGAT3 | NM_002409 | mannosyl (beta-1,4-)-glycoprotein beta-1,4-N-acetylglucosaminyltransferase |
| MLH3 | NM_014381 | mutL homolog 3 | ZNF668 | NM_001172670 | zinc finger protein 668 | ATAD5 | NM_024857 | ATPase family, AAA domain containing 5 |
| TUBA1B | NM_006082 | tubulin alpha 1b | PPIP5K1 | NM_014659 | diphosphoinositol pentakisphosphate kinase 1 | DENND6B | NM_001001794 | DENN domain containing 6B |
| SLC25A43 | NM_145305 | solute carrier family 25 member 43 | AP5S1 | NM_001204447 | adaptor-related protein complex 5 sigma 1 subunit | RRN3P1 | NR_003370 | RRN3 homolog, RNA polymerase I transcription factor pseudogene 1 |
| GOLGA7B | NM_001010917 | golgin A7 family member B | SPG11 | NM_025137 | spastic paraplegia 11 (autosomal recessive) | ABHD14A | NM_015407 | abhydrolase domain containing 14A |
| ATG2B | NM_018036 | autophagy-related 2B | CDK1 | NM_001170406 | cyclin-dependent kinase 1 | MTRNR2L3 | NM_001190472 | MT-RNR2-like 3 |
| NDUFA12 | NM_001258338 | NADH:ubiquinone oxidoreductase subunit A12 | PPT2 | NM_138717_3 | palmitoyl-protein thioesterase 2 | VPS13B | NM_181661 | vacuolar protein sorting 13 homolog B (yeast) |
| TNFAIP2 | NM_006291 | TNF alpha-induced protein 2 | COPS2 | NM_004236 | COP9 signalosome subunit 2 | ADORA3 | NM_000677 | adenosine A3 receptor |
| SLCO3A1 | NM_013272 | solute carrier organic anion transporter family member 3A1 | CXXC1 | NM_014593 | CXXC finger protein 1 | LOC101927272 | NR_110908 | uncharacterized LOC101927272 |
| CAMSAP2 | NM_203459 | calmodulin regulated spectrin-associated protein family member 2 | MKL2 | NM_014048 | MKL1/myocardin-like 2 | JAK3 | NM_000215 | Janus kinase 3 |
| BSCL2 | NM_032667 | Berardinelli-Seip congenital lipodystrophy 2 (seipin) | PCYOX1L | NM_024028 | prenylcysteine oxidase 1-like | LINC00969 | NR_122105 | long intergenic non-protein coding RNA 969 |
| NRSN2-AS1 | NR_109990 | NRSN2 antisense RNA 1 | TRIM47 | NM_033452 | tripartite motif containing 47 | SASS6 | NM_001304829 | SAS-6 centriolar assembly protein |
| FAM98B | NM_173611 | family with sequence similarity 98 member B | AQP9 | NM_020980 | aquaporin 9 | MCOLN2 | NM_153259 | mucolipin 2 |
| C15orf57 | NM_001289132 | chromosome 15 open reading frame 57 | BTG3 | NM_006806 | BTG family member 3 | OPRL1 | NM_182647 | Opioid-related nociceptin receptor 1 |
| MAD2L2 | NM_001127325 | MAD2 mitotic arrest deficient-like 2 (yeast) | TMEM222 | NR_037577 | transmembrane protein 222 | SCRN1 | NM_001145513 | secernin 1 |
| EIF3J-AS1 | NR_034171 | EIF3J antisense RNA 1 (head to head) | CORO7-PAM16 | NM_001201479 | CORO7-PAM16 readthrough | ELOVL6 | NM_024090 | ELOVL fatty acid elongase 6 |
| FGD6 | NM_018351 | FYVE, RhoGEF and PH domain containing 6 | C8orf33 | NM_023080 | chromosome 8 open reading frame 33 | ANOS1 | NM_000216 | anosmin 1 |
| FAAP100 | NR_033338 | Fanconi anemia core complex-associated protein 100 | FANCE | NM_021922 | Fanconi anemia complementation group E | FRMD3 | NM_001244962 | FERM domain containing 3 |
| ARRDC1 | NM_152285 | arrestin domain containing 1 | PKD2 | NM_000297 | polycystin 2, transient receptor potential cation channel | NEK1 | NM_001199397 | NIMA-related kinase 1 |
| AP4E1 | NM_001252127 | adaptor related protein complex 4 epsilon 1 subunit | UBL7 | NM_201265 | ubiquitin-like 7 | RTN4RL2 | NM_178570 | reticulon 4 receptor-like 2 |
| DMXL2 | NM_015263 | Dmx like 2 | ARSK | NM_198150 | arylsulfatase family member K | TMEM161B | NM_001289007 | transmembrane protein 161B |
| RSL24D1 | NM_016304 | ribosomal L24 domain containing 1 | ARC | NM_015193 | activity-regulated cytoskeleton-associated protein | LYSMD3 | NM_001286812 | LysM domain containing 3 |
| TMEM9 | NM_001288566 | transmembrane protein 9 | STX18 | NM_016930 | syntaxin 18 | CXCL14 | NM_004887 | C-X-C motif chemokine ligand 14 |
| CST3 | NM_001288614 | cystatin C | SCOC | NM_032547 | short coiled-coil protein | MAP3K8 | NM_001244134 | mitogen-activated protein kinase kinase kinase 8 |
| ARRDC1-AS1 | NR_122035 | ARRDC1 antisense RNA 1 | ABHD2 | NM_007011 | abhydrolase domain containing 2 | HENMT1 | NM_001102592 | HEN1 methyltransferase homolog 1 |
| OAZ2 | NM_001301302 | ornithine decarboxylase antizyme 2 | CHST7 | NM_019886 | carbohydrate sulfotransferase 7 | HLA-DOA | NM_002119_1 | major histocompatibility complex, class II, DO alpha |
| PIF1 | NM_001286497 | PIF1 5'-to-3' DNA helicase | DCAF4 | NM_181340 | DDB1 and CUL4-associated factor 4 | CCL2 | NM_002982 | C-C motif chemokine ligand 2 |
| PARP16 | NM_017851 | poly(ADP-ribose) polymerase family member 16 | CAPN15 | NM_005632 | calpain 15 | HERC6 | NM_001165136 | HECT and RLD domain containing E3 ubiquitin protein ligase family member 6 |
| IQCH-AS1 | NR_040051 | IQCH antisense RNA 1 | LINC01314 | NR_120317 | long intergenic non-protein coding RNA 1314 | MARCO | NM_006770 | macrophage receptor with collagenous structure |
| UACA | NM_018003 | uveal autoantigen with coiled-coil domains and ankyrin repeats | MAD1L1 | NM_003550 | MAD1 mitotic arrest deficient like 1 | IFFO2 | NM_001136265 | intermediate filament family orphan 2 |
| ANAPC7 | NM_016238 | Anaphase-promoting complex subunit 7 | FBXL16 | NM_153350 | F-box and leucine-rich repeat protein 16 | SEPT7P2 | NR_024271 | septin 7 pseudogene 2 |
| SPPL2A | NM_032802 | signal peptide peptidase like 2A | PELO | NM_015946 | pelota homolog (Drosophila) | MAST4 | NM_198828 | microtubule associated serine/threonine kinase family member 4 |
| ANXA11 | NM_145869 | annexin A11 | ZNF200 | NM_001145447 | zinc finger protein 200 | AMDHD1 | NM_152435 | amidohydrolase domain containing 1 |
| KIAA0895L | NM_001040715 | KIAA0895-like | ZNF174 | NM_003450 | zinc finger protein 174 | RPS6KC1 | NM_001287220 | ribosomal protein S6 kinase C1 |
| TNFAIP8L1 | NM_152362 | TNF alpha-induced protein 8 like 1 | ZNF597 | NM_152457 | zinc finger protein 597 | SNHG5 | NR_003038 | small nucleolar RNA host gene 5 |
| SUFU | NM_016169 | SUFU negative regulator of hedgehog signaling | STX5 | NM_003164 | syntaxin 5 | CASC1 | NM_001082972 | cancer susceptibility candidate 1 |
| PPCDC | NM_021823 | phosphopantothenoylcysteine decarboxylase | XYLT1 | NM_022166 | xylosyltransferase 1 | CSF2RA | NM_001161531_1 | Colony-stimulating factor 2 receptor alpha subunit |
| NMT2 | NM_004808 | N-myristoyltransferase 2 | TIPIN | NM_001289986 | TIMELESS-interacting protein | WDFY2 | NM_052950 | WD repeat and FYVE domain containing 2 |
| MAP4K3 | NM_003618 | mitogen-activated protein kinase kinase kinase kinase 3 | ACSM5 | NM_017888 | acyl-CoA synthetase medium-chain family member 5 | NFE2L3 | NM_004289 | nuclear factor, erythroid 2-like 3 |
| GINS1 | NM_021067 | GINS complex subunit 1 | COG7 | NM_153603 | component of oligomeric golgi complex 7 | NOD2 | NM_022162 | nucleotide-binding oligomerization domain containing 2 |
| RPL13AP6 | NR_026715 | ribosomal protein L13a pseudogene 6 | GGA2 | NM_015044 | golgi associated, gamma adaptin ear containing, ARF-binding protein 2 | MTRF1 | NM_004294 | mitochondrial translational release factor 1 |
| TKFC | NM_015533 | triokinase and FMN cyclase | CHI3L2 | NM_001025197 | chitinase 3 like 2 | CD209 | NM_001144893 | CD209 molecule |
| IDH2 | NM_002168 | isocitrate dehydrogenase (NADP(+)) 2, mitochondrial | ZCCHC4 | NM_024936 | zinc finger CCHC-type containing 4 | PTGIR | NM_000960 | prostaglandin I2 (prostacyclin) receptor (IP) |
| UQCRQ | NM_014402 | ubiquinol-cytochrome c reductase complex III subunit VII | MYRF | NM_001127392 | myelin regulatory factor | CMPK2 | NM_001256478 | cytidine/uridine monophosphate kinase 2 |
| BRF1 | NM_001519 | BRF1, RNA polymerase III transcription initiation factor 90 kDa subunit | SNAPC2 | NM_003083 | small nuclear RNA-activating complex polypeptide 2 | ZNF708 | NM_001297560 | zinc finger protein 708 |
| MRPL12 | NM_002949 | mitochondrial ribosomal protein L12 | ZNF688 | NM_145271 | zinc finger protein 688 | ICAM2 | NM_001099788 | intercellular adhesion molecule 2 |
| PGM2L1 | NM_173582 | phosphoglucomutase 2-like 1 | FBXL19-AS1 | NR_024348 | FBXL19 antisense RNA 1 (head to head) | PRR29 | NM_001191031 | proline-rich 29 |
| CRTAM | NM_019604 | cytotoxic and regulatory T-cell molecule | DTYMK | NM_001165031 | deoxythymidylate kinase | PRR5L | NM_001160167 | proline-rich 5-like |
| HNRNPUL2-BSCL2 | NR_037946 | HNRNPUL2-BSCL2 readthrough (NMD candidate) | UNG | NM_080911 | uracil DNA glycosylase | NCF1B | NR_003186 | neutrophil cytosolic factor 1B pseudogene |
| BCKDK | NM_001122957 | branched chain ketoacid dehydrogenase kinase | CNEP1R1 | NM_153261 | CTD nuclear envelope phosphatase 1 regulatory subunit 1 | NAAA | NM_001042402 | N-acylethanolamine acid amidase |
| ADAT3 | NM_138422 | adenosine deaminase, tRNA specific 3 | LPCAT2 | NM_017839 | lysophosphatidylcholine acyltransferase 2 | PTPN4 | NM_002830 | protein tyrosine phosphatase, non-receptor type 4 |
| CLUAP1 | NM_015041 | clusterin associated protein 1 | SLC36A4 | NM_152313 | solute carrier family 36 member 4 | RINT1 | NM_021930 | RAD50 interactor 1 |
| RNU4ATAC | NR_023343 | RNA, U4atac small nuclear (U12-dependent splicing) | ALDH1A1 | NM_000689 | aldehyde dehydrogenase 1 family member A1 | SLC35F3 | NM_173508 | solute carrier family 35 member F3 |
| CARHSP1 | NM_001278266 | calcium-regulated heat-stable protein 1 | ZDHHC1 | NM_013304 | zinc finger DHHC-type containing 1 | HIVEP2 | NM_006734 | human immunodeficiency virus type I enhancer-binding protein 2 |
| PARN | NM_001134477 | poly(A)-specific ribonuclease | CTRL | NM_001907 | chymotrypsin like | DNM3 | NM_001136127 | dynamin 3 |
| RRN3 | NM_018427 | RRN3 homolog, RNA polymerase I transcription factor | DPEP2 | NM_022355 | dipeptidase 2 | SLC40A1 | NM_014585 | solute carrier family 40 member 1 |
| SMG1 | NM_015092 | SMG1 phosphatidylinositol 3-kinase-related kinase | MRM1 | NM_024864 | mitochondrial rRNA methyltransferase 1 | TRIQK | NM_001191036 | triple QxxK/R motif containing |
| DNAJC4 | NM_005528 | DnaJ heat shock protein family (Hsp40) member C4 | PMFBP1 | NM_001160213 | polyamine modulated factor 1-binding protein 1 | TOP2A | NM_001067 | topoisomerase (DNA) II alpha 170kDa |
| CCP110 | NM_001199022 | centriolar coiled-coil protein 110kDa | ACAT1 | NM_000019 | acetyl-CoA acetyltransferase 1 | RNASE1 | NM_198232 | ribonuclease A family member 1, pancreatic |
| C16orf62 | NM_020314 | chromosome 16 open reading frame 62 | GPRC5B | NM_001304771 | G protein-coupled receptor class C group 5 member B | IL1RAP | NM_002182 | interleukin 1 receptor accessory protein |
| XPNPEP3 | NM_001204827 | X-prolyl aminopeptidase 3, mitochondrial | ZFP1 | NM_153688 | ZFP1 zinc finger protein | STC2 | NM_003714 | stanniocalcin 2 |
| SCX | NM_001080514_1 | scleraxis bHLH transcription factor | CLCN3 | NM_173872 | chloride voltage-gated channel 3 | FHL1 | NR_027621 | four and a half LIM domains 1 |
| ATXN2L | NM_148416 | ataxin 2-like | TMEM231 | NM_001077418 | transmembrane protein 231 | PPBP | NM_002704 | pro-platelet basic protein |
| RABEP2 | NM_024816 | rabaptin, RAB GTPase-binding effector protein 2 | HSDL1 | NM_001146051 | hydroxysteroid dehydrogenase like 1 | USP54 | NM_152586 | Ubiquitin-specific peptidase 54 |
| GTF2E1 | NM_005513 | general transcription factor IIE subunit 1 | PIM1 | NM_002648 | Pim-1 proto-oncogene, serine/threonine kinase | PPP2R2B | NM_001271948 | protein phosphatase 2 regulatory subunit B, beta |
| LAT | NM_001014989 | linker for activation of T-cells | TFPT | NM_013342 | TCF3 (E2A) fusion partner (in childhood leukemia) | S100B | NM_006272 | S100 calcium-binding protein B |
| INO80E | NM_173618 | INO80 complex subunit E | FAM114A2 | NM_018691 | family with sequence similarity 114 member A2 | ZBTB6 | NM_006626 | zinc finger and BTB domain containing 6 |
| PFDN6 | NM_001265596_4 | prefoldin subunit 6 | CHMP5 | NM_001195536 | charged multivesicular body protein 5 | SIT1 | NM_014450 | signaling threshold-regulating transmembrane adaptor 1 |
| ZNF747 | NM_001305019 | zinc finger protein 747 | SPATA33 | NM_153025 | spermatogenesis associated 33 | MAN1B1-AS1 | NR_027447 | MAN1B1 antisense RNA 1 (head to head) |
| QRSL1 | NM_018292 | glutaminyl-tRNA synthase (glutamine-hydrolyzing)-like 1 | FOLR2 | NM_001113534 | folate receptor beta | GPR143 | NM_000273 | G protein-coupled receptor 143 |
| RALGAPB | NM_001282917 | Ral GTPase activating protein non-catalytic beta subunit | LBX2-AS1 | NR_024606 | LBX2 antisense RNA 1 | GPR82 | NM_080817 | G protein-coupled receptor 82 |
| SLC48A1 | NM_017842 | solute carrier family 48 member 1 | SPATA1 | NM_001310156 | Spermatogenesis-associated 1 | ZXDB | NM_007157 | zinc finger, X-linked, duplicated B |
| WDR63 | NM_145172 | WD repeat domain 63 | IER3IP1 | NM_016097 | immediate early response 3-interacting protein 1 | MS4A6A | NM_152851 | membrane spanning 4-domains A6A |
| DHRS3 | NM_004753 | dehydrogenase/reductase (SDR family) member 3 | PNRC2 | NM_017761 | proline-rich nuclear receptor coactivator 2 | FOSB | NM_006732 | FosB proto-oncogene, AP-1 trancription factor subunit |
| PRMT9 | NM_138364 | protein arginine methyltransferase 9 | DVL2 | NM_004422 | dishevelled segment polarity protein 2 | PI3 | NM_002638 | peptidase inhibitor 3 |
| RAB18 | NM_001256411 | RAB18, member RAS oncogene family | OLFML3 | NM_020190 | olfactomedin-like 3 | GPBAR1 | NM_170699 | G protein-coupled bile acid receptor 1 |
| ISCU | NM_014301 | iron-sulfur cluster assembly enzyme | POR | NM_000941 | cytochrome p450 oxidoreductase | FPR1 | NM_001193306 | formyl peptide receptor 1 |
| PSKH1 | NM_006742 | protein serine kinase H1 | ADORA2B | NM_000676 | adenosine A2b receptor | CCL7 | NM_006273 | C-C motif chemokine ligand 7 |
| USP30 | NM_001301175 | Ubiquitin-specific peptidase 30 | HSPBP1 | NM_001297600 | HSPA (heat shock 70kDa)-binding protein, cytoplasmic cochaperone 1 | F13A1 | NM_000129 | coagulation factor XIII A chain |
| NFAT5 | NM_006599 | nuclear factor of activated T-cells 5, tonicity-responsive | RAI1 | NM_030665 | retinoic acid induced 1 | HBA1 | NM_000558 | hemoglobin subunit alpha 1 |
| SYCE1L | NM_001129979 | synaptonemal complex central element protein 1 like | TRAF4 | NM_004295 | TNF receptor-associated factor 4 | HBB | NM_000518 | hemoglobin subunit beta |
| NUDT7 | NM_001243657 | nudix hydrolase 7 | ANKS6 | NM_173551 | ankyrin repeat and sterile alpha motif domain containing 6 | APOE | NM_000041 | apolipoprotein E |
| CITED4 | NM_133467 | Cbp/p300-interacting transactivator with Glu/Asp rich carboxy-terminal domain 4 | GOLT1B | NM_016072 | golgi transport 1B | HS3ST2 | NM_006043 | heparan sulfate (glucosamine) 3-O-sulfotransferase 2 |
| SNAI3 | NM_178310 | snail family zinc finger 3 | HMOX1 | NM_002133 | heme oxygenase 1 | NMRK2 | NM_001289117 | nicotinamide riboside kinase 2 |
| APRT | NM_001030018 | adenine phosphoribosyltransferase | ERBB2 | NM_001289938 | erb-b2 receptor tyrosine kinase 2 | PDLIM4 | NM_001131027 | PDZ and LIM domain 4 |
| JRKL | NM_001261833 | JRK-like | CDC6 | NM_001254 | cell division cycle 6 | TM4SF19 | NM_001204897 | transmembrane 4 L six family member 19 |
| CCR5 | NM_001100168 | C-C motif chemokine receptor 5 (gene/pseudogene) | LIN7B | NM_001308419 | lin-7 homolog B, crumbs cell polarity complex component | PAQR5 | NM_017705 | progestin and adipoQ receptor family member 5 |
| MESDC1 | NM_022566 | mesoderm development candidate 1 | EEF1E1 | NM_001135650 | eukaryotic translation elongation factor 1 epsilon 1 | TM4SF19-TCTEX1D2 | NR_037950 | TM4SF19-TCTEX1D2 readthrough (NMD candidate) |
| PDCD6IP | NM_001256192 | programmed cell death 6-interacting protein | APC2 | NM_005883 | adenomatosis polyposis coli 2 | TM4SF19-AS1 | NR_121665 | TM4SF19 antisense RNA 1 |
| KIDINS220 | NM_020738 | kinase D-interacting substrate 220kDa | BRCA1 | NM_007299 | breast cancer 1 | PPP1R1A | NM_006741 | protein phosphatase 1 regulatory inhibitor subunit 1A |
| AGAP11 | NM_133447 | ArfGAP with GTPase domain, ankyrin repeat and PH domain 11 | RAP1GAP | NM_002885 | RAP1 GTPase activating protein | DCSTAMP | NM_001257317 | Dendrocyte-expressed seven transmembrane protein |
| CCDC174 | NM_016474 | coiled-coil domain containing 174 | IKBIP | NM_201613 | IKBKB-interacting protein | SPTBN4 | NM_020971 | spectrin beta, non-erythrocytic 4 |
| PFAS | NM_012393 | phosphoribosylformylglycinamidine synthase | SOCS7 | NM_014598 | suppressor of cytokine signaling 7 | APOC2 | NM_000483 | apolipoprotein C2 |
| CMTM8 | NM_178868 | CKLF like MARVEL transmembrane domain containing 8 | PTGR2 | NM_001146154 | prostaglandin reductase 2 | C11orf96 | NM_001145033 | chromosome 11 open reading frame 96 |
| FAM106A | NR_026809 | family with sequence similarity 106 member A | MYO6 | NM_001300899 | myosin VI | CTGF | NM_001901 | connective tissue growth factor |
| MFAP4 | NM_001198695 | microfibrillar associated protein 4 | PDK2 | NM_001199898 | pyruvate dehydrogenase kinase 2 | ANKRD29 | NM_173505 | ankyrin repeat domain 29 |
| KRT16P3 | NR_029393 | keratin 16 pseudogene 3 | ECSIT | NM_001243204 | ECSIT signaling integrator | MAN1C1 | NM_001289010 | mannosidase alpha class 1C member 1 |
| PYCR2 | NM_001271681 | pyrroline-5-carboxylate reductase family member 2 | CSF1R | NR_109969 | Colony-stimulating factor 1 receptor | PIGV | NM_017837 | phosphatidylinositol glycan anchor biosynthesis class V |
| NLK | NM_016231 | nemo-like kinase | PRKCA | NM_002737 | protein kinase C alpha | GMEB1 | NM_024482 | glucocorticoid modulatory element-binding protein 1 |
| NF1 | NM_000267 | neurofibromin 1 | CCDC96 | NM_153376 | coiled-coil domain containing 96 | HOXB6 | NM_018952 | homeobox B6 |
| TRMT1L | NM_030934 | tRNA methyltransferase 1 like | MRPL37 | NM_016491 | mitochondrial ribosomal protein L37 | IGF1 | NM_001111284 | insulin like growth factor 1 |
| DHRSX | NM_145177_1 | dehydrogenase/reductase (SDR family) X-linked | TPMT | NM_000367 | thiopurine S-methyltransferase | IGFN1 | NM_001164586 | immunoglobulin-like and fibronectin type III domain containing 1 |
| WDR26 | NM_001115113 | WD repeat domain 26 | CD164 | NM_001142404 | CD164 molecule | AK4 | NM_203464 | adenylate kinase 4 |
| ZNF281 | NM_001281294 | zinc finger protein 281 | TLCD1 | NM_001160407 | TLC domain containing 1 | IGFBP6 | NM_002178 | insulin-like growth factor-binding protein 6 |
| C7orf55-LUC7L2 | NM_001244584 | C7orf55-LUC7L2 readthrough | BET1 | NM_005868 | Bet1 golgi vesicular membrane trafficking protein | ZMYM1 | NM_001289088 | zinc finger MYM-type containing 1 |
| AAMDC | NM_024684 | adipogenesis-associated, Mth938 domain containing | SGSH | NM_000199 | N-sulfoglucosamine sulfohydrolase | INPP5B | NM_001297434 | inositol polyphosphate-5-phosphatase B |
| ACBD4 | NM_001135704 | acyl-CoA-binding domain containing 4 | FAM175B | NM_032182 | family with sequence similarity 175 member B | PPIEL | NR_003929 | peptidylprolyl isomerase E like pseudogene |
| SLC30A9 | NM_006345 | solute carrier family 30 member 9 | C9orf66 | NM_152569 | chromosome 9 open reading frame 66 | SPACA3 | NM_173847 | sperm acrosome associated 3 |
| GTF2IRD1P1 | NR_003934 | GTF2I repeat domain containing 1 pseudogene 1 | CEP76 | NR_073537 | centrosomal protein 76 | ARHGEF16 | NM_014448 | Rho guanine nucleotide exchange factor 16 |
| RPL26L1 | NM_016093 | ribosomal protein L26 like 1 | ABHD3 | NM_001308257 | abhydrolase domain containing 3 | ABCB5 | NM_001163941 | ATP-binding cassette subfamily B member 5 |
| FOXRED1 | NR_037647 | FAD-dependent oxidoreductase domain containing 1 | UGGT2 | NM_020121 | UDP-glucose glycoprotein glucosyltransferase 2 | ADAMTS14 | NM_080722 | ADAM metallopeptidase with thrombospondin type 1 motif 14 |
| CUL4A | NM_001278513 | cullin 4A | CHM | NM_000390 | CHM, Rab escort protein 1 | NOTCH3 | NM_000435 | notch 3 |
| TOM1L1 | NM_005486 | target of myb1-like 1 membrane trafficking protein | TMEM150A | NR_033179 | transmembrane protein 150A | LINC01010 | NR_038216 | long intergenic non-protein coding RNA 1010 |
| BIVM | NM_017693 | basic, immunoglobulin-like variable motif containing | GOLGA5 | NM_005113 | golgin A5 | KHDRBS3 | NM_006558 | KH domain containing, RNA-binding, signal transduction associated 3 |
| SC5D | NM_006918 | sterol-C5-desaturase | MACROD1 | NM_014067 | MACRO domain containing 1 | SLC12A8 | NM_001195483 | solute carrier family 12 member 8 |
| TRIM37 | NM_001005207 | tripartite motif containing 37 | SEC61G | NM_014302 | Sec61 translocon gamma subunit | LINC00504 | NR_126435 | long intergenic non-protein coding RNA 504 |
| POMZP3 | NM_152992 | POM121 and ZP3 fusion | INO80C | NM_194281 | INO80 complex subunit C | IGSF21 | NM_032880 | immunoglobin superfamily member 21 |
| PRKRIR | NR_130898 |  | ITGAX | NM_000887 | integrin subunit alpha X | GPC3 | NM_001164618 | glypican 3 |
| THBS3 | NM_007112 | thrombospondin 3 | QPRT | NM_014298 | quinolinate phosphoribosyltransferase | MLXIPL | NM_032951 | MLX-interacting protein-like |
| DLG5 | NM_004747 | discs large homolog 5 | PIH1D2 | NM_001082619 | PIH1 domain containing 2 | CADPS2 | NM_001009571 | Ca2+-dependent secretion activator 2 |
| COASY | NM_001042529 | coenzyme A synthase | FAM86EP | NR_130741 | family with sequence similarity 86 member E, pseudogene | LEPR | NM_001003679 | leptin receptor |
| CD300C | NM_006678 | CD300c molecule | ACAA2 | NM_006111 | acetyl-CoA acyltransferase 2 | DNASE2B | NM_021233 | deoxyribonuclease II beta |
| WDR3 | NM_006784 | WD repeat domain 3 | LMAN1 | NM_005570 | lectin, mannose-binding 1 | KCNMA1 | NM_001014797 | potassium calcium-activated channel subfamily M alpha 1 |
| MBNL2 | NM_001306070 | muscleblind-like splicing regulator 2 | PIGN | NM_012327 | phosphatidylinositol glycan anchor biosynthesis class N | CXCL5 | NM_002994 | C-X-C motif chemokine ligand 5 |
| BCL6 | NM_001706 | B-cell CLL/lymphoma 6 | OAZ3 | NM_001134939 | ornithine decarboxylase antizyme 3 | CYP27A1 | NM_000784 | cytochrome P450 family 27 subfamily A member 1 |
| SUSD6 | NM_014734 | sushi domain containing 6 | DOHH | NM_031304 | deoxyhypusine hydroxylase/monooxygenase | RPAP2 | NM_024813 | RNA polymerase II-associated protein 2 |
| BTBD1 | NM_001011885 | BTB domain containing 1 | REXO1 | NM_020695 | REX1, RNA exonuclease 1 homolog | C17orf97 | NM_001013672 | chromosome 17 open reading frame 97 |
| PRPSAP1 | NM_002766 | phosphoribosyl pyrophosphate synthetase-associated protein 1 | SF3A2 | NM_007165 | splicing factor 3a subunit 2 | ABCD3 | NM_002858 | ATP-binding cassette subfamily D member 3 |
| DPCD | NM_015448 | deleted in primary ciliary dyskinesia homolog (mouse) | ZNF555 | NM_001172775 | zinc finger protein 555 | TRMT13 | NM_019083 | tRNA methyltransferase 13 homolog |
| SCARNA16 | NR_003013 | small Cajal body-specific RNA 16 | LOC100130093 | NR_024485 |  | LOC101928227 | NR_120330 | uncharacterized LOC101928227 |
| DYRK1B | NM_006484 | dual-specificity tyrosine phosphorylation regulated kinase 1B | MPP3 | NR_003562 | membrane palmitoylated protein 3 | FERMT2 | NM_001134999 | fermitin family member 2 |
| STRA13 | NM_001271007 | stimulated by retinoic acid 13 | NUS1 | NM_138459 | NUS1 dehydrodolichyl diphosphate synthase subunit | DCLK2 | NR_036614 | doublecortin-like kinase 2 |
| USP32 | NM_032582 | ubiquitin-specific peptidase 32 | VMAC | NM_001017921 | vimentin-type intermediate filament associated coiled-coil protein | STXBP1 | NM_003165 | Syntaxin-binding protein 1 |
| FN3KRP | NM_024619 | fructosamine 3 kinase-related protein | UFM1 | NR_104584 | ubiquitin-fold modifier 1 | LOC103091866 | NR_111955 | uncharacterized LOC103091866 |
| ID2 | NM_002166 | inhibitor of DNA-binding 2, HLH protein | ZNF561 | NM_152289 | zinc finger protein 561 | OBSL1 | NM_015311 | obscurin-like 1 |
| NAP1L1 | NM_139207 | nucleosome assembly protein 1-like 1 | FBXL12 | NM_017703 | F-box and leucine-rich repeat protein 12 | ARHGEF5 | NM_005435 | Rho guanine nucleotide exchange factor 5 |
| UQCRHL | NM_001089591 | ubiquinol-cytochrome c reductase hinge protein like | COG6 | NR_026745 | component of oligomeric golgi complex 6 | FLG-AS1 | NR_103778 | FLG antisense RNA 1 |
| MIB1 | NM_020774 | mindbomb E3 ubiquitin protein ligase 1 | HS6ST1 | NM_004807 | heparan sulfate 6-O-sulfotransferase 1 | FLG2 | NM_001014342 | filaggrin family member 2 |
| IPO11-LRRC70 | NR_073584 | IPO11-LRRC70 readthrough | C19orf38 | NM_001136482 | chromosome 19 open reading frame 38 | KPRP | NM_001025231 | keratinocyte proline-rich protein |
| RNPS1 | NR_104485 | RNA-binding protein with serine rich domain 1 | ARHGEF1 | NM_004706 | Rho guanine nucleotide exchange factor 1 | LOR | NM_000427 | loricrin |
| ATF6 | NM_007348 | activating transcription factor 6 | DENND6A | NM_152678 | DENN domain containing 6A | CERCAM | NM_001286760 | cerebral endothelial cell adhesion molecule |
| TRAF3IP1 | NM_001139490 | TRAF3-interacting protein 1 | HLA-DRB1 | NM_001243965 | major histocompatibility complex, class II, DR beta 1 | GPC1 | NM_002081 | glypican 1 |
| TARS | NM_001258437 | threonyl-tRNA synthetase | TBC1D23 | NM_018309 | TBC1 domain family member 23 | CTSZ | NM_001336 | cathepsin Z |
| WDR5B | NM_019069 | WD repeat domain 5B | HOOK2 | NM_001100176 | hook microtubule-tethering protein 2 | PKLR | NM_181871 | pyruvate kinase, liver and RBC |
| CIRBP-AS1 | NR_027271 | CIRBP antisense RNA 1 | ADGRE3 | NM_001289158 | adhesion G protein-coupled receptor E3 | UNC5B | NM_001244889 | unc-5 netrin receptor B |
| XIAP | NM_001204401 | X-linked inhibitor of apoptosis, E3 ubiquitin protein ligase | CYP4F22 | NM_173483 | cytochrome P450 family 4 subfamily F member 22 | NCKAP5 | NM_207481 | NCK-associated protein 5 |
| TMX3 | NM_019022 | thioredoxin related transmembrane protein 3 | ZNF506 | NM_001145404 | zinc finger protein 506 | METRN | NM_024042 | meteorin, glial cell differentiation regulator |
| CYHR1 | NM_138496 | cysteine/histidine-rich 1 | ZNF714 | NR_117087 | zinc finger protein 714 | LOC171391 | NR_126342 | uncharacterized LOC171391 |
| FSTL3 | NM_005860 | follistatin like 3 | SBSN | NM_001166034 | suprabasin | SCARB1 | NM_001082959 | scavenger receptor class B member 1 |
| TMEM5 | NM_014254 | transmembrane protein 5 | U2AF1L4 | NM_001040425 | U2 small nuclear RNA auxiliary factor 1-like 4 | IGSF8 | NM_052868 | immunoglobulin superfamily member 8 |
| RFC5 | NM_001206801 | replication factor C subunit 5 | LIN37 | NM_019104 | lin-37 DREAM MuvB core complex component | COL6A2 | NM_001849 | collagen type VI alpha 2 |
| GMCL1 | NM_178439 | germ cell-less, spermatogenesis associated 1 | ZNF146 | NM_001099639 | zinc finger protein 146 | DUSP12 | NM_007240 | dual-specificity phosphatase 12 |
| NCLN | NM_020170 | nicalin | ZNF420 | NM_144689 | zinc finger protein 420 | UPK3BL | NM_001114403 | uroplakin 3B-like |
| UBA6 | NM_018227 | Ubiquitin-like modifier activating enzyme 6 | ZNF569 | NM_152484 | zinc finger protein 569 | RHBDL1 | NM_001278721 | rhomboid, veinlet-like 1 (Drosophila) |
| UBFD1 | NM_019116 | ubiquitin family domain containing 1 | CAPN12 | NM_144691 | calpain 12 | DDX60 | NM_017631 | DEXD/H-box helicase 60 |
| CLDN7 | NM_001307 | claudin 7 | ZNF574 | NM_022752 | zinc finger protein 574 | CRLF1 | NM_004750 | cytokine receptor-like factor 1 |
| TRAPPC5 | NM_001042462 | trafficking protein particle complex 5 | LOC101930071 | NR_126041 | uncharacterized LOC101930071 | POLR2J2 | NM_032959 | polymerase (RNA) II subunit J2 |
| RNASE6 | NM_005615 | ribonuclease A family member k6 | PHLDB3 | NM_198850 | pleckstrin homology-like domain family B member 3 | GALNT12 | NM_024642 | polypeptide N-acetylgalactosaminyltransferase 12 |
| APIP | NM_015957 | APAF1-interacting protein | TSEN15 | NM_001300766 | tRNA-splicing endonuclease subunit 15 | ARMC9 | NM_025139 | armadillo repeat containing 9 |
| SIKE1 | NR_049741 | suppressor of IKBKE 1 | ZNF230 | NM_006300 | zinc finger protein 230 | PEX6 | NM_000287 | peroxisomal biogenesis factor 6 |
| DNASE2 | NM_001375 | deoxyribonuclease II, lysosomal | GEMIN7 | NM_001007270 | gem nuclear organelle-associated protein 7 | PKP1 | NM_001005337 | plakophilin 1 |
| C19orf52 | NM_138358 | chromosome 19 open reading frame 52 | DMWD | NM_004943 | dystrophia myotonica, WD repeat containing | CHCHD6 | NM_032343 | coiled-coil-helix-coiled-coil-helix domain containing 6 |
| EPOR | NR_033663 | erythropoietin receptor | C5AR2 | NM_001271749 | complement component 5a receptor 2 | TMEM44 | NM_138399 | transmembrane protein 44 |
| FAM35A | NM_019054 | family with sequence similarity 35 member A | KPTN | NM_007059 | kaptin (actin-binding protein) | TMEM255A | NM_001104544 | transmembrane protein 255A |
| GSTP1 | NM_000852 | glutathione S-transferase pi 1 | SPHK2 | NM_001204158 | sphingosine kinase 2 | SLC29A2 | NM_001300868 | solute carrier family 29 member 2 |
| MZT2A | NM_001085365 | mitotic spindle organizing protein 2A | SLC6A16 | NM_014037 | solute carrier family 6 member 16 | KLRG1 | NM_005810 | killer cell lectin-like receptor G1 |
| FCHO1 | NM_001161358 | FCH domain only 1 | FCGRT | NM_001136019 | Fc fragment of IgG receptor and transporter | TAF1A | NM_001201536 | TATA-box binding protein-associated factor, RNA polymerase I subunit A |
| LRFN3 | NM_024509 | leucine rich repeat and fibronectin type III domain containing 3 | ARID1B | NM_020732 | AT-rich interaction domain 1B | DHRS11 | NM_024308 | dehydrogenase/reductase (SDR family) member 11 |
| CHD6 | NM_032221 | chromodomain helicase DNA-binding protein 6 | SLC35B3 | NM_001142541 | solute carrier family 35 member B3 | SPOCD1 | NM_001281988 | SPOC domain containing 1 |
| ACTN4 | NM_004924 | actinin alpha 4 | ZNF667-AS1 | NR_036521 | ZNF667 antisense RNA 1 (head to head) | SLC2A5 | NM_003039 | solute carrier family 2 member 5 |
| DIEXF | NM_014388 | digestive organ expansion factor homolog (zebrafish) | AURKC | NM_001015879 | aurora kinase C | CALML5 | NM_017422 | calmodulin-like 5 |
| CNFN | NM_032488 | cornifelin | TAF2 | NM_003184 | TATA-box binding protein-associated factor 2 | ANKRD16 | NM_019046 | ankyrin repeat domain 16 |
| RAB3GAP2 | NM_012414 | RAB3 GTPase activating non-catalytic protein subunit 2 | ZNF160 | NM_001102603 | zinc finger protein 160 | RASA4B | NM_001277335 | RAS p21 protein activator 4B |
| UBE2B | NM_003337 | Ubiquitin-conjugating enzyme E2 B | BLM | NM_000057 | Bloom syndrome RecQ like helicase | DLGAP1-AS1 | NR_024101 | DLGAP1 antisense RNA 1 |
| MYPOP | NM_001012643 | Myb-related transcription factor, partner of profilin | E2F6 | NM_001278278 | E2F transcription factor 6 | TNFSF14 | NM_003807 | tumor necrosis factor superfamily member 14 |
| C19orf68 | NM_199341 | chromosome 19 open reading frame 68 | TTC32 | NM_001008237 | tetratricopeptide repeat domain 32 | SEMA7A | NM_003612 | semaphorin 7A (John Milton Hagen blood group) |
| KLK7 | NM_001243126 | Kallikrein-related peptidase 7 | XPR1 | NM_001135669 | xenotropic and polytropic retrovirus receptor 1 | FLJ21408 | NR_037184 | uncharacterized LOC400512 |
| ATL2 | NM_001308076 | atlastin GTPase 2 | ARCN1 | NM_001655 | archain 1 | GRIN2D | NM_000836 | glutamate ionotropic receptor NMDA type subunit 2D |
| LENG8 | NM_052925 | leukocyte receptor cluster (LRC) member 8 | AK9 | NM_001145128 | adenylate kinase 9 | LTBP3 | NM_001130144 | latent transforming growth factor-beta-binding protein 3 |
| LENG9 | NM_001301782 | leukocyte receptor cluster (LRC) member 9 | MYO1D | NM_015194 | myosin ID | ST18 | NM_014682 | suppression of tumorigenicity 18, zinc finger |
| WAS | NM_000377 | Wiskott-Aldrich syndrome | DYNC1LI1 | NM_016141 | dynein cytoplasmic 1 light intermediate chain 1 | PDXP | NM_020315 | pyridoxal phosphatase |
| TNNT1 | NM_001291774 | troponin T1, slow skeletal type | FAM193B | NM_001190946 | family with sequence similarity 193 member B | BMS1P4 | NR_026592 | BMS1, ribosome biogenesis factor pseudogene 4 |
| ZNF264 | NM_003417 | zinc finger protein 264 | THRIL | NR_110375 | TNF and HNRNPL related immunoregulatory long non-coding RNA | RHOF | NM_019034 | ras homolog family member F (in filopodia) |
| DYM | NM_017653 | dymeclin | FANCL | NM_001114636 | Fanconi anemia complementation group L | LRRC16A | NM_017640 | leucine-rich repeat containing 16A |
| ZNF417 | NM_001297734 | zinc finger protein 417 | TMED7 | NM_181836 | transmembrane p24 trafficking protein 7 | ATAD1 | NM_032810 | ATPase family, AAA domain containing 1 |
| TIMELESS | NM_003920 | timeless circadian clock | C2orf74 | NM_001143960 | chromosome 2 open reading frame 74 | NAPRT | NM_001286829 | nicotinate phosphoribosyltransferase |
| AFG3L1P | NR_003227 | AFG3 like matrix AAA peptidase subunit 1, pseudogene | ZNF83 | NM_001277949 | zinc finger protein 83 | CRNDE | NM_001308963 | colorectal neoplasia differentially expressed (non-protein coding) |
| PUM2 | NM_001282791 | pumilio RNA-binding family member 2 | ALOX5AP | NM_001204406 | arachidonate 5-lipoxygenase-activating protein | OTUB2 | NM_023112 | OTU deubiquitinase, ubiquitin aldehyde-binding 2 |
| LDAH | NM_021925 | lipid droplet-associated hydrolase | CHEK1 | NM_001244846 | checkpoint kinase 1 | PIPSL | NR_002319 | PIP5K1A and PSMD4-like, pseudogene |
| FUBP3 | NM_003934 | far upstream element-binding protein 3 | ACSL1 | NM_001286708 | acyl-CoA synthetase long-chain family member 1 | GPR84 | NM_020370 | G protein-coupled receptor 84 |
| SLC4A1AP | NM_018158 | solute carrier family 4 member 1 adaptor protein | LOC100507564 | NR_038953 | uncharacterized LOC100507564 | EXOSC1 | NM_016046 | exosome component 1 |
| KAT7 | NM_001199155 | K(lysine) acetyltransferase 7 | SFXN5 | NM_144579 | sideroflexin 5 | SNAR-A8 | NR_024228 | small ILF3/NF90-associated RNA A8 |
| GCNT1 | NM_001097634 | glucosaminyl (N-acetyl) transferase 1, core 2 | CTBP1-AS2 | NR_033339 | CTBP1 antisense RNA 2 (head to head) | FERMT1 | NM_017671 | fermitin family member 1 |
| CRIM1 | NM_016441 | cysteine rich transmembrane BMP regulator 1 (chordin-like) | MOB1A | NM_018221 | MOB kinase activator 1A | FA2H | NM_024306 | fatty acid 2-hydroxylase |
| CCZ1 | NM_015622 | CCZ1 homolog, vacuolar protein trafficking and biogenesis associated | PTGDR2 | NM_004778 | prostaglandin D2 receptor 2 | CALHM2 | NM_015916 | calcium homeostasis modulator 2 |
| C17orf80 | NM_001288771 | chromosome 17 open reading frame 80 | KIAA0907 | NM_014949 | KIAA0907 | TSPAN15 | NM_012339 | tetraspanin 15 |
| EML4 | NM_001145076 | echinoderm microtubule associated protein like 4 | SEMA4F | NM_004263 | ssemaphorin 4F | TMEM178A | NM_001167959 | transmembrane protein 178A |
| SOS1 | NM_005633 | SOS Ras/Rac guanine nucleotide exchange factor 1 | INTS2 | NM_020748 | integrator complex subunit 2 | CD80 | NM_005191 | CD80 molecule |
| GPR75-ASB3 | NM_001164165 | GPR75-ASB3 readthrough | KDM1B | NM_153042 | lysine demethylase 1B | WDR62 | NM_173636 | WD repeat domain 62 |
| AHSA2 | NM_152392 | AHA1, activator of heat shock 90kDa protein ATPase homolog 2 (yeast) | ADRA2B | NM_000682 | adrenoceptor alpha 2B | NRN1 | NM_001278710 | neuritin 1 |
| USP34 | NM_014709 | Ubiquitin-specific peptidase 34 | PHF10 | NM_133325 | PHD finger protein 10 | RASSF7 | NM_001143993 | Ras association domain family member 7 |
| C2orf68 | NM_001013649 | chromosome 2 open reading frame 68 | MRPS30 | NM_016640 | mitochondrial ribosomal protein S30 | CRACR2B | NM_173584 | calcium release-activated channel regulator 2B |
| NKRF | NM_001173488 | NFKB-repressing factor | ADAMTSL4-AS1 | NR_104133 | ADAMTSL4 antisense RNA 1 | GALNT18 | NM_198516 | polypeptide N-acetylgalactosaminyltransferase 18 |
| REV3L | NM_002912 | REV3 like, DNA directed polymerase zeta catalytic subunit | GCC2 | NR_028063 | GRIP and coiled-coil domain containing 2 | ZNF195 | NM_001130520 | zinc finger protein 195 |
| RETSAT | NM_017750 | retinol saturase (all-trans-retinol 13,14-reductase) | RANBP2 | NM_006267 | RNA-binding protein 2 | SNORD50A | NR_002743 | small nucleolar RNA, C/D box 50A |
| GNL2 | NM_013285 | G protein nucleolar 2 | MEX3C | NM_016626 | mex-3 RNA-binding family member C | HOXB5 | NM_002147 | homeobox B5 |
| ALG13 | NM_001099922 | ALG13, UDP-N-acetylglucosaminyltransferase subunit | ATP6V1E2 | NM_080653 | ATPase H+ transporting V1 subunit E2 | ACOT7 | NM_007274 | acyl-CoA thioesterase 7 |
| AQR | NM_014691 | aquarius intron-binding spliceosomal factor | KIAA1429 | NM_015496 | KIAA1429 | CYB5R2 | NM_001302826 | cytochrome b5 reductase 2 |
| INPP4A | NM_001566 | inositol polyphosphate-4-phosphatase type I A | CBWD3 | NM_201453 | COBW domain containing 3 | DTNB | NM_001256308 | dystrobrevin beta |
| TCEA1 | NM_201437 | transcription elongation factor A1 | NLRX1 | NM_001282358 | NLR family member X1 | NCAPH | NM_001281710 | non-SMC condensin I complex subunit H |
| NUDT21 | NM_007006 | nudix hydrolase 21 | TAF1D | NM_024116 | TATA-box binding protein-associated factor, RNA polymerase I subunit D | IFT140 | NM_014714 | intraflagellar transport 140 |
| GTPBP10 | NM_001042717 | GTP-binding protein 10 (putative) | SUCNR1 | NM_033050 | succinate receptor 1 | PIGR | NM_002644 | polymeric immunoglobulin receptor |
| CLDN12 | NM_001185072 | claudin 12 | GTDC1 | NM_001284233 | Glycosyltransferase-like domain containing 1 | EFNA3 | NM_004952 | ephrin A3 |
| CFL1P1 | NR_028492 | cofilin 1 pseudogene 1 | ARL6IP6 | NR_024526 | ADP ribosylation factor-like GTPase 6-interacting protein 6 | APOD | NM_001647 | apolipoprotein D |
| GPCPD1 | NM_019593 | glycerophosphocholine phosphodiesterase 1 | BCAS2 | NM_005872 | breast carcinoma amplified sequence 2 | LOC101927204 | NR_110184 | uncharacterized LOC101927204 |
| ZNF451 | NM_001257273 | zinc finger protein 451 | KCTD3 | NM_016121 | potassium channel tetramerization domain containing 3 | GGT1 | NM_013430 | gamma-glutamyltransferase 1 |
| SFT2D3 | NM_032740 | SFT2 domain containing 3 | TRIM33 | NM_033020 | tripartite motif containing 33 | RAMP1 | NM_005855 | receptor activity-modifying protein 1 |
| ZNF286A | NM_001288649 | zinc finger protein 286A | TANK | NM_133484 | TRAF family member associated NFKB activator | METTL12 | NM_001043229 | Methyltransferase-like 12 |
| IQCG | NM_001134435 | IQ motif containing G | AMN1 | NM_001278411 | antagonist of mitotic exit network 1 homolog | PITX1 | NM_002653 | paired-like homeodomain 1 |
| ZSCAN2 | NM_181877 | zinc finger and SCAN domain containing 2 | PCSK5 | NM_006200 | proprotein convertase subtilisin/kexin type 5 | PAX8 | NM_003466 | paired box 8 |
| CEP57 | NM_001243776 | centrosomal protein 57 | C10orf88 | NM_024942 | chromosome 10 open reading frame 88 | FAM43A | NM_153690 | family with sequence similarity 43 member A |
| GPD2 | NM_001083112 | glycerol-3-phosphate dehydrogenase 2 | CACNA1A | NM_000068 | calcium voltage-gated channel subunit alpha1 A | EFEMP2 | NR_037718 | EGF containing fibulin-like extracellular matrix protein 2 |
| MARCH7 | NM_001282806 | Membrane-associated ring-CH-type finger 7 | USP1 | NM_001017416 | Ubiquitin-specific peptidase 1 | STK32C | NM_173575 | serine/threonine kinase 32C |
| DFNB59 | NM_001042702 | deafness, autosomal recessive 59 | SCRN3 | NM_024583 | secernin 3 | RASA4CP | NR_024116 | RAS p21 protein activator 4C, pseudogene |
| PMS1 | NR_110332 | PMS1 homolog 1, mismatch repair system component | LOC101927027 | NR_110206 | uncharacterized LOC101927027 | RASA4 | NM_001079877 | RAS p21 protein activator 4 |
| SLC39A10 | NM_001127257 | solute carrier family 39 member 10 | DDX50 | NM_024045 | DEAD-box helicase 50 | DDAH1 | NM_012137 | dimethylarginine dimethylaminohydrolase 1 |
| SRSF1 | NR_034041 | serine/arginine-rich splicing factor 1 | CASC4 | NM_138423 | cancer susceptibility candidate 4 | GRN | NM_002087 | granulin |
| MYO9A | NM_006901 | myosin IXA | HIBCH | NM_198047 | 3-hydroxyisobutyryl-CoA hydrolase | CH17-340M24.3 | NR_103768 | uncharacterized protein BC009467 |
| TRPM7 | NM_017672 | transient receptor potential cation channel subfamily M member 7 | GLMN | NM_053274 | glomulin, FKBP-associated protein | TMEM262 | NM_001242631 | transmembrane protein 262 |
| TNIK | NM_001161566 | TRAF2 and NCK-interacting kinase | KCTD18 | NM_152387 | potassium channel tetramerization domain containing 18 | CCDC106 | NM_013301 | coiled-coil domain containing 106 |
| PDE6D | NM_002601 | phosphodiesterase 6D | COQ10B | NM_025147 | coenzyme Q10B | MURC | NM_001018116 | Muscle-related coiled-coil protein |
| ZBED5 | NM_001143667 | zinc finger BED-type containing 5 | HSPE1-MOB4 | NM_001202485 | HSPE1-MOB4 readthrough | LINC01128 | NR_047519 | long intergenic non-protein coding RNA 1128 |
| CLDND1 | NM_001040199 | claudin domain containing 1 | FBXO44 | NM_183412 | F-box protein 44 | LARGE | NM_004737 | like-glycosyltransferase |
| CCDC88B | NM_032251 | coiled-coil domain containing 88B | PLCL1 | NM_006226 | phospholipase C like 1 | SLC25A1 | NM_001287387 | solute carrier family 25 member 1 |
| MYEOV2 | NM_138336 |  | TYW5 | NR_109907 | tRNA-yW-synthesizing protein 5 | HDAC10 | NM_032019 | histone deacetylase 10 |
| ZNF140 | NM_003440 | zinc finger protein 140 | SUCLG2 | NM_001177599 | succinate-CoA ligase GDP-forming beta subunit | VWF | NM_000552 | von Willebrand factor |
| DNALI1 | NM_003462 | dynein axonemal light intermediate chain 1 | APOBEC3C | NM_014508 | apolipoprotein B mRNA-editing enzyme catalytic subunit 3C | TUBGCP4 | NM_014444 | tubulin gamma complex associated protein 4 |
| RAB28 | NM_001159601 | RAB28, member RAS oncogene family | ZNF850 | NM_001193552 | zinc finger protein 850 | PPARG | NM_005037 | peroxisome proliferator activated receptor gamma |
| BTAF1 | NM_003972 | B-TFIID TATA-box binding protein-associated factor 1 | CBWD2 | NM_172003 | COBW domain containing 2 | SPSB2 | NM_001146316 | splA/ryanodine receptor domain and SOCS box containing 2 |
| SLC23A2 | NM_005116 | solute carrier family 23 member 2 | WDR12 | NM_018256 | WD repeat domain 12 | ZNF366 | NM_152625 | zinc finger protein 366 |
| SNORD17 | NR_003045 | small nucleolar RNA, C/D box 17 | MAK | NM_001242957 | male germ cell-associated kinase | A2ML1 | NM_144670 | alpha-2-macroglobulin like 1 |
| SEC31B | NM_015490 | SEC31 homolog B, COPII coat complex component | NBEAL1 | NM_001114132 | neurobeachin-like 1 | CLEC1A | NM_001297748 | C-type lectin domain family 1 member A |
| CIDEB | NM_014430 | cell death-inducing DFFA-like effector b | USPL1 | NM_005800 | ubiquitin-specific peptidase-like 1 | OLR1 | NM_001172632 | oxidized low-density lipoprotein receptor 1 |
| BACH1 | NM_001186 | BTB domain and CNC homolog 1 | GCSHP3 | NR_033248 | glycine cleavage system protein H (aminomethyl carrier) pseudogene 3 | TMEM52B | NM_001079815 | transmembrane protein 52B |
| AHCY | NM_000687 | adenosylhomocysteinase | NCAPG | NM_022346 | non-SMC condensin I complex subunit G | TMTC1 | NM_001193451 | transmembrane and tetratricopeptide repeat containing 1 |
| PCIF1 | NM_022104 | PDX1 C-terminal inhibiting factor 1 | ARIH1 | NM_005744 | ariadne RBR E3 ubiquitin protein ligase 1 | ALG10 | NM_032834 | ALG10, alpha-1,2-glucosyltransferase |
| NCOA5 | NM_020967 | nuclear receptor coactivator 5 | PCMTD2 | NM_001104925 | protein-L-isoaspartate (D-aspartate) O-methyltransferase domain containing 2 | ALG10B | NM_001013620 | ALG10B, alpha-1,2-glucosyltransferase |
| GTF2A1 | NM_001278940 | general transcription factor IIA subunit 1 | C6orf203 | NM_016487 | chromosome 6 open reading frame 203 | COX5B | NM_001862 | cytochrome c oxidase subunit 5B |
| ZNF845 | NM_138374 | zinc finger protein 845 | TESC | NM_001168325 | tescalcin | CACNB3 | NM_001206917 | calcium voltage-gated channel auxiliary subunit beta 3 |
| POLR2J3 | NM_001097615 | polymerase (RNA) II subunit J3 | KIAA1407 | NM_020817 |  | SPATS2 | NR_120663 | spermatogenesis-associated serine rich 2 |
| TNFRSF6B | NM_003823 | tumor necrosis factor receptor superfamily member 6b | IFRD1 | NM_001197080 | interferon-related developmental regulator 1 | TMEM241 | NM_032933 | transmembrane protein 241 |
| USP25 | NM_001283041 | Ubiquitin-specific peptidase 25 | RUSC2 | NR_052015 | RUN and SH3 domain containing 2 | LETMD1 | NM_001300765 | LETM1 domain containing 1 |
| MRPL39 | NM_017446 | mitochondrial ribosomal protein L39 | ATL1 | NM_001127713 | atlastin GTPase 1 | KRT5 | NM_000424 | keratin 5 |
| MME | NM_000902 | membrane metallo-endopeptidase | SLC35E2 | NM_001199787 | solute carrier family 35 member E2 | QTRT1 | NM_031209 | queuine tRNA-ribosyltransferase catalytic subunit 1 |
| RBM38 | NM_183425 | RNA-binding motif protein 38 | TMEM242 | NM_018452 | transmembrane protein 242 | GGTLC2 | NM_001282879 | gamma-glutamyltransferase light chain 2 |
| SYT11 | NM_152280 | synaptotagmin 11 | TDRD7 | NM_014290 | Tudor domain containing 7 | PHLDA1 | NM_007350 | pleckstrin homology like domain family A member 1 |
| PCNXL3 | NM_032223 |  | CSE1L | NM_001316 | chromosome segregation 1-like | BBS10 | NM_024685 | Bardet-Biedl syndrome 10 |
| RFX3 | NM_001282116 | regulatory factor X3 | SLC12A2 | NR_046207 | solute carrier family 12 member 2 | TAF13 | NM_005645 | TATA-box binding protein-associated factor 13 |
| BCL2 | NM_000633 | B-cell CLL/lymphoma 2 | LOC727896 | NR_026659 | cysteine and histidine-rich domain containing 1 pseudogene | MRC2 | NM_006039 | mannose receptor C type 2 |
| RPL36AL | NM_001001 | ribosomal protein L36a-like | ZNF75D | NR_110381 | zinc finger protein 75D | ISOC2 | NM_024710 | isochorismatase domain containing 2 |
| MDM1 | NM_001205028 | Mdm1 nuclear protein | ZNF251 | NM_138367 | zinc finger protein 251 | YAE1D1 | NM_001282446 | Yae1 domain containing 1 |
| C9orf142 | NM_183241 | chromosome 9 open reading frame 142 | SMOX | NM_175840 | spermine oxidase | ACACB | NM_001093 | acetyl-CoA carboxylase beta |
| TXNRD2 | NM_006440 | thioredoxin reductase 2 | NOL8 | NM_017948 | nucleolar protein 8 | ANKRD6 | NM_001242809 | ankyrin repeat domain 6 |
| DECR1 | NM_001359 | 2,4-dienoyl-CoA reductase 1, mitochondrial | MSANTD2 | NR_131749 | Myb/SANT DNA-binding domain containing 2 | FBXO10 | NM_012166 | F-box protein 10 |
| CABIN1 | NM_001201429 | calcineurin-binding protein 1 | TGFBR2 | NM_001024847 | transforming growth factor beta receptor 2 | LINC00346 | NR_027701 | long intergenic non-protein coding RNA 346 |
| ASPHD2 | NM_020437 | aspartate beta-hydroxylase domain containing 2 | RAB8B | NM_016530 | RAB8B, member RAS oncogene family | FEM1C | NM_020177 | fem-1 homolog C |
| DUSP18 | NM_001304795 | dual-specificity phosphatase 18 | CTNNAL1 | NM_003798 | catenin alpha like 1 | SERPINI1 | NM_001122752 | serpin family I member 1 |
| LOC101928461 | NR_125858 | uncharacterized LOC101928461 | TTBK2 | NM_173500 | tau tubulin kinase 2 | POLR1B | NM_001282779 | polymerase (RNA) I subunit B |
| HMGXB4 | NR_027780 | HMG-box containing 4 | GNA13 | NM_006572 | G protein subunit alpha 13 | LRRC29 | NM_012163 | leucine-rich repeat containing 29 |
| LGALS3 | NM_001177388 | lectin, galactoside-binding soluble 3 | ZNF341 | NM_032819 | zinc finger protein 341 | AKT1S1 | NM_001098633 | AKT1 substrate 1 |
| LGALS1 | NM_002305 | lectin, galactoside-binding soluble 1 | ECD | NR_024203 | ecdysoneless cell cycle regulator | NQO2 | NM_000904 | NAD(P)H quinone dehydrogenase 2 |
| ST3GAL5 | NM_003896 | ST3 beta-galactoside alpha-2,3-sialyltransferase 5 | AAR2 | NM_001271874 | AAR2 splicing factor homolog | PEX14 | NM_004565 | peroxisomal biogenesis factor 14 |
| TEF | NM_001145398 | TEF, PAR bZIP transcription factor | MYL9 | NM_006097 | myosin light chain 9 | PIGU | NM_080476 | phosphatidylinositol glycan anchor biosynthesis class U |
| MEI1 | NM_152513 | meiotic double-stranded break formation protein 1 | ZNF746 | NM_152557 | zinc finger protein 746 | ZNF436 | NM_030634 | zinc finger protein 436 |
| C5 | NM_001735 | complement component 5 | HYPK | NM_001199885 | Huntingtin-interacting protein K | SCARNA4 | NR_003005 | small Cajal body-specific RNA 4 |
| SENP6 | NM_015571 | SUMO1/sentrin specific peptidase 6 | CCPG1 | NM_001204450 | cell cycle progression 1 | ANXA2 | NM_004039 | annexin A2 |
| PTPN18 | NM_001142370 | protein tyrosine phosphatase, non-receptor type 18 | PIK3C2B | NM_002646 | phosphatidylinositol-4-phosphate 3-kinase catalytic subunit type 2 beta | TMEM255B | NM_182614 | transmembrane protein 255B |
| PLD2 | NM_002663 | phospholipase D2 | C7orf43 | NM_018275 | chromosome 7 open reading frame 43 | ABCB8 | NM_001282292 | ATP-binding cassette subfamily B member 8 |
| POLH | NM_006502 | polymerase (DNA) eta | ABCD4 | NR_003256 | ATP-binding cassette subfamily D member 4 | P3H3 | NM_014262 | prolyl 3-hydroxylase 3 |
| RPUSD3 | NM_001142547 | RNA pseudouridylate synthase domain containing 3 | ADSS | NM_001126 | adenylosuccinate synthase | NRL | NM_006177 | neural retina leucine zipper |
| BTD | NM_001281723 | biotinidase | PARD6B | NM_032521 | par-6 family cell polarity regulator beta | RIPK3 | NM_006871 | receptor-interacting serine/threonine kinase 3 |
| ANKRD28 | NM_001195099 | ankyrin repeat domain 28 | SLC45A3 | NM_033102 | solute carrier family 45 member 3 | BTBD2 | NM_017797 | BTB domain containing 2 |
| SLIT1 | NM_003061 | slit guidance ligand 1 | KRBA2 | NM_001304947 | KRAB-A domain containing 2 | AP4S1 | NM_001254729 | adaptor-related protein complex 4 sigma 1 subunit |
| NKIRAS1 | NM_020345 | NFKB inhibitor-interacting Ras-like 1 | SNRPA1 | NM_003090 | small nuclear ribonucleoprotein polypeptide A' | BBS12 | NM_152618 | Bardet-Biedl syndrome 12 |
| FBXO41 | NM_001080410 | F-box protein 41 | EXOSC8 | NM_181503 | exosome component 8 | COBL | NM_001287436 | cordon-bleu WH2 repeat protein |
| GPD1L | NM_015141 | glycerol-3-phosphate dehydrogenase 1-like | C5orf51 | NM_175921 | chromosome 5 open reading frame 51 | AP5M1 | NM_018229 | adaptor-related protein complex 5 mu 1 subunit |
| CCDC34 | NM_030771 | coiled-coil domain containing 34 | ADNP2 | NM_014913 | ADNP homeobox 2 | LINC00622 | NR_036540 | long intergenic non-protein coding RNA 622 |
| DLEC1 | NM_007335 | deleted in lung and esophageal cancer 1 | TMEM238 | NM_001190764 | transmembrane protein 238 | MFGE8 | NM_005928 | milk fat globule-EGF factor 8 protein |
| PCNX | NM_001308160 |  | DDIAS | NM_145018 | DNA damage induced apoptosis suppressor | SEPT4 | NM_004574 | septin 4 |
| TUBB4B | NM_006088 | tubulin beta 4B class IVb | GORAB | NM_152281 | golgin, RAB6-interacting | ACAD10 | NM_001136538 | acyl-CoA dehydrogenase family member 10 |
| TTC14 | NM_001042601 | tetratricopeptide repeat domain 14 | ATP1B2 | NM_001303263 | ATPase Na+/K+ transporting subunit beta 2 | SAMD1 | NM_138352 | sterile alpha motif domain containing 1 |
| LINC00672 | NR_038847 | long intergenic non-protein coding RNA 672 | CRYZL1 | NM_145858 | crystallin zeta-like 1 | ENAH | NM_018212 | enabled homolog (Drosophila) |
| FAM133DP | NR_034169 | family with sequence similarity 133 member D, pseudogene | CCDC101 | NM_138414 |  | TDRD9 | NM_153046 | Tudor domain containing 9 |
| CD84 | NM_003874 | CD84 molecule | NDNL2 | NM_138704 |  | ZNF322 | NM_001242799 | zinc finger protein 322 |
| AEBP2 | NM_001114176 | AE-binding protein 2 | ZNF75A | NM_001302109 | zinc finger protein 75a | NDN | NM_002487 | necdin, MAGE family member |
| PM20D2 | NM_001010853 | peptidase M20 domain containing 2 | LZTFL1 | NM_001276379 | leucine zipper transcription factor-like 1 | CXCL3 | NM_002090 | C-X-C motif chemokine ligand 3 |
| PBRM1 | NM_018313 | polybromo 1 | GOLGA8A | NR_027409 | golgin A8 family member A | ACTC1 | NM_005159 | actin, alpha, cardiac muscle 1 |
| ARHGAP11A | NM_001286479 | Rho GTPase activating protein 11A | POFUT2 | NR_004858 | protein O-fucosyltransferase 2 | CHKB-AS1 | NR_021492 | CHKB antisense RNA 1 (head to head) |
| HNRNPA3P1 | NR_002726 | heterogeneous nuclear ribonucleoprotein A3 pseudogene 1 | RFC1 | NM_001204747 | replication factor C subunit 1 | CDAN1 | NM_138477 | codanin 1 |
| SRPK1 | NR_034069 | SRSF protein kinase 1 | ANP32E | NM_001280559 | acidic nuclear phosphoprotein 32 family member E | SPATA5L1 | NR_027635 | spermatogenesis-associated 5 like 1 |
| VRK1 | NM_003384 | vaccinia-related kinase 1 | SNORA12 | NR_002954 | small nucleolar RNA, H/ACA box 12 | DECR2 | NM_020664 | 2,4-dienoyl-CoA reductase 2, peroxisomal |
| METTL15 | NM_001297775 | Methyltransferase-like 15 | TMEM191A | NR_026815 | transmembrane protein 191A (pseudogene) | FAM227B | NM_152647 | family with sequence similarity 227 member B |
| CGGBP1 | NM_003663 | CGG triplet repeat binding protein 1 | PCNP | NM_020357 | PEST proteolytic signal containing nuclear protein | DCAF13P3 | NR_027642 | DDB1 and CUL4 associated factor 13 pseudogene 3 |
| CRYBG3 | NM_153605 | crystallin beta-gamma domain containing 3 | NINJ2 | NM_016533 | ninjurin 2 | GTF2A2 | NM_004492 | general transcription factor IIA subunit 2 |
| TRMT10C | NM_017819 | tRNA methyltransferase 10C, mitochondrial RNase P subunit | CRYBB2P1 | NR_033733 | crystallin beta B2 pseudogene 1 | ZFHX2 | NM_033400 | zinc finger homeobox 2 |
| RAD52 | NM_001297421 | RAD52 homolog, DNA repair protein | ZNF211 | NM_001265600 | zinc finger protein 211 | ANKDD1A | NM_182703 | ankyrin repeat and death domain containing 1A |
| SUZ12P1 | NR_024187 | SUZ12 polycomb repressive complex 2 subunit pseudogene 1 | YOD1 | NM_018566 | YOD1 deubiquitinase | CPTP | NM_001029885 | ceramide-1-phosphate transfer protein |
| RASSF3 | NR_040718 | Ras association domain family member 3 | OSBP2 | NM_001282738 | oxysterol-binding protein 2 | ZBTB47 | NM_145166 | zinc finger and BTB domain containing 47 |
| HEG1 | NM_020733 | heart development protein with EGF-like domains 1 | HSPA13 | NM_006948 | heat shock protein family A (Hsp70) member 13 | PPAT | NM_002703 | phosphoribosyl pyrophosphate amidotransferase |
| SNX4 | NM_003794 | sorting nexin 4 | PCGF3 | NM_006315 | polycomb group ring finger 3 | PSTPIP1 | NM_003978 | proline-serine-threonine phosphatase-interacting protein 1 |
| ABTB1 | NM_032548 | ankyrin repeat and BTB domain containing 1 | APOL4 | NM_145660 | apolipoprotein L4 | ACSF2 | NM_001288968 | acyl-CoA synthetase family member 2 |
| LY6E | NM_002346 | lymphocyte antigen 6 complex, locus E | LINC00869 | NR_111953 | long intergenic non-protein coding RNA 869 | IFT22 | NM_001287526 | intraflagellar transport 22 |
| ATAD2 | NM_014109 | ATPase family, AAA domain containing 2 | LINC00623 | NR_024511 | long intergenic non-protein coding RNA 623 | CHTF18 | NM_022092 | chromosome transmission fidelity factor 18 |
| CHST2 | NM_004267 | carbohydrate sulfotransferase 2 | WDR75 | NM_032168 | WD repeat domain 75 | SNHG9 | NR_003142 | small nucleolar RNA host gene 9 |
| MORC3 | NM_015358 | MORC family CW-type zinc finger 3 | GLI1 | NM_001160045 | GLI family zinc finger 1 | SOCS1 | NM_003745 | suppressor of cytokine signaling 1 |
| KIF5B | NM_004521 | kinesin family member 5B | IKZF5 | NR_073475 | IKAROS family zinc finger 5 | NPIPA7 | NM_001282507 | nuclear pore complex-interacting protein family member A7 |
| SMC4 | NM_001288753 | structural maintenance of chromosomes 4 | GALNT10 | NM_198321 | polypeptide N-acetylgalactosaminyltransferase 10 | ATP2A1 | NM_004320 | ATPase sarcoplasmic/endoplasmic reticulum Ca2+ transporting 1 |
| RIN1 | NM_004292 | Ras and Rab interactor 1 | CEP97 | NM_001303401 | centrosomal protein 97 | PRSS53 | NM_001039503 | protease, serine 53 |
| SKIL | NM_005414 | SKI-like proto-oncogene | ATP8B1 | NM_005603 | ATPase phospholipid transporting 8B1 | C16orf87 | NM_001001436 | chromosome 16 open reading frame 87 |
| MPP7 | NM_173496 | membrane palmitoylated protein 7 | CBWD6 | NM_001085457 | COBW domain containing 6 | PXMP2 | NM_018663 | peroxisomal membrane protein 2 |
| DCUN1D1 | NM_020640 | defective in cullin neddylation 1 domain containing 1 | SERHL | NR_027786 | serine hydrolase-like (pseudogene) | GLCE | NM_015554 | glucuronic acid epimerase |
| SLC11A1 | NM_000578 | solute carrier family 11 member 1 | OSBPL8 | NM_001003712 | Oxysterol-binding protein-like 8 | PLEKHG4 | NM_001129727 | pleckstrin homology and RhoGEF domain containing G4 |
| ZNF365 | NM_014951 | zinc finger protein 365 | CDPF1 | NM_207327 | cysteine rich DPF motif domain containing 1 | CDH3 | NM_001793 | cadherin 3 |
| RPIA | NM_144563 | ribose 5-phosphate isomerase A | ALG12 | NM_024105 | ALG12, alpha-1,6-mannosyltransferase | PHLPP2 | NM_015020 | PH domain and leucine rich repeat protein phosphatase 2 |
| SIPA1L1 | NM_001284245 | Signal-induced proliferation-associated 1-like 1 | CBWD1 | NM_018491 | COBW domain containing 1 | CLDN22 | NM_001111319 | claudin 22 |
| KLHL5 | NM_001171654 | Kelch-like family member 5 | SLC41A1 | NM_173854 | solute carrier family 41 member 1 | ADAT1 | NR_036460 | adenosine deaminase, tRNA specific 1 |
| TYMS | NM_001071 | thymidylate synthetase | SMN2 | NM_022875 | survival of motor neuron 2, centromeric | ZNF469 | NM_001127464 | zinc finger protein 469 |
| ZNF512 | NM_001271289 | zinc finger protein 512 | TRNT1 | NM_001302946 | tRNA nucleotidyl transferase, CCA-adding, 1 | THEM6 | NM_016647 | thioesterase superfamily member 6 |
| PUS7L | NM_031292 | pseudouridylate synthase 7 like | BHLHE40 | NM_003670 | basic helix-loop-helix family member e40 | PIK3R6 | NM_001290211 | phosphoinositide-3-kinase regulatory subunit 6 |
| KIAA0226L | NM_025113 | KIAA0226-like | RAD18 | NM_020165 | RAD18, E3 ubiquitin protein ligase | ZNF624 | NM_020787 | zinc finger protein 624 |
| ZNF182 | NM_001178099 | zinc finger protein 182 | SRGAP3 | NM_014850 | SLIT-ROBO Rho GTPase activating protein 3 | GJA1 | NM_000165 | gap junction protein alpha 1 |
| NR1D2 | NR_110524 | nuclear receptor subfamily 1 group D member 2 | BCL2L11 | NM_006538 | BCL2-like 11 | CCDC144B | NR_036647 | coiled-coil domain containing 144B (pseudogene) |
| OLIG1 | NM_138983 | oligodendrocyte transcription factor 1 | SDE2 | NM_152608 | SDE2 telomere maintenance homolog | EXD2 | NM_018199 | exonuclease 3'-5' domain containing 2 |
| RPL32P3 | NR_003111 | ribosomal protein L32 pseudogene 3 | HDAC11 | NM_024827 | histone deacetylase 11 | TMEM183A | NM_138391 | transmembrane protein 183A |
| HADH | NM_005327 | hydroxyacyl-CoA dehydrogenase | DYX1C1-CCPG1 | NR_037923 | DYX1C1-CCPG1 readthrough (NMD candidate) | LOC100507195 | NR_120458 | uncharacterized LOC100507195 |
| CD58 | NR_026665 | CD58 molecule | KIF3A | NM_007054 | kinesin family member 3A | FIGNL1 | NM_001287496 | fidgetin like 1 |
| UPF2 | NM_015542 | UPF2 regulator of nonsense transcripts homolog (yeast) | CD163 | NM_203416 | CD163 molecule | KRT17 | NM_000422 | keratin 17 |
| LOC644936 | NR_004845 | actin, beta pseudogene | CMTM7 | NM_181472 | CKLF like MARVEL transmembrane domain containing 7 | ZNF17 | NM_006959 | zinc finger protein 17 |
| SETD7 | NM_001306199 | SET domain containing lysine methyltransferase 7 | ACSL3 | NM_203372 | acyl-CoA synthetase long-chain family member 3 | DCAKD | NM_001288654 | dephospho-CoA kinase domain containing |
| RAPGEF2 | NM_014247 | Rap guanine nucleotide exchange factor 2 | EXOC8 | NM_175876 | exocyst complex component 8 | WWC2 | NM_024949 | WW and C2 domain containing 2 |
| CASP3 | NM_004346 | caspase 3, apoptosis-related cysteine peptidase | G0S2 | NM_015714 | G0/G1 switch 2 | SP2 | NM_003110 | Sp2 transcription factor |
| PRIMPOL | NM_001300767 | primase and DNA-directed polymerase | DNAJB9 | NM_012328 | DnaJ heat shock protein family (Hsp40) member B9 | F8 | NM_019863 | coagulation factor VIII |
| DNAJC21 | NM_194283 | DnaJ heat shock protein family (Hsp40) member C21 | ZNF35 | NM_003420 | zinc finger protein 35 | DLX3 | NM_005220 | distal-less homeobox 3 |
| IL6ST | NM_175767 | interleukin 6 signal transducer | DCUN1D2 | NM_001014283 | defective in cullin neddylation 1 domain containing 2 | TMPRSS5 | NM_001288751 | transmembrane protease, serine 5 |
| TRAPPC13 | NM_024941 | trafficking protein particle complex 13 | TMEM158 | NM_015444 | transmembrane protein 158 (gene/pseudogene) | GCLC | NM_001498 | glutamate-cysteine ligase catalytic subunit |
| GTF2H2 | NM_001515 | general transcription factor IIH subunit 2 | DTWD1 | NM_020234 | DTW domain containing 1 | ALG3 | NR_024533 | ALG3, alpha-1,3- mannosyltransferase |
| SCAMP1-AS1 | NR_105014 | SCAMP1 antisense RNA 1 | FAM126B | NM_173822 | family with sequence similarity 126 member B | C1QTNF3-AMACR | NR_037951 | C1QTNF3-AMACR readthrough (NMD candidate) |
| FAM151B | NM_205548 | family with sequence similarity 151 member B | ADAM15 | NM_001261465 | ADAM metallopeptidase domain 15 | USP32P2 | NR_003554 | Ubiquitin-specific peptidase 32 pseudogene 2 |
| SEPT8 | NM_001098811 | septin 8 | KRBOX4 | NM_001129898 | KRAB box domain containing 4 | ENDOV | NM_001164637 | endonuclease V |
| PFKFB3 | NM_001145443 | 6-phosphofructo-2-kinase/fructose-2,6-biphosphatase 3 | AHCTF1P1 | NR_077058 | AT-hook containing transcription factor 1 pseudogene 1 | KLHL35 | NM_001039548 | Kelch-like family member 35 |
| C5orf15 | NM_020199 | chromosome 5 open reading frame 15 | SEMA6A | NM_020796 | semaphorin 6A | C18orf25 | NM_145055 | chromosome 18 open reading frame 25 |
| CDKN2AIPNL | NM_080656 | CDKN2A-interacting protein N-terminal-like | C18orf21 | NM_001201476 | chromosome 18 open reading frame 21 | ZNF532 | NM_018181 | zinc finger protein 532 |
| LINC00649 | NR_038883 | long intergenic non-protein coding RNA 649 | PIGB | NM_004855 | phosphatidylinositol glycan anchor biosynthesis class B | G6PD | NM_001042351 | glucose-6-phosphate dehydrogenase |
| CBFA2T3 | NM_175931 | CBFA2/RUNX1 translocation partner 3 | FAM208A | NM_015224 | family with sequence similarity 208 member A | BSG | NM_198590 | basigin (Ok blood group) |
| GRPEL2 | NM_152407 | GrpE-like 2, mitochondrial | SOCS2 | NM_003877 | suppressor of cytokine signaling 2 | SNORA81 | NR_002989 | small nucleolar RNA, H/ACA box 81 |
| SPARC | NM_001309444 | secreted protein acidic and cysteine rich | TCF19 | NM_007109 | transcription factor 19 | SCN1B | NM_199037 | sodium voltage-gated channel beta subunit 1 |
| PNN | NM_002687 | pinin, desmosome-associated protein | ZNF26 | NM_001256280 | zinc finger protein 26 | SFXN2 | NM_178858 | sideroflexin 2 |
| LSM11 | NM_173491 | LSM11, U7 small nuclear RNA-associated | MINA | NM_001261829 | MYC-induced nuclear antigen | CCDC94 | NM_018074 | coiled-coil domain containing 94 |
| BPGM | NM_001293085 | bisphosphoglycerate mutase | CLVS1 | NM_173519 | clavesin 1 | STXBP2 | NM_006949 | Syntaxin-binding protein 2 |
| SFXN1 | NM_022754 | sideroflexin 1 | TMEM229B | NM_182526 | transmembrane protein 229B | LOC100507642 | NR_108065 | uncharacterized LOC100507642 |
| THOC3 | NM_032361 | THO complex 3 | LOC93622 | NR_015433 | Morf4 family-associated protein 1 like 1 pseudogene | RITA1 | NM_001286215 | RBPJ-interacting and tubulin-associated 1 |
| PRRG4 | NM_024081 | proline rich and Gla domain 4 | KATNA1 | NM_001204076 | katanin catalytic subunit A1 | GCDH | NM_000159 | glutaryl-CoA dehydrogenase |
| PITPNC1 | NM_012417 | phosphatidylinositol transfer protein, cytoplasmic 1 | CCDC109B | NM_017918 | coiled-coil domain containing 109B | CASP14 | NM_012114 | caspase 14 |
| HLA-J | NR_024240_2 | major histocompatibility complex, class I, J (pseudogene) | LONRF1 | NM_152271 | LON peptidase N-terminal domain and ring finger 1 | EPHX3 | NM_024794 | epoxide hydrolase 3 |
| GALT | NM_001258332 | galactose-1-phosphate uridylyltransferase | POGLUT1 | NM_152305 | protein O-glucosyltransferase 1 | NR2C2AP | NM_001300945 | nuclear receptor 2C2-associated protein |
| MPHOSPH9 | NM_022782 | M-phase phosphoprotein 9 | PPM1B | NM_001033557 | protein phosphatase, Mg2+/Mn2+ dependent 1B | TSHZ3 | NM_020856 | teashirt zinc finger homeobox 3 |
| ZNRD1-AS1 | NR_026751_7 |  | FAM86JP | NR_024250 | family with sequence similarity 86 member J, pseudogene | DPY19L3 | NM_001172774 | dpy-19 like 3 (C. elegans) |
| ATAD2B | NM_001242338 | ATPase family, AAA domain containing 2B | SLC41A3 | NM_001008486 | solute carrier family 41 member 3 | GPATCH1 | NM_018025 | G-patch domain containing 1 |
| TMEM55A | NM_018710 | transmembrane protein 55A | SLC2A4RG | NM_020062 | SLC2A4 regulator | ZNF91 | NM_003430 | zinc finger protein 91 |
| LINC00342 | NR_103734 | long intergenic non-protein coding RNA 342 | PTPRJ | NM_001098503 | protein tyrosine phosphatase, receptor type J | ZNF585A | NM_152655 | zinc finger protein 585A |
| ASAP1-IT2 | NR_045385 | ASAP1 intronic transcript 2 | ZNF44 | NM_016264 | zinc finger protein 44 | ITPKC | NM_025194 | inositol-trisphosphate 3-kinase C |
| PANDAR | NR_109836 | promoter of CDKN1A antisense DNA damage activated RNA | ATP2C1 | NM_001199182 | ATPase secretory pathway Ca2+ transporting 1 | PPM1N | NM_001080401 | protein phosphatase, Mg2+/Mn2+ dependent 1N (putative) |
| POLR1C | NM_203290 | polymerase (RNA) I subunit C | NPHP3-ACAD11 | NR_037804 | NPHP3-ACAD11 readthrough (NMD candidate) | RDH13 | NM_138412 | retinol dehydrogenase 13 (all-trans/9-cis) |
| VEGFA | NM_001171625 | vascular endothelial growth factor A | ACAD11 | NM_032169 | acyl-CoA dehydrogenase family member 11 | ZNF470 | NM_001001668 | zinc finger protein 470 |
| MAP3K7 | NM_145331 | mitogen-activated protein kinase kinase kinase 7 | ZSCAN21 | NM_145914 | zinc finger and SCAN domain containing 21 | ZNF71 | NM_021216 | zinc finger protein 71 |
| HACE1 | NR_104424 | HECT domain and ankyrin repeat containing E3 ubiquitin protein ligase 1 | CASKIN2 | NM_020753 | CASK-interacting protein 2 | ZNF772 | NM_001024596 | zinc finger protein 772 |
| SESN1 | NM_001199933 | sestrin 1 | NIN | NM_020921 | ninein | ZNF550 | NM_001277092 | zinc finger protein 550 |
| ZBTB24 | NM_014797 | zinc finger and BTB domain containing 24 | STON1 | NM_006873 | stonin 1 | ZNF134 | NM_003435 | zinc finger protein 134 |
| GTF3C6 | NM_138408 | general transcription factor IIIC subunit 6 | EID2 | NM_153232 | EP300-interacting inhibitor of differentiation 2 | IL3RA | NM_001267713 | interleukin 3 receptor subunit alpha |
| NAMPT | NM_005746 | nicotinamide phosphoribosyltransferase | PXYLP1 | NM_152282 | 2-phosphoxylose phosphatase 1 | ARL13B | NM_001174151 | ADP ribosylation factor like GTPase 13B |
| TRMT11 | NM_001031712 | tRNA methyltransferase 11 homolog | FCGR2B | NM_001002273 | Fc fragment of IgG receptor IIb | SH3YL1 | NR_104225 | SH3 and SYLF domain containing 1 |
| VCL | NM_014000 | vinculin | CP | NR_046371 | ceruloplasmin (ferroxidase) | GDF11 | NM_005811 | growth differentiation factor 11 |
| PLCL2 | NM_001144382 | phospholipase C-like 2 | RFX7 | NM_022841 | regulatory factor X7 | GEN1 | NM_182625 | GEN1, Holliday junction 5' flap endonuclease |
| SLC44A2 | NM_001145056 | solute carrier family 44 member 2 | EXOSC5 | NM_020158 | exosome component 5 | INPP5E | NM_019892 | inositol polyphosphate-5-phosphatase E |
| FBXO30 | NM_032145 | F-box protein 30 | LNPEP | NM_005575 | leucyl/cystinyl aminopeptidase | ABHD1 | NM_032604 | abhydrolase domain containing 1 |
| MAP1A | NM_002373 | microtubule-associated protein 1A | SEC63 | NM_007214 | SEC63 homolog, protein translocation regulator | MGA | NM_001080541 | MGA, MAX dimerization protein |
| AGPAT4 | NM_020133 | 1-acylglycerol-3-phosphate O-acyltransferase 4 | ANKRD36B | NM_025190 | ankyrin repeat domain 36B | LOC105747689 | NR_131917 | uncharacterized LOC105747689 |
| CLEC5A | NM_001301167 | C-type lectin domain family 5 member A | PPP1R12B | NM_001167857 | protein phosphatase 1 regulatory subunit 12B | NEAT1 | NR_028272 | nuclear paraspeckle assembly transcript 1 (non-protein coding) |
| DDX26B | NM_182540 |  | PHC3 | NM_024947 | polyhomeotic homolog 3 | PRR12 | NM_020719 | proline rich 12 |
| SP4 | NM_003112 | Sp4 transcription factor | RRM2B | NM_015713 | ribonucleotide reductase regulatory TP53 inducible subunit M2B | SNORD89 | NR_003070 | small nucleolar RNA, C/D box 89 |
| MOB1B | NM_001244766 | MOB kinase activator 1B | AHCTF1 | NM_015446 | AT-hook containing transcription factor 1 | NMRAL1 | NM_001305141 | NmrA-like family domain containing 1 |
| MEMO1 | NR_126034 | mediator of cell motility 1 | PHKA2-AS1 | NR_029379 | PHKA2 antisense RNA 1 | IMMP1L | NM_001304274 | inner mitochondrial membrane peptidase subunit 1 |
| CXorf21 | NM_025159 | chromosome X open reading frame 21 | RPL22L1 | NM_001099645 | ribosomal protein L22 like 1 | AMMECR1L | NM_001199140 | AMMECR1-like |
| PRG4 | NM_005807 | proteoglycan 4 | CCDC50 | NM_174908 | coiled-coil domain containing 50 | ABAT | NM_020686 | 4-aminobutyrate aminotransferase |
| PCF11 | NM_015885 | PCF11 cleavage and polyadenylation factor subunit | USP3-AS1 | NR_034080 | USP3 antisense RNA 1 | USP37 | NM_020935 | ubiquitin specific peptidase 37 |
| KIAA0754 | NM_015038 | KIAA0754 | MRPL42 | NM_172177 | mitochondrial ribosomal protein L42 | PPM1K | NM_152542 | protein phosphatase, Mg2+/Mn2+ dependent 1K |
| SERPING1 | NM_000062 | serpin family G member 1 | CEP19 | NM_032898 | centrosomal protein 19 | CEACAM5 | NM_001308398 | carcinoembryonic antigen-related cell adhesion molecule 5 |
| SOD2 | NM_001024466 | superoxide dismutase 2, mitochondrial | TEX14 | NM_031272 | testis expressed 14 | LINC01560 | NR_126059 | long intergenic non-protein coding RNA 1560 |
| AIG1 | NM_001286587 | androgen-induced 1 | RAB30 | NM_014488 | RAB30, member RAS oncogene family | TTC21B | NM_024753 | tetratricopeptide repeat domain 21B |
| GRAMD1B | NM_020716 | GRAM domain containing 1B | CD46 | NM_153826 | CD46 molecule | MNAT1 | NM_001177963 | MNAT1, CDK activating kinase assembly factor |
| DLEU7 | NM_198989 | deleted in lymphocytic leukemia, 7 | CRIPAK | NM_175918 | cysteine rich PAK1 inhibitor | KIZ | NM_001163022 | kizuna centrosomal protein |
| C5orf58 | NM_001102609 | chromosome 5 open reading frame 58 | ANKS1B | NM_152788 | ankyrin repeat and sterile alpha motif domain containing 1B | TARBP1 | NM_005646 | TAR (HIV-1) RNA-binding protein 1 |
| NDUFA4 | NM_002489 | NDUFA4, mitochondrial complex-associated | CWC22 | NM_020943 | CWC22 homolog, spliceosome-associated protein | C9orf84 | NM_173521 | chromosome 9 open reading frame 84 |
| JAK2 | NM_004972 | Janus kinase 2 | AFAP1 | NM_198595 | actin filament associated protein 1 | STAG3L3 | NR_040582 | stromal antigen 3-like 3 (pseudogene) |
| IGF2BP3 | NM_006547 | Insulin-like growth factor 2 mRNA-binding protein 3 | SLC30A6 | NM_001193513 | solute carrier family 30 member 6 | IFT57 | NM_018010 | intraflagellar transport 57 |
| SLC30A1 | NM_021194 | solute carrier family 30 member 1 | LIMS2 | NM_001136037 | LIM zinc finger domain containing 2 | LANCL1 | NM_006055 | LanC like 1 |
| LANCL2 | NM_018697 | LanC like 2 | NAPEPLD | NM_198990 | N-acyl phosphatidylethanolamine phospholipase D | BCS1L | NM_001257342 | BCS1 homolog, ubiquinol-cytochrome c reductase complex chaperone |
| SBDS | NM_016038 | SBDS ribosome assembly guanine nucleotide exchange factor | LPAR6 | NM_005767 | lysophosphatidic acid receptor 6 | SGK494 | NM_001174103 | uncharacterized serine/threonine-protein kinase SgK494 |
| RFC2 | NM_181471 | replication factor C subunit 2 | TLR1 | NM_003263 | toll like receptor 1 | LOC101928020 | NR_110291 | uncharacterized LOC101928020 |
| ZP3 | NM_007155 | zona pellucida glycoprotein 3 (sperm receptor) | TRAPPC2 | NM_001011658 | trafficking protein particle complex 2 | CCDC85B | NM_006848 | coiled-coil domain containing 85B |
| FDPSP2 | NR_003262 | farnesyl diphosphate synthase pseudogene 2 | EIF2A | NM_032025 | eukaryotic translation initiation factor 2A | SNORD57 | NR_002738 | small nucleolar RNA, C/D box 57 |
| GSAP | NM_017439 | gamma-secretase activating protein | SPIN4 | NM_001012968 | spindlin family member 4 | CEP135 | NM_025009 | centrosomal protein 135 |
| AMICA1 | NM_153206 |  | N4BP2 | NM_018177 | NEDD4-binding protein 2 | ZFP62 | NM_152283 | ZFP62 zinc finger protein |
| PDGFRA | NM_006206 | platelet-derived growth factor receptor alpha | GNPDA2 | NM_001270881 | glucosamine-6-phosphate deaminase 2 | PDCD4-AS1 | NR_026932 | PDCD4 antisense RNA 1 |
| THAP5 | NM_001287601 | THAP domain containing 5 | CDC14B | NM_003671 | cell division cycle 14B | KIAA2018 | NR_111981 |  |
| CLASP2 | NM_015097 | cytoplasmic linker-associated protein 2 | LOC101927164 | NR_110668 | uncharacterized LOC101927164 | NAPB | NR_104266 | NSF attachment protein beta |
| CHCHD3 | NM_017812 | coiled-coil-helix-coiled-coil-helix domain containing 3 | DCUN1D4 | NM_015115 | defective in cullin neddylation 1 domain containing 4 | TMIGD3 | NM_001081976 | transmembrane and immunoglobulin domain containing 3 |
| SCIMP | NM_001271842 | SLP adaptor and CSK-interacting membrane protein | DANCR | NR_024031 | differentiation antagonizing non-protein coding RNA | IFT52 | NM_001303458 | intraflagellar transport 52 |
| LOC100129148 | NR_033999 | uncharacterized LOC100129148 | LOC100507577 | NR_040677 | uncharacterized LOC100507577 | ZNF780A | NM_001142579 | zinc finger protein 780A |
| LGR4 | NM_018490 | leucine-rich repeat containing G protein-coupled receptor 4 | SHOC2 | NM_007373 | SHOC2 leucine-rich repeat scaffold protein | IFT74 | NM_001099223 | intraflagellar transport 74 |
| ZNF767P | NR_027789 | zinc finger family member 767, pseudogene | TWISTNB | NM_001002926 | TWIST neighbor | RFX2 | NM_134433 | regulatory factor X2 |
| GPR65 | NM_003608 | G protein-coupled receptor 65 | CXCL8 | NM_000584 | C-X-C motif chemokine ligand 8 | IDE | NM_001165946 | Insulin-degrading enzyme |
| ASGR1 | NM_001671 | asialoglycoprotein receptor 1 | MYH11 | NM_001040114 | myosin, heavy chain 11, smooth muscle | TASP1 | NM_017714 | taspase 1 |
| OBSCN | NM_001098623 | obscurin, cytoskeletal calmodulin and titin-interacting RhoGEF | LOC100506860 | NR_109780 | uncharacterized LOC100506860 | LINC00174 | NR_026873 | long intergenic non-protein coding RNA 174 |
| MCPH1 | NM_024596 | microcephalin 1 | KIN | NR_045609 | Kin17 DNA and RNA-binding protein | PRAM1 | NM_032152 | PML-RARA-regulated adaptor molecule 1 |
| MTMR7 | NM_004686 | myotubularin-related protein 7 | SGOL2 | NM_001160046 |  | SMG1P2 | NR_002473_1 | SMG1 pseudogene 2 |
| ARPC1A | NM_001190996 | actin-related protein 2/3 complex subunit 1A | LOC729737 | NR_039983 | uncharacterized LOC729737 | TCEA2 | NM_198723 | transcription elongation factor A2 |
| L3MBTL3 | NM_001007102 | l(3)mbt-like 3 (Drosophila) | KIAA1586 | NM_001286274 | KIAA1586 | IGFBP2 | NM_000597 | Insulin-like growth factor-binding protein 2 |
| PAG1 | NM_018440 | phosphoprotein membrane anchor with glycosphingolipid microdomains 1 | LAMTOR3 | NR_024170 | late endosomal/lysosomal adaptor, MAPK and MTOR activator 3 | GRIK1 | NM_175611 | glutamate ionotropic receptor kainate type subunit 1 |
| IMPA1 | NM_001144879 | inositol monophosphatase 1 | BDH2 | NM_020139 | 3-hydroxybutyrate dehydrogenase, type 2 | DGAT2 | NM_001253891 | diacylglycerol O-acyltransferase 2 |
| POP1 | NM_001145861 | POP1 homolog, ribonuclease P/MRP subunit | POC1B | NM_172240 | POC1 centriolar protein B | LSM14A | NM_015578 | LSM14A mRNA-processing body assembly factor |
| MAL2 | NM_052886 | mal, T-cell differentiation protein 2 (gene/pseudogene) | C11orf30 | NM_020193 |  | ZFX | NM_001178095 | zinc finger protein, X-linked |
| HSPA6 | NM_002155 | heat shock protein family A (Hsp70) member 6 | ZNF543 | NM_213598 | zinc finger protein 543 | GNB1L | NM_053004 | G protein subunit beta 1 like |
| ZFAT | NM_001167583 | zinc finger and AT-hook domain containing | MFSD8 | NM_152778 | major facilitator superfamily domain containing 8 | CDADC1 | NM_030911 | cytidine and dCMP deaminase domain containing 1 |
| DGKA | NM_001345 | diacylglycerol kinase alpha | FLNB | NM_001164317 | filamin B | APOL3 | NM_145641 | apolipoprotein L3 |
| ALMS1 | NM_015120 | ALMS1, centrosome and basal body-associated protein | GAB1 | NM_002039 | GRB2-associated binding protein 1 | C1orf131 | NM_001300830 | chromosome 1 open reading frame 131 |
| DGKD | NM_152879 | diacylglycerol kinase delta | SLC10A7 | NM_001029998 | solute carrier family 10 member 7 | ZNF564 | NM_144976 | zinc finger protein 564 |
| LINC00936 | NR_028138 | long intergenic non-protein coding RNA 936 | FOXN2 | NM_002158 | forkhead box N2 | RFPL1S | NR_002727 | RFPL1 antisense RNA 1 |
| LINC01176 | NR_108081 | long intergenic non-protein coding RNA 1176 | ZNF69 | NM_021915 | zinc finger protein 69 | LOC100379224 | NR_033341 | uncharacterized LOC100379224 |
| TJP2 | NM_001170414 | tight-junction protein 2 | NAF1 | NM_001128931 | nuclear assembly factor 1 ribonucleoprotein | MSL3P1 | NR_024322 | male-specific lethal 3 homolog (Drosophila) pseudogene 1 |
| ZNF675 | NM_138330 | zinc finger protein 675 | TMA16 | NM_018352 | translation machinery-associated 16 homolog | ZNF644 | NM_201269 | zinc finger protein 644 |
| ZNF510 | NM_014930 | zinc finger protein 510 | PWP2 | NM_005049 | PWP2 periodic tryptophan protein homolog (yeast) | OSGEPL1 | NM_022353 | O-sialoglycoprotein endopeptidase-like 1 |
| ENTPD1-AS1 | NR_038444 | ENTPD1 antisense RNA 1 | CENPC | NM_001812 | centromere protein C | PAXBP1 | NR_027873 | PAX3 and PAX7-binding protein 1 |
| FAM206A | NM_017832 | family with sequence similarity 206 member A | CBR4 | NM_032783 | carbonyl reductase 4 | ZNF573 | NM_001172690 | zinc finger protein 573 |
| CDC26 | NM_139286 | cell division cycle 26 | C5orf28 | NM_022483 |  | RAB33B | NM_031296 | RAB33B, member RAS oncogene family |
| CDK5RAP2 | NM_018249 | CDK5 regulatory subunit associated protein 2 | FBXO8 | NM_012180 | F-box protein 8 | TUBGCP6 | NM_020461 | tubulin gamma complex associated protein 6 |
| MYBL2 | NM_001278610 | MYB proto-oncogene-like 2 | RWDD4 | NM_001307922 | RWD domain containing 4 | THBD | NM_000361 | thrombomodulin |
| TBC1D13 | NM_001286772 | TBC1 domain family member 13 | SLC25A4 | NM_001151 | solute carrier family 25 member 4 | PRRT3 | NM_207351 | Proline-rich transmembrane protein 3 |
| TRAF3IP3 | NM_025228 | TRAF3-interacting protein 3 | CYP4V2 | NM_207352 | cytochrome P450 family 4 subfamily V member 2 | ITGB2-AS1 | NR_038315 | ITGB2 antisense RNA 1 |
| FAM166A | NM_001001710 | family with sequence similarity 166 member A | AHRR | NM_020731 | aryl-hydrocarbon receptor repressor | EAF1 | NM_033083 | ELL-associated factor 1 |
| NSMF | NM_001130970 | NMDA receptor synaptonuclear signaling and neuronal migration factor | MRPL36 | NM_032479 | mitochondrial ribosomal protein L36 | IL32 | NM_001012633 | interleukin 32 |
| PNPLA4 | NM_001142389 | patatin-like phospholipase domain containing 4 | NUP155 | NM_153485 | nucleoporin 155kDa | ADM | NM_001124 | adrenomedullin |
| SCML1 | NM_006746 | sex comb on midleg-like 1 (Drosophila) | NANP | NM_152667 | N-acetylneuraminic acid phosphatase | CSPG5 | NM_006574 | chondroitin sulfate proteoglycan 5 |
| RP2 | NM_006915 | retinitis pigmentosa 2 (X-linked recessive) | CWC27 | NM_005869 | CWC27 spliceosome-associated protein homolog | CD52 | NM_001803 | CD52 molecule |
| UBA1 | NM_153280 | ubiquitin-like modifier activating enzyme 1 | SMN1 | NM_022874 | survival of motor neuron 1, telomeric | AMT | NM_001164712 | aminomethyltransferase |
| CCL27 | NM_006664 | C-C motif chemokine ligand 27 | POLK | NM_016218 | polymerase (DNA) kappa | CACNA2D4 | NM_172364 | calcium voltage-gated channel auxiliary subunit alpha2delta 4 |
| RRAGB | NM_016656 | Ras-related GTP-binding B | GTPBP2 | NM_019096 | GTP-binding protein 2 | ICAM4 | NM_001039132 | intercellular adhesion molecule 4 (Landsteiner-Wiener blood group) |
| SIRPB2 | NM_001134836 | signal regulatory protein-beta 2 | PAPD4 | NM_001297744 | PAP-associated domain containing 4 | SLC38A1 | NM_001278388 | solute carrier family 38 member 1 |
| RPS26P11 | NR_002309 | ribosomal protein S26 pseudogene 11 | CKMT2-AS1 | NR_034123 | CKMT2 antisense RNA 1 | PRKAA1 | NM_006251 | protein kinase AMP-activated catalytic subunit alpha 1 |
| ETS1 | NM_001162422 | ETS proto-oncogene 1, transcription factor | CCNH | NM_001239 | cyclin H | FAM214A | NM_019600 | family with sequence similarity 214 member A |
| TCEAL4 | NM_001300901 | transcription elongation factor A-like 4 | RIOK2 | NM_018343 | RIO kinase 2 | ZNF654 | NM_018293 | zinc finger protein 654 |
| C1GALT1C1 | NM_152692 | C1GALT1-specific chaperone 1 | GIN1 | NM_017676 | gypsy retrotransposon integrase 1 | SCAF4 | NM_001145445 | SR-related CTD-associated factor 4 |
| IDH3G | NM_004135 | isocitrate dehydrogenase 3 (NAD(+)) gamma | C12orf76 | NM_207435 | chromosome 12 open reading frame 76 | LOC388242 | NR_002556 | SAGA complex-associated factor 29 pseudogene |
| BIRC7 | NM_022161 | baculoviral IAP repeat containing 7 | ZNF25 | NM_145011 | zinc finger protein 25 | BMP8A | NM_181809 | bone morphogenetic protein 8a |
| TNFRSF4 | NM_003327 | tumor necrosis factor receptor superfamily member 4 | RBFOX2 | NM_014309 | RNA-binding protein, fox-1 homolog 2 | SLC15A2 | NM_021082 | solute carrier family 15 member 2 |
| MRVI1 | NM_001100167 | murine retrovirus integration site 1 homolog | ALAD | NM_000031 | aminolevulinate dehydratase | FAM86HP | NR_024252 | family with sequence similarity 86 member H, pseudogene |
| DEFB1 | NM_005218 | defensin beta 1 | HDAC9 | NM_001204144 | histone deacetylase 9 | ASTE1 | NR_110229 | asteroid homolog 1 (Drosophila) |
| TNFSF15 | NM_005118 | tumor necrosis factor superfamily member 15 | ZNF827 | NM_178835 | zinc finger protein 827 | NUDT16P1 | NR_002949 | nudix hydrolase 16 pseudogene 1 |
| PANK4 | NM_018216 | pantothenate kinase 4 | LYL1 | NM_005583 | lymphoblastic leukemia associated hematopoiesis regulator 1 | ZNF224 | NM_013398 | zinc finger protein 224 |
| CYGB | NM_134268 | cytoglobin | ZNF227 | NM_001289167 | zinc finger protein 227 | NPIPB5 | NM_001135865 | nuclear pore complex-interacting protein family member B5 |
| TMEM114 | NR_110736 | transmembrane protein 114 | PKD2L2 | NM_014386 | polycystin 2 like 2, transient receptor potential cation channel | HELZ | NM_014877 | helicase with zinc finger |
| SMIM1 | NM_001288583 | small integral membrane protein 1 (Vel blood group) | PCSK7 | NM_004716 | proprotein convertase subtilisin/kexin type 7 | RPGR | NM_000328 | retinitis pigmentosa GTPase regulator |
| UCHL1 | NM_004181 | ubiquitin C-terminal hydrolase L1 | PRKCZ | NM_002744 | protein kinase C zeta | BRI3BP | NM_080626 | BRI3-binding protein |
| AFAP1L1 | NM_001146337 | actin filament-associated protein 1 like 1 | AIMP1 | NM_001142415 | aminoacyl tRNA synthetase complex-interacting multifunctional protein 1 | CHRD | NM_001304474 | chordin |
| ZMIZ1-AS1 | NR_024429 | ZMIZ1 antisense RNA 1 | TERC | NR_001566 | telomerase RNA component | LOC101926963 | NR_110054 | uncharacterized LOC101926963 |
| C15orf48 | NM_197955 | chromosome 15 open reading frame 48 | ECSCR | NR_121659 | endothelial cell surface expressed chemotaxis and apoptosis regulator | LINC01184 | NR_015360 | long intergenic non-protein coding RNA 1184 |
| CPE | NM_001873 | carboxypeptidase E | PURA | NM_005859 | purine-rich element-binding protein A | RNF4 | NM_002938 | ring finger protein 4 |
| SPON2 | NM_001199021 | spondin 2 | DCAF4L1 | NM_001029955 | DDB1 and CUL4-associated factor 4-like 1 | CHML | NM_001821 | choroideremia-like (Rab escort protein 2) |
| CASP9 | NR_102732 | caspase 9, apoptosis-related cysteine peptidase | IFI27 | NM_001288958 | interferon alpha-inducible protein 27 | LOC100506076 | NR_103732 | uncharacterized LOC100506076 |
| FUT11 | NM_001284194 | fucosyltransferase 11 | FAM35DP | NR_027634 | family with sequence similarity 35 member D, pseudogene | ZNF680 | NM_178558 | zinc finger protein 680 |
| FLJ37453 | NR_024279 | uncharacterized LOC729614 | PCDH12 | NM_016580 | protocadherin 12 | SLC2A9 | NM_001001290 | solute carrier family 2 member 9 |
| SNORA64 | NR_002326 | small nucleolar RNA, H/ACA box 64 | PRELID2 | NM_138492 | PRELI domain containing 2 | MAGI3 | NM_152900 | Membrane-associated guanylate kinase, WW and PDZ domain containing 3 |
| HES2 | NM_019089 | hes family bHLH transcription factor 2 | CCNA2 | NM_001237 | cyclin A2 | STK36 | NM_001243313 | serine/threonine kinase 36 |
| MT1G | NM_001301267 | metallothionein 1G | SPECC1L-ADORA2A | NR_103546 | SPECC1L-ADORA2A readthrough (NMD candidate) | PARP11 | NR_104461 | poly(ADP-ribose) polymerase family member 11 |
| SIGLEC16 | NR_002825 | sialic acid-binding Ig-like lectin 16 (gene/pseudogene) | SP140 | NM_007237 | SP140 nuclear body protein | SGCB | NM_000232 | sarcoglycan, beta (43kDa dystrophin-associated glycoprotein) |
| RARRES1 | NM_206963 | retinoic acid receptor responder 1 | MFAP3 | NM_005927 | microfibrillar-associated protein 3 | MLKL | NM_152649 | mixed lineage kinase domain-like |
| ACP5 | NM_001111036 | acid phosphatase 5, tartrate resistant | RPS6KA5 | NM_004755 | ribosomal protein S6 kinase A5 | RNF139-AS1 | NR_108047 | RNF139 antisense RNA 1 (head to head) |
| TEAD3 | NM_003214 | TEA domain transcription factor 3 | THUMPD2 | NM_025264 | THUMP domain containing 2 | FCMR | NM_001193338 | Fc fragment of IgM receptor |
| KANK3 | NM_198471 | KN motif and ankyrin repeat domains 3 | TTC9 | NM_015351 | tetratricopeptide repeat domain 9 | NOA1 | NM_032313 | nitric oxide-associated 1 |
| ARFGEF3 | NM_020340 | ARFGEF family member 3 | POLR3C | NM_001303456 | polymerase (RNA) III subunit C | CXCL1 | NM_001511 | C-X-C motif chemokine ligand 1 |
| ATP2B2 | NM_001683 | ATPase plasma membrane Ca2+ transporting 2 | LIPE | NM_005357 | lipase E, hormone sensitive type | PIAS1 | NM_016166 | protein inhibitor of activated STAT 1 |
| HTRA4 | NM_153692 | HtrA serine peptidase 4 | USP15 | NM_001252079 | ubiquitin-specific peptidase 15 | CXCL2 | NM_002089 | C-X-C motif chemokine ligand 2 |
| ALOX15B | NM_001141 | arachidonate 15-lipoxygenase, type B | ADAMTSL4 | NM_019032 | ADAMTS like 4 | FAM47E-STBD1 | NM_001242939 | FAM47E-STBD1 readthrough |
| PDIK1L | NM_001243532 | PDLIM1-interacting kinase 1 like | ADAT2 | NM_182503 | adenosine deaminase, tRNA specific 2 | HAUS5 | NM_015302 | HAUS augmin-like complex subunit 5 |
| ANGPTL4 | NR_104213 | angiopoietin like 4 | ZNF805 | NM_001145078 | zinc finger protein 805 | TBC1D22A-AS1 | NR_122047 | TBC1D22A antisense RNA 1 |
| MYO1B | NM_001130158 | myosin IB | CALD1 | NM_033138 | caldesmon 1 | ZMAT1 | NM_001011657 | zinc finger matrin-type 1 |
| COL1A2 | NM_000089 | collagen type I alpha 2 | SCARNA22 | NR_003004 | small Cajal body-specific RNA 22 | GSTCD | NM_024751 | glutathione S-transferase C-terminal domain containing |
| CLGN | NM_004362 | calmegin | LTC4S | NM_145867 | leukotriene C4 synthase | RNASE4 | NM_001282193 | ribonuclease A family member 4 |
| ZNF620 | NM_175888 | zinc finger protein 620 | MTRNR2L4 | NM_001190476 | MT-RNR2-like 4 | TLR4 | NM_003266 | Toll-like receptor 4 |
| BCAT1 | NM_001178094 | branched chain amino acid transaminase 1 | TTLL11 | NM_001139442 | tubulin tyrosine ligase-like 11 | ZNF33A | NM_001278177 | zinc finger protein 33A |
| TXLNGY | NR_045128 | taxilin gamma pseudogene, Y-linked | TRIM52-AS1 | NR_102759 | TRIM52 antisense RNA 1 (head to head) | HMGB3 | NM_001301231 | High-mobility group box 3 |
| SNRNP40 | NM_004814 | small nuclear ribonucleoprotein U5 subunit 40 | FOXC1 | NM_001453 | forkhead box C1 | LSM6 | NM_007080 | LSM6 homolog, U6 small nuclear RNA and mRNA degradation-associated |
| CCDC28B | NM_001301011 | coiled-coil domain containing 28B | MMP14 | NM_004995 | matrix metallopeptidase 14 | TMEM184C | NM_018241 | transmembrane protein 184C |
| ZBTB8OS | NM_178547 | zinc finger and BTB domain containing 8 opposite strand | PSMG4 | NM_001128591 | proteasome assembly chaperone 4 | CFAP44 | NM_001164496 | cilia and flagella-associated protein 44 |
| RNF19B | NM_153341 | ring finger protein 19B | SLC16A6 | NM_001174166 | solute carrier family 16 member 6 | C4orf46 | NR_077235 | chromosome 4 open reading frame 46 |
| AZIN2 | NM_001293562 | antizyme inhibitor 2 | SNRNP48 | NM_152551 | small nuclear ribonucleoprotein U11/U12 subunit 48 | LOC731424 | NR_037867 |  |
| USP9Y | NM_004654 | ubiquitin-specific peptidase 9, Y-linked | KIAA0101 | NR_109934 | KIAA0101 | GUSBP1 | NR_027028 | glucuronidase, beta pseudogene 1 |
| EVA1B | NM_018166 | eva-1 homolog B (*C. elegans*) | LOC103611081 | NR_125382 | uncharacterized LOC103611081 | CCDC112 | NM_152549 | coiled-coil domain containing 112 |
| NLRP2 | NM_001174081 | NLR family, pyrin domain containing 2 | ADAM8 | NM_001109 | ADAM metallopeptidase domain 8 | RAD50 | NM_005732 | RAD50 double-strand break repair protein |
| F3 | NM_001178096 | coagulation factor III, tissue factor | NUP153 | NM_001278210 | nucleoporin 153kDa | CCNI2 | NM_001039780 | cyclin I family member 2 |
| DUSP13 | NM_001007273 | dual-specificity phosphatase 13 | ELOVL3 | NM_152310 | ELOVL fatty acid elongase 3 | C5orf66 | NM_001277348 | chromosome 5 open reading frame 66 |
| SVBP | NM_199342 | small vasohibin-binding protein | KATNBL1P6 | NR_003954 | katanin regulatory subunit B1 like 1 pseudogene 6 | C1orf228 | NM_001145636 | chromosome 1 open reading frame 228 |
| RRAD | NM_001128850 | Ras-related associated with diabetes | HIST1H4C | NM_003542 | histone cluster 1, H4c | FRY | NM_023037 | FRY microtubule-binding protein |
| CLDN3 | NM_001306 | claudin 3 | SMC5 | NM_015110 | structural maintenance of chromosomes 5 | APBB3 | NM_006051 | amyloid beta precursor protein-binding family B member 3 |
| PTGDS | NM_000954 | prostaglandin D2 synthase | VAV3 | NM_006113 | vav guanine nucleotide exchange factor 3 | TMCO6 | NM_001300982 | transmembrane and coiled-coil domains 6 |
| HRH1 | NM_001098213 | histamine receptor H1 | ATF7IP | NM_001286515 | activating transcription factor 7-interacting protein | C1orf162 | NM_001300835 | chromosome 1 open reading frame 162 |
| TESK2 | NM_007170 | testis-specific kinase 2 | TTPAL | NM_024331 | tocopherol (alpha) transfer protein-like | C1S | NM_001734 | complement component 1, s subcomponent |
| NPTX2 | NM_002523 | neuronal pentraxin 2 | DXO | NM_005510 | decapping exoribonuclease | RBM7 | NM_016090 | RNA-binding motif protein 7 |
| ADM2 | NM_024866 | adrenomedullin 2 | IER3 | NM_003897_4 | immediate early response 3 | GUSBP4 | NR_003660 | glucuronidase, beta pseudogene 4 |
| KTI12 | NM_138417 | KTI12 chromatin-associated homolog | CDSN | NM_001264 | corneodesmosin | IL24 | NM_001185156 | interleukin 24 |
| GNG12 | NM_018841 | G protein subunit gamma 12 | BTN2A1 | NM_007049 | butyrophilin subfamily 2 member A1 | GEMIN5 | NM_001252156 | gem nuclear organelle associated protein 5 |
| CD109 | NM_001159587 | CD109 molecule | GAB3 | NM_080612 | GRB2-associated binding protein 3 | FAM102A | NM_203305 | family with sequence similarity 102 member A |
| JCHAIN | NM_144646 | joining chain of multimeric IgA and IgM | LPCAT4 | NM_153613 | lysophosphatidylcholine acyltransferase 4 | EZH2 | NM_152998 | enhancer of zeste homolog 2 (Drosophila) |
| NPR1 | NM_000906 | natriuretic peptide receptor 1 | SS18L1 | NM_198935 | SS18L1, nBAF chromatin remodeling complex subunit | AATK | NM_004920 | apoptosis-associated tyrosine kinase |
| HRK | NR_073189 | harakiri, BCL2-interacting protein | NCR3 | NM_147130 | natural cytotoxicity triggering receptor 3 | LOC101928445 | NR_110560 | uncharacterized LOC101928445 |
| NEURL3 | NM_001285485 | neuralized E3 ubiquitin protein ligase 3 | HSD17B8 | NM_014234_5 | hydroxysteroid (17-beta) dehydrogenase 8 | PLA2G4B | NM_001114633 | phospholipase A2 group IVB |
| PARVA | NM_018222 | parvin alpha | RSPH3 | NM_031924 | radial spoke 3 homolog | ZBTB20 | NM_015642 | zinc finger and BTB domain containing 20 |
| PRCD | NR_033357 | progressive rod-cone degeneration | SNTB1 | NM_021021 | syntrophin beta 1 | RNF144B | NM_182757 | ring finger protein 144B |
| FPGT | NM_001199329 | fucose-1-phosphate guanylyltransferase | CCDC167 | NM_138493 | coiled-coil domain containing 167 | GMNN | NM_001251990 | geminin, DNA replication inhibitor |
| CRYZ | NM_001889 | crystallin zeta | LOC728554 | NR_003615 | THO complex 3 pseudogene | GABPB1-AS1 | NR_024490 | GABPB1 antisense RNA 1 |
| PIGK | NM_005482 | phosphatidylinositol glycan anchor biosynthesis class K | ELOVL5 | NM_021814 | ELOVL fatty acid elongase 5 | ZSCAN9 | NM_001199480 | zinc finger and SCAN domain containing 9 |
| SPTBN2 | NM_006946 | spectrin beta, non-erythrocytic 2 | BAG2 | NM_004282 | BCL2 associated athanogene 2 | ZNF789 | NM_001013258 | zinc finger protein 789 |
| CALB2 | NM_007088 | calbindin 2 | LIN54 | NM_194282 | lin-54 DREAM MuvB core complex component | TBC1D10C | NM_001256508 | TBC1 domain family member 10C |
| ARHGAP44 | NM_014859 | Rho GTPase-activating protein 44 | FAM135A | NM_020819 | family with sequence similarity 135 member A | ZNF721 | NM_133474 | zinc finger protein 721 |
| KLK10 | NM_001077500 | kallikrein-related peptidase 10 | VNN1 | NM_004666 | vanin 1 | KIFC1 | NM_002263 | kinesin family member C1 |
| MYBPH | NM_004997 | myosin-binding protein H | EEF1A1 | NM_001402 | eukaryotic translation elongation factor 1 alpha 1 | ITPR3 | NM_002224 | inositol 1,4,5-trisphosphate receptor type 3 |
| CCDC18 | NM_001306076 | coiled-coil domain containing 18 | APH1B | NM_031301 | aph-1 homolog B, gamma secretase subunit | ENPP4 | NM_014936 | ectonucleotide pyrophosphatase/phosphodiesterase 4 (putative) |
| GSG1 | NM_001206842 | germ cell associated 1 | TRAF3IP2-AS1 | NR_034108 | TRAF3IP2 antisense RNA 1 | SH2D3C | NM_170600 | SH2 domain containing 3C |
| ACTR3B | NM_001040135 | ARP3 actin-related protein 3 homolog B (yeast) | AURKB | NM_001284526 | aurora kinase B | KLHDC1 | NM_172193 | kelch domain containing 1 |
| LOC100288911 | NR_037631 | uncharacterized LOC100288911 | MAFB | NM_005461 | v-maf avian musculoaponeurotic fibrosarcoma oncogene homolog B | CD40 | NM_001302753 | CD40 molecule |
| MMP10 | NM_002425 | matrix metallopeptidase 10 | GOLGA8B | NR_027410 | golgin A8 family member B | LY6G5C | NM_025262 | lymphocyte antigen 6 complex, locus G5C |
| FAM102B | NM_001010883 | family with sequence similarity 102 member B | ZUFSP | NM_145062 | zinc finger with UFM1-specific peptidase domain | LTV1 | NM_032860 | LTV1 ribosome biogenesis factor |
| CD300E | NM_181449 | CD300e molecule | TTLL3 | NM_001025930 | tubulin tyrosine ligase like 3 | PLAGL1 | NM_001080956 | PLAG1-like zinc finger 1 |
| CD180 | NM_005582 | CD180 molecule | RNF217 | NM_001286398 | ring finger protein 217 | UST | NM_005715 | uronyl 2-sulfotransferase |
| CYB561D1 | NM_001134402 | cytochrome b561 family member D1 | THAP9-AS1 | NR_034075 | THAP9 antisense RNA 1 | LGALS9C | NM_001040078 | lectin, galactoside-binding soluble 9C |
| PIPOX | NM_016518 | pipecolic acid and sarcosine oxidase | LAMA2 | NM_000426 | laminin subunit alpha 2 | ARMT1 | NM_001286562 | acidic residue methyltransferase 1 |
| ZNF653 | NM_138783 | zinc finger protein 653 | NLRP12 | NM_001277129 | NLR family, pyrin domain containing 12 | RALGPS2 | NM_001286247 | Ral GEF with PH domain and SH3-binding motif 2 |
| VANGL1 | NM_138959 | VANGL planar cell polarity protein 1 | SCARF1 | NR_102409 | scavenger receptor class F member 1 | LGALS2 | NM_006498 | lectin, galactoside-binding soluble 2 |
| SLC22A15 | NM_018420 | solute carrier family 22 member 15 | LOC100507557 | NR_038246 | uncharacterized LOC100507557 | CLEC1B | NM_001099431 | C-type lectin domain family 1 member B |
| WBP5 | NM_001006612 |  | RAB32 | NM_006834 | RAB32, member RAS oncogene family | KIF21B | NM_001252103 | kinesin family member 21B |
| COL5A3 | NM_015719 | collagen type V alpha 3 | RABGAP1L | NM_014857 | RAB GTPase activating protein 1-like | NLRP3 | NM_001079821 | NLR family, pyrin domain containing 3 |
| PYGO1 | NM_015617 | pygopus family PHD finger 1 | TIFA | NM_052864 | TRAF-interacting protein with forkhead associated domain | CDT1 | NM_030928 | chromatin licensing and DNA replication factor 1 |
| WARS2 | NM_015836 | tryptophanyl tRNA synthetase 2, mitochondrial | MTRF1L | NM_001301871 | mitochondrial translational release factor 1 like | GSTT2 | NR_126445 | glutathione S-transferase theta 2 (gene/pseudogene) |
| GPX8 | NR_131338 | glutathione peroxidase 8 (putative) | PTPN7 | NR_037663 | protein tyrosine phosphatase, non-receptor type 7 | CCL5 | NM_002985 | C-C motif chemokine ligand 5 |
| CST6 | NM_001323 | cystatin E/M | ZNF718 | NR_110527 | zinc finger protein 718 | PLSCR1 | NM_021105 | phospholipid scramblase 1 |
| TYRO3 | NM_006293 | TYRO3 protein tyrosine kinase | DOCK11 | NM_144658 | dedicator of cytokinesis 11 | SEMA4B | NM_020210 | semaphorin 4B |
| TMEM53 | NM_001300748 | transmembrane protein 53 | ZNF671 | NM_024833 | zinc finger protein 671 | REPS2 | NM_004726 | RALBP1-associated Eps domain containing 2 |
| CERS2 | NM_022075 | ceramide synthase 2 | LOC729603 | NR_003288 | calcineurin-like EF-hand protein 1 pseudogene | CES1 | NM_001266 | carboxylesterase 1 |
| GBAT2 | NR_131972 | glioblastoma multiforme-associated transcript 2 | FAM216A | NM_013300 | family with sequence similarity 216 member A | GADD45B | NM_015675 | growth arrest and DNA damage inducible beta |
| ARHGEF17 | NM_014786 | Rho guanine nucleotide exchange factor 17 | UBE2C | NM_181800 | ubiquitin conjugating enzyme E2 C | WDR76 | NM_024908 | WD repeat domain 76 |
| MRPL9 | NM_001300733 | mitochondrial ribosomal protein L9 | PAQR3 | NM_001040202 | progestin and adipoQ receptor family member 3 | ZDBF2 | NM_020923 | zinc finger DBF-type containing 2 |
| SNORD49A | NR_002744 | small nucleolar RNA, C/D box 49A | GP1BA | NM_000173 | glycoprotein Ib platelet alpha subunit | NAP1L5 | NM_153757 | nucleosome assembly protein 1 like 5 |
| CRCT1 | NM_019060 | cysteine rich C-terminal 1 | SLC8A1-AS1 | NR_038441 | SLC8A1 antisense RNA 1 | ULK2 | NM_001142610 | unc-51 like autophagy activating kinase 2 |
| SPRR2G | NM_001014291 | small proline-rich protein 2G | ARL5B | NM_178815 | ADP ribosylation factor-like GTPase 5B | LOC100130744 | NR_046285 | uncharacterized LOC100130744 |
| S100A7A | NM_176823 | S100 calcium-binding protein A7A | C4orf33 | NM_173487 | chromosome 4 open reading frame 33 | HIBADH | NM_152740 | 3-hydroxyisobutyrate dehydrogenase |
| LY9 | NM_002348 | lymphocyte antigen 9 | DKFZP586I1420 | NR_002186 | uncharacterized protein DKFZp586I1420 | ZNF717 | NM_001290210 | zinc finger protein 717 |
| ME3 | NM_006680 | malic enzyme 3 | ZNF37BP | NR_026777 | zinc finger protein 37B, pseudogene | CWF19L2 | NM_152434 | CWF19-like 2, cell cycle control (S. pombe) |
| SNAR-B1 | NR_024231 | small ILF3/NF90-associated RNA B1 | LST1 | NM_205839 | leukocyte specific transcript 1 | RP9P | NR_003500 | retinitis pigmentosa 9 pseudogene |
| GPD1 | NM_005276 | glycerol-3-phosphate dehydrogenase 1 | TRIM66 | NM_014818 | tripartite motif containing 66 | NPIPB4 | NM_001310148 | nuclear pore complex-interacting protein family member B4 |
| RAB17 | NR_033308 | RAB17, member RAS oncogene family | MRS2P2 | NR_024072 | MRS2 pseudogene 2 | SAP25 | NM_001168682 | Sin3A-associated protein 25kDa |
| SYDE1 | NM_033025 | synapse defective Rho GTPase homolog 1 | COL17A1 | NM_000494 | collagen type XVII alpha 1 | CCL23 | NM_005064 | C-C motif chemokine ligand 23 |
| SLC6A9 | NM_001261380 | solute carrier family 6 member 9 | B4GALT4 | NM_212543 | beta-1,4-galactosyltransferase 4 | NPIPB3 | NM_130464 | nuclear pore complex-interacting protein family member B3 |
| ZBTB7B | NR_045515 | zinc finger and BTB domain containing 7B | LILRB1 | NM_006669 | leukocyte immunoglobulin like receptor B1 | TSPAN32 | NM_139022 | tetraspanin 32 |
| MSRB1 | NM_016332 | methionine sulfoxide reductase B1 | GVINP1 | NR_003945 | GTPase, very large interferon inducible pseudogene 1 | KLF7 | NM_003709 | Kruppel-like factor 7 (ubiquitous) |
| SCARNA18 | NR_003139 | small Cajal body-specific RNA 18 | SNAPC1 | NM_003082 | small nuclear RNA activating complex polypeptide 1 | ITIH4 | NM_001166449 | inter-alpha-trypsin inhibitor heavy chain family member 4 |
| PMF1 | NM_001199654 | polyamine-modulated factor 1 | ZNF101 | NM_001300949 | zinc finger protein 101 | ID1 | NM_002165 | inhibitor of DNA binding 1, HLH protein |
| GDF1 | NM_001492 | growth differentiation factor 1 | BRWD3 | NM_153252 | bromodomain and WD repeat domain containing 3 | ANXA2R | NM_001014279 | annexin A2 receptor |
| FANCD2 | NM_033084 | Fanconi anemia complementation group D2 | GPR132 | NM_001278696 | G protein-coupled receptor 132 | TSPAN9 | NM_001168320 | tetraspanin 9 |
| C2orf71 | NM_001029883 | chromosome 2 open reading frame 71 | NFIX | NM_002501 | nuclear factor I X | MLLT11 | NM_006818 | myeloid/lymphoid or mixed-lineage leukemia |
| CRTAC1 | NM_018058 | cartilage acidic protein 1 | UMAD1 | NM_001302350 | UBAP1-MVB12-associated (UMA) domain containing 1 | AHCYL2 | NM_015328 | adenosylhomocysteinase like 2 |
| MPZ | NM_000530 | myelin protein zero | KLHL7 | NM_001031710 | Kelch-like family member 7 | TMEM209 | NM_001301163 | transmembrane protein 209 |
| EGFL7 | NR_046367 | EGF-like domain multiple 7 | MPP6 | NM_016447 | membrane palmitoylated protein 6 | SEMA4C | NM_017789 | semaphorin 4C |
| GPRASP2 | NM_001004051 | G protein-coupled receptor associated sorting protein 2 | HOXA5 | NM_019102 | homeobox A5 | CELF2-AS1 | NR_126062 | CELF2 antisense RNA 1 |
| SCUBE2 | NM_001170690 | signal peptide, CUB domain and EGF like domain containing 2 | HOXA10-AS | NR_046609 | HOXA10 antisense RNA | TCAF1 | NM_001206938 | TRPM8 channel-associated factor 1 |
| TPD52L1 | NM_001300994 | tumor protein D52-like 1 | BORA | NM_024808 | bora, aurora kinase A activator | GJB6 | NM_001110221 | gap junction protein beta 6 |
| CENPO | NM_001199803 | centromere protein O | MARCKSL1 | NM_023009 | MARCKS-like 1 | ATF7IP2 | NM_024997 | activating transcription factor 7-interacting protein 2 |
| ZFY | NM_001145276 | zinc finger protein, Y-linked | KYNU | NM_001199241 | kynureninase | LMBR1 | NM_022458 | limb development membrane protein 1 |
| ZC3H12C | NM_033390 | zinc finger CCCH-type containing 12C | POLM | NR_104299 | polymerase (DNA) mu | FUT10 | NM_032664 | fucosyltransferase 10 |
| APOBEC3G | NM_021822 | apolipoprotein B mRNA-editing enzyme catalytic subunit 3G | CD36 | NM_001001547 | CD36 molecule | OSM | NM_020530 | oncostatin M |
| USP12-AS2 | NR_046548 | USP12 antisense RNA 2 (head to head) | TAF9B | NM_015975 | TATA-box binding protein associated factor 9b | PTCRA | NM_001243169 | pre T-cell antigen receptor alpha |
| MT1L | NR_001447 | metallothionein 1L (gene/pseudogene) | TPST1 | NM_003596 | tyrosylprotein sulfotransferase 1 | EPHB4 | NM_004444 | EPH receptor B4 |
| PLS3 | NM_005032 | plastin 3 | GS1-124K5.11 | NR_111973 | RAB guanine nucleotide exchange factor 1 pseudogene | GLCCI1 | NM_138426 | Glucocorticoid-induced 1 |
| FAM95B1 | NR_026759 | family with sequence similarity 95 member B1 | SBDSP1 | NR_001588 | Shwachman-Bodian-Diamond syndrome pseudogene 1 | GIMAP2 | NM_015660 | GTPase, IMAP family member 2 |
| RGS1 | NM_002922 | regulator of G-protein signaling 1 | ABHD11 | NM_001301058 | abhydrolase domain containing 11 | RAD54B | NM_012415 | RAD54 homolog B (S. cerevisiae) |
| DENND1B | NM_001195215 | DENN domain containing 1B | CSF1 | NM_172210 | colony-stimulating factor 1 | TMC8 | NM_152468 | transmembrane channel like 8 |
| ZFYVE16 | NM_001284236 | zinc finger FYVE-type containing 16 | PTPN12 | NM_002835 | protein tyrosine phosphatase, non-receptor type 12 | LAPTM4B | NM_018407 | lysosomal protein transmembrane 4 beta |
| LY6K | NM_017527 | lymphocyte antigen 6 complex, locus K | CARD11 | NM_032415 | caspase recruitment domain family member 11 | CD28 | NM_001243077 | CD28 molecule |
| S100A14 | NM_020672 | S100 calcium-binding protein A14 | MTURN | NM_152793 | maturin, neural progenitor differentiation regulator homolog (Xenopus) | WDYHV1 | NM_001283027 | WDYHV motif containing 1 |
| ARNTL2 | NM_001248004 | aryl hydrocarbon receptor nuclear translocator like 2 | GSTT1 | NM_001293809 | glutathione S-transferase theta 1 | HRH2 | NM_001131055 | histamine receptor H2 |
| CROCCP2 | NR_026752 | ciliary rootlet coiled-coil, rootletin pseudogene 2 | GRAP | NM_006613 | GRB2-related adaptor protein | LY6D | NM_003695 | lymphocyte antigen 6 complex, locus D |
| TRIM14 | NM_033219 | tripartite motif containing 14 | DBF4 | NM_006716 | DBF4 zinc finger | PRKCH | NM_006255 | protein kinase C eta |
| ZNF626 | NM_001076675 | zinc finger protein 626 | ROGDI | NR_046480 | rogdi homolog | SIGLEC14 | NM_001098612 | sialic acid-binding Ig-like lectin 14 |
| HPGD | NM_000860 | hydroxyprostaglandin dehydrogenase 15-(NAD) | CPVL | NM_019029 | carboxypeptidase, vitellogenic like | TTC39B | NM_001168341 | tetratricopeptide repeat domain 39B |
| C16orf93 | NM_001014979 |  | VPS50 | NM_024553 | VPS50, EARP/GARPII complex subunit | SKP2 | NM_001243120 | S-phase kinase-associated protein 2, E3 ubiquitin protein ligase |
| SMAD6 | NR_027654 | SMAD family member 6 | DUSP2 | NM_004418 | dual-specificity phosphatase 2 | NDUFB6 | NM_002493 | NADH:ubiquinone oxidoreductase subunit B6 |
| ERRFI1 | NM_018948 | ERBB receptor feedback inhibitor 1 | TMEM130 | NM_001134451 | transmembrane protein 130 | RND3 | NM_001254738 | Rho family GTPase 3 |
| KIAA1456 | NM_020844 | KIAA1456 | TRRAP | NM_001244580 | transformation/transcription domain-associated protein | FAM26F | NM_001276460 | family with sequence similarity 26 member F |
| KCNJ1 | NM_000220 | potassium voltage-gated channel subfamily J member 1 | FAM200A | NM_145111 | family with sequence similarity 200 member A | EMB | NM_198449 | embigin |
| USP30-AS1 | NR_038996 | USP30 antisense RNA 1 | ZNF3 | NM_017715 | zinc finger protein 3 | ABLIM1 | NM_006720 | Actin-binding LIM protein 1 |
| HLA-DPA1 | NM_001242525_1 | major histocompatibility complex, class II, DP alpha 1 | MOSPD3 | NM_001040097 | motile sperm domain containing 3 | C9orf85 | NM_182505 | chromosome 9 open reading frame 85 |
| SLC7A11-AS1 | NR_038380 | SLC7A11 antisense RNA 1 | MKKS | NR_072977 | McKusick-Kaufman syndrome | PRUNE2 | NR_131751 | prune homolog 2 (Drosophila) |
| SLC47A1 | NM_018242 | solute carrier family 47 member 1 | CDKN2A | NM_000077 | cyclin-dependent kinase inhibitor 2A | GRK5 | NM_005308 | G protein-coupled receptor kinase 5 |
| LPGAT1 | NM_014873 | lysophosphatidylglycerol acyltransferase 1 | ENC1 | NM_001256576 | ectodermal-neural cortex 1 | IDNK | NR_046422 | idnK, gluconokinase homolog (E. coli) |
| MAMSTR | NM_001297753 | MEF2-activating motif and SAP domain containing transcriptional regulator | LSM8 | NM_016200 | LSM8 homolog, U6 small nuclear RNA associated | CCDC88C | NM_001080414 | coiled-coil domain containing 88C |
| ECE2 | NM_014693 | Endothelin-converting enzyme 2 | CHUK | NM_001278 | conserved helix-loop-helix ubiquitous kinase | CFL2 | NM_021914 | cofilin 2 |
| LOC100289561 | NM_001242713 | uncharacterized LOC100289561 | METTL2B | NM_018396 | methyltransferase like 2B | MAML3 | NM_018717 | mastermind like transcriptional coactivator 3 |
| ARHGDIB | NM_001175 | Rho GDP dissociation inhibitor beta | ZNF107 | NM_016220 | zinc finger protein 107 | SCAI | NM_173690 | suppressor of cancer cell invasion |
| AMIGO2 | NM_181847 | adhesion molecule with Ig-like domain 2 | SPIRE1 | NM_001128626 | spire-type actin nucleation factor 1 | GCH1 | NM_001024070 | GTP cyclohydrolase 1 |
| FES | NM_001143785 | FES proto-oncogene, tyrosine kinase | MTRNR2L6 | NM_001190487 | MT-RNR2-like 6 | FAM78A | NM_033387 | family with sequence similarity 78 member A |
| MYO10 | NM_012334 | myosin X | CELSR1 | NM_014246 | cadherin EGF LAG seven-pass G-type receptor 1 | GTF3C4 | NM_012204 | general transcription factor IIIC subunit 4 |
| FAM89A | NM_198552 | family with sequence similarity 89 member A | AP1AR | NM_001128426 | adaptor-related protein complex 1-associated regulatory protein | RALGDS | NM_001271776 | ral guanine nucleotide dissociation stimulator |
| PCNXL2 | NM_014801 |  | CUL1 | NM_003592 | cullin 1 | SURF2 | NM_001278928 | surfeit 2 |
| RTKN | NM_001015056 | rhotekin | LINC00996 | NR_034033 | long intergenic non-protein coding RNA 996 | DPH3P1 | NM_080750 | diphthamide biosynthesis 3 pseudogene 1 |
| TRIM26 | NM_001242783_5 | tripartite motif containing 26 | PLK2 | NM_001252226 | Polo-like kinase 2 | ZNF674 | NM_001146291 | zinc finger protein 674 |
| MEF2C | NM_001193349 | myocyte enhancer factor 2C | CSGALNACT1 | NR_024040 | chondroitin sulfate N-acetylgalactosaminyltransferase 1 | GIMAP6 | NM_001244071 | GTPase, IMAP family member 6 |
| DESI2 | NM_016076 | desumoylating isopeptidase 2 | FABP5P3 | NR_002935 | fatty acid-binding protein 5 pseudogene 3 | GIMAP8 | NM_175571 | GTPase, IMAP family member 8 |
| AMPD3 | NM_001172431 | adenosine monophosphate deaminase 3 | FFAR4 | NM_181745 | free fatty acid receptor 4 | BRAF | NM_004333 | B-Raf proto-oncogene, serine/threonine kinase |
| RAB40B | NM_006822 | RAB40B, member RAS oncogene family | LPAR5 | NM_001142961 | lysophosphatidic acid receptor 5 | CD1C | NM_001765 | CD1c molecule |
| PODXL | NM_001018111 | podocalyxin-like | RBPMS2 | NM_194272 | RNA-binding protein with multiple splicing 2 | ARL4C | NM_001282431 | ADP ribosylation factor like GTPase 4C |
| PCGF2 | NM_007144 | polycomb group ring finger 2 | ATP8B2 | NM_001005855 | ATPase phospholipid transporting 8B2 | ZNF462 | NM_021224 | zinc finger protein 462 |
| ACTRT3 | NM_032487 | actin-related protein T3 | ANGPT2 | NM_001147 | angiopoietin 2 | PPP1R3F | NM_001184745 | protein phosphatase 1 regulatory subunit 3F |
| ECM1 | NM_022664 | extracellular matrix protein 1 | SGK223 | NM_001080826 | homolog of rat pragma of Rnd2 | ZNF567 | NM_001300979 | zinc finger protein 567 |
| FAM101B | NM_182705 | family with sequence similarity 101 member B | C10orf35 | NM_145306 | chromosome 10 open reading frame 35 | HMGN5 | NM_030763 | high-mobility group nucleosome binding domain 5 |
| RHOBTB1 | NR_024555 | Rho-related BTB domain containing 1 | KIF20B | NM_016195 | kinesin family member 20B | GIMAP7 | NM_153236 | GTPase, IMAP family member 7 |
| LOC100499489 | NR_036533 | uncharacterized LOC100499489 | TRABD2A | NM_001277053 | TraB domain containing 2A | FCGR3B | NM_001244753 | Fc fragment of IgG receptor IIIb |
| MTPAP | NM_018109 | mitochondrial poly(A) polymerase | GFRA2 | NM_001495 | GDNF family receptor alpha 2 | BAIAP2L1 | NM_018842 | BAI1-associated protein 2 like 1 |
| GBP5 | NM_001134486 | guanylate-binding protein 5 | WASF1 | NM_003931 | WAS protein family member 1 | HPRT1 | NM_000194 | hypoxanthine phosphoribosyltransferase 1 |
| DNAJB5 | NM_012266 | DnaJ heat shock protein family (Hsp40) member B5 | SCARNA17 | NR_003003 | small Cajal body-specific RNA 17 | FAM65B | NM_014722 | family with sequence similarity 65 member B |
| XPOT | NM_007235 | exportin for tRNA | RAP1GAP2 | NM_001100398 | RAP1 GTPase activating protein 2 | CD160 | NM_007053 | CD160 molecule |
| FAM45A | NM_001303111 | family with sequence similarity 45 member A | HORMAD2-AS1 | NR_110541 | HORMAD2 antisense RNA 1 | PVRL3 | NM_001243286 |  |

**Supplemental Table 7. GO analysis of 2,559 genes significantly altered in ALS(R)-iMGs compared to ALS(S)-iMGs**

| **Category** | **Term** | **Count** | **Genes** |
| --- | --- | --- | --- |
| GOTERM_BP_DIRECT | GO:0006935~chemotaxis | 30 | CXCL1, CCL2, CXCL5, ENPP2, C5, CXCL2, FPR1, CXCL8, FES, CCL5, CCL27, CCL7, S1PR1, CCL23, RALA, DEFB1, C5AR2, AIMP1, CMKLR1, CCL18, CCL13, CXCL14, CCR5, ECSCR, LTB4R2, CMTM8, CMTM7, ANOS1, PTGDR2, PLAU |
| GOTERM_BP_DIRECT | GO:0042384~cilium assembly | 29 | ABLIM1, CLUAP1, STK36, NEK1, TMEM216, DYNC2LI1, TTLL3, WDR19, TMEM231, IFT122, TTBK2, MKKS, EXOC5, CCDC28B, IFT140, KIF3A, RPGR, ALMS1, CBY1, BBS1, TRAF3IP1, POC1B, FNBP1L, IFT57, RAB17, RFX3, RFX2, IFT74, ARL13B |
| GOTERM_BP_DIRECT | GO:0035338~long-chain fatty-acyl-CoA biosynthetic process | 13 | ACOT7, ACSL1, ELOVL5, ELOVL3, PPT2, SLC25A1, HACD1, ELOVL6, ACSL4, ACSL3, TECR, ACSF2, ACOT4 |
| GOTERM_BP_DIRECT | GO:0051056~regulation of small GTPase-mediated signal transduction | 27 | DLC1, RAP1GAP, RALGAPB, MYO9A, ARHGAP12, STARD13, ARHGAP6, ARHGAP5, ARHGAP44, SOS1, RHOBTB2, RHOBTB1, ARHGAP11A, RAP1GAP2, INPP5B, RHOF, ARHGDIB, OBSCN, VAV3, ARHGEF16, ARHGEF17, ARHGEF9, ARHGAP23, SYDE1, SIPA1L1, SRGAP3, SRGAP1 |
| GOTERM_BP_DIRECT | GO:0006355~regulation of transcription, DNA-templated | 210 | MEF2C, SLC2A4RG, CDKN2AIPNL, HMGN5, ZNF251, ZSCAN9, ZNF772, ZNF107, PITX1, ZNF101, ZNF44, ZNF644, TFPT, ZNF506, ZNF234, ZNF789, ZNF384, ST18, TRAPPC2, ZNF510, ZNF232, AHCTF1, ZNF230, ZNF618, ZNF512, AHRR, ZNF227, SCAND2P, LYL1, ZNF224, PIR, PBRM1, ZNF71, ARNTL2, ZNF69, IKZF5, ZNF624, ZBTB47, ZNF620, SMAD3, PMF1, ZNF626, ZNF497, ZSCAN2, ZNF585A, SHOX2, DLX3, CAPN15, RNF6, TULP3, ZNF211, RAD54B, KAT6B, ZFHX2, ZNF219, BACH1, ZNF17, TAF1A, ZNF532, TAF1D, EZH2, ZNF675, ZNF674, ZNF680, ZNF146, ZNF33A, BRF1, SNAPC2, SNAPC1, ZNF543, SCAI, ZNF688, ZBTB24, GTF2H2, ZNF140, ZNF341, ZNF134, ZNF747, FOXC1, ZNF550, LIN54, ZNF746, ZNF555, ZFP62, ZNF469, CARHSP1, ZBTB6, THAP5, SCML1, ZNF75A, ZNF35, WTIP, ZNF654, ZNF780A, ZNF653, SUFU, ZNF75D, LPXN, NR1D2, ZSCAN21, ZNF850, ZSCAN25, ZNF286A, PRKAA1, USP34, ZNF561, ZNF470, ZNF566, ZNF264, ZNF28, ZNF567, NLK, ZNF569, ZNF160, ZFP1, PHF10, ZNF25, ZNF26, ZNF668, WWTR1, MSRB2, RPS6KA5, MNAT1, ZNF672, IFT57, ZSCAN32, ZNF761, APBB3, ZNF573, ZNF584, ZNF827, ZXDB, PNN, KDM1B, GTF2E1, USP27X, ZNF182, ZNF579, OLIG1, ZNF574, MLXIPL, HDAC10, ZNF816, HDAC11, ZNF3, SP140, KRBA2, ZNF195, MGA, ZNF436, ZNF845, HMGB3, TRRAP, NR2C1, CXXC1, ZNF322, ZNF597, BHLHE40, ZNF420, KAT8, EEF1A1, KAT7, TAF5, RFX7, ATAD2, HACE1, ATF7IP2, FAM208A, MSL3, EAF1, ZNF317, ZNF417, PNRC2, HOXB6, HIVEP2, RFX3, RFX2, E2F1, ZNF83, ZNF200, SAP25, PCGF3, LBH, GTF2A1, GTF2A2, PAX8, ZNF721, INO80E, KRBOX4, INO80C, KHDRBS3, ZFY, ZFX, ZNF91, ZNF718, ZNF714, NCOA5, ZNF717, ZNF805, NFYB, MACC1, ZNF708, NFATC2, ZBTB7B, ZNF818P, L3MBTL3, MAK, ADNP2, GMCL1, SETD7 |
| GOTERM_BP_DIRECT | GO:0032496~response to lipopolysaccharide | 31 | CXCL1, TNFRSF6B, ALAD, CXCL5, CXCL3, CXCL2, SNCA, ACP5, TLR4, GNG12, TNFRSF4, GCH1, SLC11A1, IRAK3, CASP3, PTGIR, CYP27B1, CASP9, LIAS, CHUK, CD40, SPARC, GJB6, PCK2, S100A14, SOD2, TNFRSF10A, THBD, PPBP, ADM, JAK2 |
| GOTERM_BP_DIRECT | GO:0006954~inflammatory response | 61 | LY86, TLR1, TLR4, IL10, PTGIR, SEMA7A, IL1RAP, VNN1, ADAM8, CHUK, IRAK2, C5AR2, GBP5, CHST2, CD40, IL24, NLRP3, NLRP2, NCR3, TNFRSF10A, TNFAIP6, KLRG1, CCR5, PPBP, TNFRSF6B, CXCL1, TPST1, PRKCZ, CCL2, CXCL5, CXCL3, CSF1, C5, CXCL2, FPR1, CXCL8, CCL5, TNFRSF4, CCL7, SLC11A1, HRH1, CCL23, FOLR2, PSTPIP1, BCL6, LIAS, PTX3, BLNK, CSF1R, OLR1, AIMP1, ECM1, CD180, CCL18, RPS6KA5, LAT, APOL3, CCL13, LTB4R2, HDAC9, PLA2G4B |
| GOTERM_BP_DIRECT | GO:0030041~actin filament polymerization | 8 | COBL, ARHGAP6, WASF1, MSRB1, JAK2, WAS, TTC17, MSRB2 |
| GOTERM_BP_DIRECT | GO:0006955~immune response | 63 | AQP9, TLR1, TNFSF15, TNFSF14, TLR4, C1QC, IL10, SEMA7A, IL1RAP, FCGR3B, CHUK, CMKLR1, PRG4, CD40, IL24, CD164, WAS, NCR3, OSM, TNFRSF10A, CD36, PPBP, CCR5, CST7, HLA-DPA1, MAP3K14, PTGDR2, TNFRSF6B, CXCL1, IL1R2, CCL2, LST1, HLA-DRB1, CXCL5, ENPP2, CXCL3, CXCL2, JCHAIN, GPR65, CXCL8, IL32, OAS1, CCL5, TNFRSF4, CCL27, SLC11A1, CCL23, HRH2, HLA-DOA, DEFB1, SMAD6, IL1RN, SMAD3, CCL18, LAT, CCL13, RGS1, FCGR2B, CXCL14, SP2, ETS1, CD274, TGFBR3 |
| GOTERM_BP_DIRECT | GO:0008152~metabolic process | 28 | SGSH, BCAT1, ARSD, AQP9, ARSK, ACAT1, ACSF2, MUT, ACSL1, ACSL4, AGPAT4, DXO, ACSL3, ACAA2, CES1, SUCLG2, ISOC2, LPCAT2, MAN1C1, LPCAT4, TMEM68, CPE, HDHD3, CENPV, ACAD10, LIPE, ACSM5, GSTP1 |
| GOTERM_BP_DIRECT | GO:0015937~coenzyme A biosynthetic process | 6 | PANK4, COASY, ACOT7, PPCDC, ACAT1, DCAKD |
| GOTERM_BP_DIRECT | GO:0030890~positive regulation of B cell proliferation | 12 | MEF2C, CARD11, VAV3, SLC39A10, BST1, BCL2, BCL6, ATAD5, TLR4, CD40, NFATC2, TNFRSF4 |
| GOTERM_BP_DIRECT | GO:0060271~cilium morphogenesis | 28 | STK36, NEK1, TMEM216, TTLL3, WDR19, TMEM231, IFT122, TTBK2, MKKS, IFT43, CCDC28B, IFT140, KIF3A, RAB8B, RPGR, TTC21B, WWTR1, PTPDC1, BBS1, POC1B, IFT57, RAB17, RFX3, RFX2, IFT52, PARVA, IFT74, ARL13B |
| GOTERM_BP_DIRECT | GO:0060907~positive regulation of macrophage cytokine production | 5 | CD36, SEMA7A, TLR4, SPON2, GPRC5B |
| GOTERM_BP_DIRECT | GO:0070232~regulation of T cell apoptotic process | 4 | LGALS3, GIMAP8, CD274, JAK3 |
| GOTERM_BP_DIRECT | GO:0031100~organ regeneration | 13 | CDK1, UPF2, CCL2, PPARG, TGFBR2, PPAT, GAS6, ADM, IGF2R, TGFBR3, ANGPT2, CCNA2, GSTP1 |
| GOTERM_BP_DIRECT | GO:0006974~cellular response to DNA damage stimulus | 38 | ARMT1, BLM, XIAP, TIPIN, KIAA0101, CHEK1, KIN, CHCHD6, FANCL, NPAS2, CHD1L, CASP9, APITD1-CORT, BCL2, WDR76, BCL6, NFATC2, TOP2A, TAF1, VAV3, SMC5, ATAD5, MACROD1, ATR, RAD52, UBE2B, RAD50, BRCA1, TANK, C9ORF142, TIMELESS, CUL4A, UBA1, SMARCAL1, SUSD6, RAD18, SETD7, PIDD1 |
| GOTERM_BP_DIRECT | GO:0001843~neural tube closure | 18 | DVL2, DLC1, COBL, CLUAP1, CECR2, CELSR1, SUFU, MTHFD1, IFT122, TULP3, ADM, IFT57, ST14, SEMA4C, KIF20B, RALA, GLMN, LIAS |
| GOTERM_BP_DIRECT | GO:0030168~platelet activation | 24 | PRKCA, GNA13, TYRO3, VAV3, C1GALT1C1, F8, PRKCH, CD40, ITPR3, GAS6, DGKA, CD9, PLSCR1, LAT, VWF, DGKD, FYN, P2RY1, COL1A2, GP1BA, ADRA2B, COL1A1, PIK3R6, CLEC1B |
| GOTERM_BP_DIRECT | GO:0009411~response to UV | 12 | MAP4K3, CDKN2D, USP1, TRIM32, PRIMPOL, TIPIN, KIAA0101, RAD18, PRKAA1, SERPINB13, UBE2B, GTF2H2 |
| GOTERM_BP_DIRECT | GO:0006260~DNA replication | 30 | BLM, DBF4, TIPIN, KIAA0101, NAP1L1, NFIX, CHEK1, KIN, CDT1, IGHMBP2, INO80E, RECQL4, CDC6, CDK1, POLK, KAT7, POLH, NOL8, IGF1, TREX1, ATR, BRCA1, RAD50, MCM5, RFC5, RFC3, TIMELESS, RFC1, RFC2, CHTF18 |
| GOTERM_BP_DIRECT | GO:0006351~transcription, DNA-templated | 267 | MEF2C, SLC2A4RG, FSTL3, HMGN5, CITED4, ZNF251, MAP3K7, MAMSTR, CRY2, ZSCAN9, ZNF772, ZNF107, ZNF101, ZNF44, ZNF644, TFPT, ZNF506, MTPAP, ZNF234, ZNF789, RFC1, HES2, ZNF384, ST18, PIAS1, MAD2L2, TRAPPC2, ERBB2, ZNF510, CHCHD3, ZNF232, ZNF230, ZNF618, ZNF512, PLAGL1, ZNF227, AHRR, ZNF224, LYL1, PBRM1, ZNF71, ARNTL2, NKRF, ZNF69, IKZF5, AEBP2, KLF7, ZNF624, ZBTB47, ZNF620, SMAD6, SMAD3, ZNF497, ZNF626, ZSCAN2, ZNF585A, RNF4, ZNF211, TCF19, RAD54B, ZFHX2, KAT6B, ZNF219, ZNF17, ZBTB33, ZNF532, PPARG, EZH2, ZNF675, ZNF674, GLI1, ZNF680, SCX, ATF7IP, ZNF281, ZBTB20, ZNF33A, CCNH, ZNF543, SCAI, POLR1C, POLR1B, ZNF688, ZNF140, ZBTB24, GTF2H2, ZNF341, ZNF134, ZNF550, LIN54, ZNF746, ZNF555, POLR2G, ZFP62, ZNF469, ZBTB6, THAP5, SCML1, ZNF366, ZNF75A, ZNF35, WTIP, ZNF654, SEC14L2, ZNF780A, ZNF653, ZNF174, ZNF75D, LPXN, CIR1, ZSCAN21, NR1D2, ZNF850, PER2, ZNF286A, ZSCAN25, TCEA1, BCL6, PRKAA1, TCEA2, ZNF561, ZNF470, BRMS1L, ZNF564, ZNF566, ZNF264, ZNF28, ZNF567, NLK, ZNF569, ZNF160, ZFP1, PHF10, ZNF25, ZNF26, ZNF668, POLR3C, ZNF671, SNAI3, ZNF672, IFT57, ZSCAN32, ZNF761, ZNF462, ZMYND15, ZNF573, ZNF584, CCDC85B, ZNF451, ZNF827, INO80, ZXDB, TCEAL4, PNN, KDM1B, IGHMBP2, ZNF182, CDKN2A, MIER1, CGGBP1, ZNF579, OLIG1, ZNF574, SS18L1, MLXIPL, HDAC10, FOXN2, ZNF816, HDAC11, ARID1B, SPOCD1, POLR2J2, ZNF3, POLR2J3, TRIM37, ZNF195, TIMELESS, TRIM33, MGA, NFE2L3, ZNF436, ZNF845, TSHZ3, EID2, HMGB3, TRRAP, NR2C1, CXXC1, ZNF322, OVOL1, ZNF597, BHLHE40, ZNF420, SLC30A9, KAT8, EEF1A1, ZMYM2, KAT7, HACE1, ATAD2, RNPS1, TEAD3, MYPOP, BRCA1, ATF7IP2, FAM208A, ATF6, MSL3, EAF1, MSL3P1, ZNF317, ZNF417, PNRC2, HOXB6, RFX3, RFX2, HDAC9, E2F1, ZNF83, E2F6, ZNF200, ZNF202, SAP25, CBFA2T3, PCGF2, FUBP3, PCGF3, LBH, PAX8, MKL2, ZNF721, INO80E, INO80C, KHDRBS3, ZFY, ZFX, ZNF91, NLRP3, PURA, MURC, ZNF718, ZNF714, NCOA5, ZNF717, MYRF, PEG3, ZNF805, NFYB, NFIX, PAXBP1, MACC1, MINA, NPAS2, ZNF708, GTF3C6, CHD6, GTF3C4, CEBPA, ZNF818P, L3MBTL3, MAK, ADNP2, ID2, SP2, ID1, SP4, SETD7 |
| GOTERM_BP_DIRECT | GO:2000505~regulation of energy homeostasis | 6 | CD36, NR1D2, LEPR, MLXIPL, PRKAA1, BMP8A |
| GOTERM_BP_DIRECT | GO:0032695~negative regulation of interleukin-12 production | 6 | IRAK3, CMKLR1, ACP5, PIBF1, JAK3, IL10 |
| GOTERM_BP_DIRECT | GO:2001224~positive regulation of neuron migration | 5 | SEMA6A, KIF20B, NSMF, SEMA3A, RAPGEF2 |
| GOTERM_BP_DIRECT | GO:0008544~epidermis development | 19 | KLK7, KIF3A, SPRR2G, CDSN, WAS, LAMB3, COL17A1, CST6, KRT5, KRT17, SPRR2D, CASP14, CTGF, ATP2C1, GAB1, OVOL1, SPRR3, CALML5, FABP5 |
| GOTERM_BP_DIRECT | GO:0014070~response to organic cyclic compound | 13 | TAF2, NAMPT, CDK1, TRPA1, ACACB, MMP14, ACAT1, G6PD, ACSL1, PLIN2, ABCD3, ANGPT2, ACSL3 |
| GOTERM_BP_DIRECT | GO:0001568~blood vessel development | 11 | MEF2C, DLX3, MIB1, ALDH1A2, EGFL7, SPHK2, DHCR7, TGFBR2, COL1A2, COL1A1, RAPGEF2 |
| GOTERM_BP_DIRECT | GO:0006361~transcription initiation from RNA polymerase I promoter | 10 | MNAT1, TAF1A, POLR2L, RRN3, CCNH, TAF1D, POLR1C, POLR1B, TWISTNB, GTF2H2 |
| GOTERM_BP_DIRECT | GO:0051770~positive regulation of nitric-oxide synthase biosynthetic process | 6 | NAMPT, NOD2, CCL2, FCER2, TLR4, JAK2 |
| GOTERM_BP_DIRECT | GO:0033591~response to L-ascorbic acid | 4 | ITGA2, SPARC, GSTP1, SOD2 |
| GOTERM_BP_DIRECT | GO:0043589~skin morphogenesis | 5 | COL1A2, ITGA2, COL1A1, ERRFI1, CDSN |
| GOTERM_BP_DIRECT | GO:0048245~eosinophil chemotaxis | 5 | CCL13, HRH1, LGALS3, CCL5, CCL7 |
| GOTERM_BP_DIRECT | GO:0030574~collagen catabolic process | 15 | COL4A1, ADAMTS14, MMP19, MRC2, MMP8, MMP15, COL5A3, MMP14, MMP12, MMP10, COL1A2, COL6A2, COL6A1, COL1A1, ADAM15 |
| GOTERM_BP_DIRECT | GO:0042276~error-prone translesion synthesis | 7 | RFC5, POLK, RFC3, RFC1, RFC2, MAD2L2, REV3L |
| GOTERM_BP_DIRECT | GO:0009083~branched-chain amino acid catabolic process | 7 | BCAT1, ACADSB, BCKDK, PPM1K, HIBCH, ACAT1, HIBADH |
| GOTERM_BP_DIRECT | GO:0042572~retinol metabolic process | 9 | ALDH1A1, ALDH1A2, DHRS3, DHRS4, DGAT2, DHRS9, PNPLA4, RDH13, RETSAT |
| GOTERM_BP_DIRECT | GO:0006362~transcription elongation from RNA polymerase I promoter | 9 | MNAT1, TAF1A, POLR2L, CCNH, TAF1D, POLR1C, POLR1B, TWISTNB, GTF2H2 |
| GOTERM_BP_DIRECT | GO:0019985~translesion synthesis | 10 | RFC5, POLK, RFC3, POLH, RFC1, ISG15, RFC2, PRIMPOL, KIAA0101, REV3L |
| GOTERM_BP_DIRECT | GO:0090307~mitotic spindle assembly | 10 | TUBGCP4, KIFC1, TUBGCP6, TUBGCP5, XIAP, FAM175B, BIRC7, MZT1, PIBF1, MYBL2 |
| GOTERM_BP_DIRECT | GO:0051289~protein homotetramerization | 14 | GBP5, SYT11, TRPA1, IDE, CBR4, ACACB, DECR1, HPRT1, CRYZ, PPAT, TK1, SOD2, CTH, APIP |
| GOTERM_BP_DIRECT | GO:0035721~intraciliary retrograde transport | 5 | WDR19, IFT122, TTC21B, IFT43, IFT140 |
| GOTERM_BP_DIRECT | GO:0042787~protein ubiquitination involved in ubiquitin-dependent protein catabolic process | 28 | FZR1, ABTB1, UBA6, RNF217, AURKB, KLHL7, ARIH1, C18ORF25, C19ORF68, BTBD1, KLHL28, BTBD2, RNF122, CUL1, RNF144B, TAF1, CDK1, HERC6, HACE1, HERC4, CDC26, UBE2C, CUL4A, TRIM32, ANAPC7, ASB2, RNF19B, FBXO10 |
| GOTERM_BP_DIRECT | GO:0006928~movement of cell or subcellular component | 18 | GNA13, ENPP2, WASF1, CALD1, CXCL8, IGF1, CERCAM, VCL, CD9, DNALI1, IGSF8, VNN1, HSPB1, ANOS1, KPTN, JAK2, ARHGDIB, TUBB4B |
| GOTERM_BP_DIRECT | GO:0043547~positive regulation of GTPase activity | 84 | DLC1, RAB3GAP2, RP2, EZH2, ARHGAP12, PTGIR, ARHGAP6, TBC1D17, RASA4B, S1PR1, ARHGAP5, GRIN2D, ARHGAP11A, RAPGEF2, DOCK11, ERRFI1, CSF2RA, DENND6B, DENND6A, ARHGEF1, RPGR, MADD, ARHGEF5, ARHGEF17, ARHGEF9, CD40, ARHGAP23, SH2D3C, ACAP3, ARFGEF3, FNBP1L, PDGFRA, SRGAP3, RIN1, TRAPPC1, SRGAP1, RALGPS2, CCL2, RAP1GAP, ERBB2, CCL5, DENND2D, ARFGEF1, MYO9A, CCL7, STARD13, ALDH1A1, AGAP11, CCL23, PLEKHG7, ARHGAP44, SOS1, CHM, CAMK2B, RAP1GAP2, CCZ1, INPP5B, RASA4, FGD6, FBXO8, ARHGDIB, DVL2, OBSCN, LAMTOR3, VAV3, SPTBN4, NF1, RGS18, CCL18, RALGDS, DOCK4, DENND1B, LAT, CCL13, RABEP2, RGS1, CHML, FYN, SPTBN2, JAK2, JAK3, ST5, IL3RA, GFRA2 |
| GOTERM_BP_DIRECT | GO:0006363~termination of RNA polymerase I transcription | 9 | MNAT1, TAF1A, POLR2L, CCNH, TAF1D, POLR1C, POLR1B, TWISTNB, GTF2H2 |
| GOTERM_BP_DIRECT | GO:0051415~interphase microtubule nucleation by interphase microtubule organizing center | 4 | TUBGCP4, TUBGCP6, TUBGCP5, MZT1 |
| GOTERM_BP_DIRECT | GO:0046600~negative regulation of centriole replication | 4 | TRIM37, CDK5RAP2, BRCA1, MDM1 |
| GOTERM_BP_DIRECT | GO:0001947~heart looping | 14 | MEF2C, CLUAP1, SETDB2, KIF3A, TGFBR2, GJA1, SMAD3, SUFU, MIB1, IFT57, PKD2, MKKS, IFT52, ARL13B |
| GOTERM_BP_DIRECT | GO:0006488~dolichol-linked oligosaccharide biosynthetic process | 6 | ALG10B, ALG3, SRD5A3, ALG10, ALG12, ALG13 |
| GOTERM_BP_DIRECT | GO:0030488~tRNA methylation | 8 | TARBP1, TRMT11, TRMT13, HENMT1, THUMPD2, KIAA1456, METTL2B, FTSJ1 |
| GOTERM_BP_DIRECT | GO:0006281~DNA repair | 39 | BACH1, FZR1, ERCC6L2, BLM, ENDOV, CDC14B, UNG, INO80, CHEK1, TRRAP, RRM2B, KIN, IGHMBP2, FANCL, CHD1L, APITD1-CORT, POLM, INO80E, INO80C, RECQL4, POLK, CDK1, TFPT, POLH, GEN1, PIF1, TREX1, SMG1, ATR, RAD52, UBE2B, RAD50, BRCA1, RFC5, MNAT1, FAAP100, FANCD2, RAD18, ASTE1 |
| GOTERM_BP_DIRECT | GO:0006635~fatty acid beta-oxidation | 11 | ACAA2, ACADS, ABCD1, ABCD3, BDH2, DECR1, HIBCH, ACAD11, HADH, ACAD10, ACAT1 |
| GOTERM_BP_DIRECT | GO:0000082~G1/S transition of mitotic cell cycle | 20 | BCAT1, CDC6, CDK1, CCNH, DBF4, SKP2, GPR132, PPAT, MCM5, CDT1, TYMS, MNAT1, CDKN2A, CUL4A, PLK2, CDKN2D, USP37, PIAS1, CCNA1, CUL1 |
| GOTERM_BP_DIRECT | GO:0050927~positive regulation of positive chemotaxis | 5 | S1PR1, F3, VEGFA, SMAD3, ITGA2 |
| GOTERM_BP_DIRECT | GO:0051895~negative regulation of focal adhesion assembly | 6 | DLC1, ARHGAP6, RCC2, APOD, CLASP2, MMP14 |
| GOTERM_BP_DIRECT | GO:0051642~centrosome localization | 6 | SEMA6A, ASUN, KIF5B, NIN, PLXNA2, RANBP2 |
| GOTERM_BP_DIRECT | GO:0007099~centriole replication | 6 | SASS6, CCP110, CEP135, WDR62, CDK5RAP2, CENPJ |
| GOTERM_BP_DIRECT | GO:0006919~activation of cysteine-type endopeptidase activity involved in apoptotic process | 17 | DLC1, PPARG, SNCA, TNFSF15, SMAD3, NLRP3, BCL2L11, TNFRSF10A, IFI27, CASP3, CDKN2A, F3, IFT57, NLRP12, JAK2, PIDD1, EIF2AK3 |
| GOTERM_BP_DIRECT | GO:0034447~very-low-density lipoprotein particle clearance | 3 | APOE, APOC1, VLDLR |
| GOTERM_BP_DIRECT | GO:0010642~negative regulation of platelet-derived growth factor receptor signaling pathway | 4 | PTPRJ, PTGIR, APOD, SNCA |
| GOTERM_BP_DIRECT | GO:0033135~regulation of peptidyl-serine phosphorylation | 4 | PLCL2, PLCL1, SPTBN4, PRKAA1 |
| GOTERM_BP_DIRECT | GO:1900264~positive regulation of DNA-directed DNA polymerase activity | 4 | RFC5, RFC3, RFC2, CHTF18 |
| GOTERM_BP_DIRECT | GO:0060326~cell chemotaxis | 14 | CXCL1, CCL2, CXCL5, BCAR1, C5, CXCL2, ARHGEF16, FPR1, CCL5, CCL27, DOCK4, CCL13, CXCL14, PDGFRA |
| GOTERM_BP_DIRECT | GO:0030224~monocyte differentiation | 6 | MEF2C, PIR, VEGFA, PPARG, MT1G, CSF1R |
| GOTERM_BP_DIRECT | GO:0043651~linoleic acid metabolic process | 6 | ACSL1, ELOVL5, ALOX15B, ABCD1, ELOVL3, GSTP1 |
| GOTERM_BP_DIRECT | GO:0009435~NAD biosynthetic process | 5 | NAMPT, KYNU, QPRT, NMRK2, NAPRT |
| GOTERM_BP_DIRECT | GO:0006164~purine nucleotide biosynthetic process | 5 | MTHFD1, OAS1, HPRT1, PFAS, PPAT |
| GOTERM_BP_DIRECT | GO:0097502~mannosylation | 5 | PIGZ, PIGV, ALG3, PIGB, ALG12 |
| GOTERM_BP_DIRECT | GO:0000083~regulation of transcription involved in G1/S transition of mitotic cell cycle | 7 | BACH1, CDK1, CDC6, TYMS, E2F6, CCNA1, CDT1 |
| GOTERM_BP_DIRECT | GO:0070527~platelet aggregation | 10 | TYRO3, TSPAN32, PDGFRA, STXBP1, HSPB1, GP1BA, HBB, GAS6, MYL9, VCL |
| GOTERM_BP_DIRECT | GO:0006654~phosphatidic acid biosynthetic process | 9 | DGKA, GPD1L, PLD2, GPD1, DDHD1, GNPAT, AGPAT4, PLA2G4B, LPCAT4 |
| GOTERM_BP_DIRECT | GO:0006283~transcription-coupled nucleotide-excision repair | 15 | POLR2G, COPS2, POLK, POLR2L, CCNH, COPS7A, GTF2H2, RFC5, MNAT1, RFC3, AQR, RFC1, CUL4A, RFC2, TCEA1 |
| GOTERM_BP_DIRECT | GO:0001666~response to hypoxia | 29 | ALAD, CCL2, AHCY, CLDN3, WTIP, CBFA2T3, CASP3, HMOX1, PRKAA1, CYGB, ANGPT2, ANGPTL4, KCNMA1, ACTN4, TGFBR2, NF1, CST3, SMAD3, ITGA2, MMP14, SOD2, SCFD1, ADM, ETS1, VEGFA, PKLR, ABAT, TGFBR3, PLAU |
| GOTERM_BP_DIRECT | GO:0030225~macrophage differentiation | 6 | CEBPA, GAB3, L3MBTL3, CSF1, VEGFA, CSF1R |
| GOTERM_BP_DIRECT | GO:0051301~cell division | 53 | DYNC1LI1, KIFC1, SEPT4, MAD1L1, FAM175B, BCAR1, BORA, INO80, TTC28, CD2AP, CCNA1, CCNA2, TUBA1B, CDK14, CDC6, CDK1, CCNF, DYNLT3, UBE2C, MCM5, NCAPD2, TIMELESS, RCC2, ZWINT, PELO, ANAPC7, MAD2L2, FZR1, HAUS5, ASUN, NEK1, TIPIN, CDC73, VRK1, NCAPH, APITD1-CORT, NCAPG, KATNA1, CENPC, USP37, CLASP2, LMLN, PARD6B, SETDB2, SMC5, PMF1, CDC26, CENPJ, SMC4, TEX14, ANXA11, CENPV, KIF20B |
| GOTERM_BP_DIRECT | GO:0042254~ribosome biogenesis | 9 | MINA, NAF1, RRN3, GTPBP10, MRPL36, RSL24D1, NOA1, BOP1, GNL2 |
| GOTERM_BP_DIRECT | GO:0043407~negative regulation of MAP kinase activity | 9 | PTPRJ, IRAK3, SPRY1, APOE, NF1, UCHL1, SORL1, PAQR3, GSTP1 |
| GOTERM_BP_DIRECT | GO:0048041~focal adhesion assembly | 7 | DLC1, ARHGAP6, RCC2, BCL2, FERMT2, TESK2, ITGA2 |
| GOTERM_BP_DIRECT | GO:0045671~negative regulation of osteoclast differentiation | 7 | MAFB, NF1, LILRB4, FSTL3, ZNF675, TLR4, TMEM178A |
| GOTERM_BP_DIRECT | GO:0042761~very long-chain fatty acid biosynthetic process | 5 | ELOVL5, ELOVL3, HACD1, ELOVL6, TECR |
| GOTERM_BP_DIRECT | GO:1900025~negative regulation of substrate adhesion-dependent cell spreading | 5 | RCC2, ACTN4, TACSTD2, AP1AR, GBP1 |
| GOTERM_BP_DIRECT | GO:0034383~low-density lipoprotein particle clearance | 4 | CD36, DGAT2, HMOX1, SCARB1 |
| GOTERM_BP_DIRECT | GO:0060972~left/right pattern formation | 4 | CLUAP1, IFT57, SNAI1, DPCD |
| GOTERM_BP_DIRECT | GO:2001020~regulation of response to DNA damage stimulus | 4 | ARMT1, IER3, NPAS2, CASP9 |
| GOTERM_BP_DIRECT | GO:0032731~positive regulation of interleukin-1 beta production | 4 | NOD2, SMAD3, HSPB1, JAK2 |
| GOTERM_BP_DIRECT | GO:0050863~regulation of T cell activation | 4 | LAT, SIT1, CCL5, PAG1 |
| GOTERM_BP_DIRECT | GO:0002576~platelet degranulation | 19 | ACTN4, FAM3C, F13A1, TMX3, F8, STXBP1, IGF1, SERPING1, SPARC, ECM1, GAS6, VCL, CD9, VWF, CD36, PPBP, VEGFA, ITIH4, CFD |
| GOTERM_BP_DIRECT | GO:0022617~extracellular matrix disassembly | 15 | KLK7, BSG, EXOC8, MMP19, MMP8, HSPG2, CDH1, MMP15, MMP14, CTSV, MMP12, MMP10, LAMB3, ADAM8, ADAM15 |
| GOTERM_BP_DIRECT | GO:0007010~cytoskeleton organization | 27 | LOR, ABLIM1, CCL2, RP2, WTIP, CD2AP, VILL, CCL7, ANK2, KRT5, APOE, TSPAN32, OBSL1, FGD6, TUBB3, TUBB4B, PLD2, ARC, ZMYM2, TNIK, CECR2, PALLD, BRWD3, SEMA6A, MAST4, CCL13, SPTBN2 |
| GOTERM_BP_DIRECT | GO:0048813~dendrite morphogenesis | 9 | RBFOX2, TMEM106B, KLF7, FYN, SEMA3A, KIDINS220, HPRT1, CACNA1A, VLDLR |
| GOTERM_BP_DIRECT | GO:0045926~negative regulation of growth | 6 | MT1L, GPC3, ALOX15B, MT1E, MT1X, MT1G |
| GOTERM_BP_DIRECT | GO:0051298~centrosome duplication | 6 | TUBGCP4, SASS6, TUBGCP6, TUBGCP5, CCP110, PKD2 |
| GOTERM_BP_DIRECT | GO:0042795~snRNA transcription from RNA polymerase II promoter | 14 | POLR2G, ASUN, SNAPC2, POLR2L, TAF5, SNAPC1, INTS2, GTF2E1, TAF13, RPAP2, GTF2A1, RPRD2, INTS7, GTF2A2 |
| GOTERM_BP_DIRECT | GO:0061333~renal tubule morphogenesis | 3 | MEF2C, COL4A1, PKD2 |
| GOTERM_BP_DIRECT | GO:0051984~positive regulation of chromosome segregation | 3 | CDC6, SMC5, RAD18 |
| GOTERM_BP_DIRECT | GO:0042992~negative regulation of transcription factor import into nucleus | 3 | CD36, NF1, SUFU |
| GOTERM_BP_DIRECT | GO:0006235~dTTP biosynthetic process | 3 | TYMS, DTYMK, CMPK2 |
| GOTERM_BP_DIRECT | GO:2000660~negative regulation of interleukin-1-mediated signaling pathway | 3 | IL1R2, IL1RN, ZNF675 |
| GOTERM_BP_DIRECT | GO:0035556~intracellular signal transduction | 59 | ZBTB33, TUFT1, NOD2, RASA4B, STAC2, CTGF, PPP1R1A, HMOX1, DLG5, RAPGEF2, PAG1, PRKCA, MAGI3, TNIK, SOCS2, GNB1L, ZP3, ARHGEF5, MLXIPL, SOCS7, PRKCH, SRPK1, MAP4K3, MAST4, TNS2, CD80, HSPB1, ASB2, CXCL1, PRKCZ, STYXL1, CARHSP1, STK17B, CXCL8, MYO9A, RAB40B, PLCL2, DGKA, STK32C, PLCL1, DGKD, DCLK2, PRKAA1, RASA4, BLNK, DVL2, GPR75-ASB3, SPSB1, NLK, SPSB2, NPR1, RPS6KA5, LAT, FYN, CD209, CDC42BPA, TGFBR3, JAK2, JAK3 |
| GOTERM_BP_DIRECT | GO:0042127~regulation of cell proliferation | 30 | TNFRSF6B, FXYD2, EID2, XIAP, CXCL3, CXCL2, EZH2, CHEK1, FES, TNFRSF4, FANCL, DHCR7, PKD2, BCL6, RAPGEF2, RBFOX2, BRAF, FA2H, TGFBR2, BIRC7, CD40, BRCA1, PURA, TNFRSF10A, PPBP, TIMELESS, FYN, JAK2, PIAS1, PLAU |
| GOTERM_BP_DIRECT | GO:0070098~chemokine-mediated signaling pathway | 14 | CXCL1, CCL2, CXCL5, CMKLR1, CXCL3, CXCL2, CXCL8, CCL5, CCL18, CCL7, CCL13, CCL23, CCR5, PPBP |
| GOTERM_BP_DIRECT | GO:0006768~biotin metabolic process | 5 | BTD, PDZD11, VNN1, SLC5A6, ACACB |
| GOTERM_BP_DIRECT | GO:0019395~fatty acid oxidation | 5 | GCDH, PPARG, CYGB, PRKAA1, POR |
| GOTERM_BP_DIRECT | GO:0070208~protein heterotrimerization | 5 | COL1A2, COL6A2, COL6A1, COL1A1, SKIL |
| GOTERM_BP_DIRECT | GO:0048167~regulation of synaptic plasticity | 8 | MEF2C, ATP2B2, HRH1, NOLC1, PLK2, HRH2, SIPA1L1, RAPGEF2 |
| GOTERM_BP_DIRECT | GO:0051384~response to glucocorticoid | 13 | TYMS, ALAD, CASP3, S100B, ADM, PTGDS, BCL2, IL1RN, SPARC, IGFBP2, MDK, CTSV, IL10 |
| GOTERM_BP_DIRECT | GO:0048843~negative regulation of axon extension involved in axon guidance | 7 | SEMA6A, PLXNA3, SEMA4F, SEMA7A, SEMA4C, SEMA4B, SEMA3A |
| GOTERM_BP_DIRECT | GO:0006596~polyamine biosynthetic process | 4 | OAZ2, OAZ3, SMOX, AZIN2 |
| GOTERM_BP_DIRECT | GO:0007167~enzyme-linked receptor protein signaling pathway | 4 | ERBB2, JAK2, JAK3, GAS6 |
| GOTERM_BP_DIRECT | GO:2000810~regulation of bicellular tight junction assembly | 4 | GPBAR1, PRKCH, GJA1, SNAI1 |
| GOTERM_BP_DIRECT | GO:0008608~attachment of spindle microtubules to kinetochore | 4 | TEX14, FAM175B, AURKC, AURKB |
| GOTERM_BP_DIRECT | GO:0006633~fatty acid biosynthetic process | 11 | SC5D, FA2H, ABCD3, CBR4, PRKAA1, HACD1, ACACB, ACSL3, BRCA1, ACSM5, HSD17B8 |
| GOTERM_BP_DIRECT | GO:0055088~lipid homeostasis | 9 | CEBPA, GCDH, ACADSB, NR1D2, ACADS, PPARG, ACAD11, PNPLA4, ACAD10 |
| GOTERM_BP_DIRECT | GO:0050729~positive regulation of inflammatory response | 14 | CCL2, ZP3, ITGA2, TLR4, GPRC5B, CCL5, CCL18, CCL7, CCL13, CCL23, ETS1, NLRP12, FABP4, JAK2 |
| GOTERM_BP_DIRECT | GO:0045444~fat cell differentiation | 14 | PID1, CEBPA, OSBPL8, FFAR4, SMAD6, SOCS1, BSCL2, SOCS7, ALMS1, CBY1, TRIM32, MKKS, PIAS1, GRK5 |
| GOTERM_BP_DIRECT | GO:0042060~wound healing | 15 | ERBB2, FUT10, TGFBR2, NF1, SMAD3, SPARC, CELSR1, IL24, CDH3, SLC11A1, CASP3, FGFR1OP2, SPRR3, PDGFRA, SCARB1 |
| GOTERM_BP_DIRECT | GO:0071526~semaphorin-plexin signaling pathway | 8 | SEMA6A, PLXNA3, SEMA4F, PLXNA2, SEMA7A, SEMA4C, SEMA4B, SEMA3A |
| GOTERM_BP_DIRECT | GO:0051604~protein maturation | 5 | TESC, ISCA2, TSPAN5, SORL1, TSPAN15 |
| GOTERM_BP_DIRECT | GO:0035329~hippo signaling | 7 | DVL2, MOB1B, CASP3, MOB1A, TEAD3, WWTR1, TJP2 |
| GOTERM_BP_DIRECT | GO:0035058~nonmotile primary cilium assembly | 7 | BBS1, KIF3A, MKKS, PIBF1, CENPJ, BBS10, ARL13B |
| GOTERM_BP_DIRECT | GO:0043029~T cell homeostasis | 7 | SIT1, BCL2, RIPK3, TNFSF14, JAK3, SLC39A3, BCL2L11 |
| GOTERM_BP_DIRECT | GO:0048661~positive regulation of smooth muscle cell proliferation | 12 | NOTCH3, RBPMS2, MNAT1, NAMPT, S1PR1, ID2, HMOX1, TGFBR2, SKP2, IGF1, ITGA2, CCL5 |
| GOTERM_BP_DIRECT | GO:1900182~positive regulation of protein localization to nucleus | 6 | CDK1, KAT7, CDKN2A, FYN, EIF2AK3, CD2AP |
| GOTERM_BP_DIRECT | GO:0001889~liver development | 14 | CEBPA, COBL, UPF2, GNPNAT1, NF1, SMAD3, AK4, ACAT1, SEC63, SOD2, ALDH1A2, IGF2R, PKD2, TGFBR3 |
| GOTERM_BP_DIRECT | GO:0042981~regulation of apoptotic process | 33 | SEPT4, GDF1, BCAR1, TNFRSF4, RABGGTB, NOD2, CASP9, PAX8, CHM, BCL6, SKIL, DLG5, ZNF420, TRAF4, LCMT1, ACTN4, MADD, LGALS1, ANP32E, SKP2, BRCA1, TNFRSF10A, RASSF3, CARD11, ETS1, FYN, IFT57, GDF11, JAK2, PPP1R13B, PIDD1, FBXO10, BMP8A |
| GOTERM_BP_DIRECT | GO:0038096~Fc-gamma receptor signaling pathway involved in phagocytosis | 7 | FYN, FCGR2B, WAS, ARPC1A, MYO10, VAV3, NCKAP1 |
| GOTERM_BP_DIRECT | GO:0006909~phagocytosis | 3 | CD36, ANXA11, SLC11A1 |
